# Supplementary material for: What are the volume and budget needs to provide chemotherapy to all children with acute lymphoblastic leukaemia in Thailand? Development and application of an estimation tool
Source: BMJ Open. 2020 Oct 26;10(10):e041901. doi: 10.1136/bmjopen-2020-041901 (PMC7592266; doi:10.1136/bmjopen-2020-041901)
Supplement: Author's manuscript [file bmjopen-2020-041901.draft_revisions.pdf]

BMJ Open is committed to open peer review. As part of this commitment we make the peer review history of every article we publish publicly available.

When an article is published we post the peer reviewers' comments and the authors' responses online. We also post the versions of the paper that were used during peer review. These are the versions that the peer review comments apply to.

The versions of the paper that follow are the versions that were submitted during the peer review process. They are not the versions of record or the final published versions. They should not be cited or distributed as the published version of this manuscript.

BMJ Open is an open access journal and the full, final, typeset and author-corrected version of record of the manuscript is available on our site with no access controls, subscription charges or pay-per-view fees (http://bmjopen.bmj.com).

If you have any questions on BMJ Open's open peer review process please email info.bmjopen@bmj.com

BMJ Open

Evidence to improve access to pharmacotherapy: A model to estimate needs, costs, and availability of medicines to treat pediatric acute lymphoblastic leukemia

Journal:	*BMJ Open*
Manuscript ID	bmjopen-2020-041901
Article Type:	Original research
Date Submitted by the Author:	23-Jun-2020

Complete List of Authors:	Sruamsiri, Rosarin; Naresuan University, Center of Pharmaceutical Outcomes Research<br>Ferrario, Alessandra; Harvard Medical School, Population Medicine; Harvard Pilgrim Health Care Institute, Population Medicine<br>Ross-Degnan, Dennis; Harvard Medical School, Department of Population Medicine; Harvard Pilgrim Health Care Institute, Department of Population Medicine<br>Denberg, Avram; Hospital for Sick Children, Child Health and Evaluative Sciences; The Hospital for Sick Children<br>Frazier, A. Lindsay; Dana-Farber/Boston Children's Cancer and Blood Disorders Center<br>Gupta, Sumit; Hospital for Sick Children, Child Health Evaluative Sciences; Hospital for Sick Children, Division of Haematology/Oncology<br>Ward, Zachary; Harvard University T H Chan School of Public Health, Center for Health Decision Science<br>Yeh, Jennifer; Harvard Medical School, Department of Pediatrics<br>Wagner, Anita; Harvard Medical School, Department of Population Medicine; Harvard Pilgrim Health Care Institute, Department of Population Medicine

Keywords:	Leukaemia < ONCOLOGY, Paediatric oncology < ONCOLOGY, Health policy < HEALTH SERVICES ADMINISTRATION & MANAGEMENT

SCHOLARONE™ Manuscripts

I, the Submitting Author has the right to grant and does grant on behalf of all authors of the Work (as defined in the below author licence), an exclusive licence and/or a non-exclusive licence for contributions from authors who are: i) UK Crown employees; ii) where BMJ has agreed a CC-BY licence shall apply, and/or iii) in accordance with the terms applicable for US Federal Government officers or employees acting as part of their official duties; on a worldwide, perpetual, irrevocable, royalty-free basis to BMJ Publishing Group Ltd ("BMJ") its licensees and where the relevant Journal is co-owned by BMJ to the co-owners of the Journal, to publish the Work in this journal and any other BMJ products and to exploit all rights, as set out in our licence.

The Submitting Author accepts and understands that any supply made under these terms is made by BMJ to the Submitting Author unless you are acting as an employee on behalf of your employer or a postgraduate student of an affiliated institution which is paying any applicable article publishing charge ("APC") for Open Access articles. Where the Submitting Author wishes to make the Work available on an Open Access basis (and intends to pay the relevant APC), the terms of reuse of such Open Access shall be governed by a Creative Commons licence – details of these licences and which Creative Commons licence will apply to this Work are set out in our licence referred to above.

Other than as permitted in any relevant BMJ Author's Self Archiving Policies, I confirm this Work has not been accepted for publication elsewhere, is not being considered for publication elsewhere and does not duplicate material already published. I confirm all authors consent to publication of this Work and authorise the granting of this licence.

For peer review only

Evidence to improve access to pharmacotherapy: A model to estimate needs, costs, and availability of medicines to treat pediatric acute lymphoblastic leukemia

Authors: Rosarin Sruamsiri[1†], Alessandra Ferrario[2*†], Dennis Ross-Degnan[2], Avram E Denburg[3,4], A Lindsay Frazier[5], Sumit Gupta[3,4], Zachary J Ward[6], Jennifer M Yeh[7], Anita K Wagner[2]

[1]Center of Pharmaceutical Outcomes Research, Naresuan University, Phitsanulok, Thailand

[2]Department of Population Medicine, Harvard Medical School and Harvard Pilgrim Health Care Institute, Boston, USA

[3]Child Health Evaluative Sciences, Hospital for Sick Children, Toronto, Canada

[4]Division of Haematology/Oncology, Hospital for Sick Children, Toronto, Canada

[5]Dana-Farber/Boston Children's Cancer and Blood Disorders Center, Boston, USA

[6]Center for Health Decision Science, Harvard TH Chan School of Public Health, Boston, USA

[7]Department of Pediatrics, Harvard Medical School, Boston, USA; Division of General Pediatrics, Boston Children's Hospital, Boston, USA

[†]Joint first authors (RS and AF contributed equally to this paper)

*Corresponding author

For peer review only

Alessandra Ferrario
Division of Health Policy and Insurance Research
Department of Population Medicine
Harvard Medical School and Harvard Pilgrim Health Care Institute
401 Park Drive, Suite 401 East
Boston, MA 02215
Email: Alessandra_Ferrario@hphci.harvard.edu
Tel. +1 617 867 4863

Key words: leukemia; pediatric oncology; health policy

Word count and reference count: 3022 words and 44 references

For peer review only

For peer review only - http://bmjopen.bmj.com/site/about/guidelines.xhtml

Abstract *(242 words)*

Objective: To develop a model to estimate the need, availability and cost of medicines required to treat children with acute lymphoblastic leukemia (ALL), the most common form of pediatric cancer.

Methods: Using national treatment guidelines for pediatric ALL, incidence data, and published local medicines prices, we estimated the volume (mg) and cost of medicines needed to treat children with ALL in Thailand. For medicines whose main indication is pediatric ALL (asparaginase and 6-mercaptopurine), we compared need with actual sales in 2017 to assess whether available quantities in the market were sufficient to cover estimated needs.

Results: Ten anti-cancer medicines and four chemoprotective agents are needed for the treatment of pediatric ALL in Thailand. Of these fourteen medicines, thirteen are included in the WHO essential medicines list for children. All are available as generics. We estimated that essential chemotherapy and chemoprotective agents to treat all children diagnosed with pediatric ALL in Thailand in 2017 would cost US$ 814 952 (US$ 1 365 422 for diagnosed and undiagnosed children), which corresponds to 0.005% (0.008%) of the country's total health expenditure. The volume of asparaginase and 6-mercaptopurine available on the Thai market in 2017 was more than sufficient (2.3 and 1.5 times, respectively the amount needed) to treat all diagnosed children with pediatric ALL.

Conclusions: Procuring sufficient quantities of essential medicines to treat children with pediatric ALL requires relatively modest resources. Medicine cost should not be a major barrier to ALL treatment in similar settings.

For peer review only

For peer review only - http://bmjopen.bmj.com/site/about/guidelines.xhtml

Strengths and limitations of this study

- Development of a model to estimate the need, availability and cost of medicines required to treat children with acute lymphoblastic leukemia that can be customized to needs of a specific country or setting and populated with local data
- Generation of evidence which can support advocacy and inclusion of essential medicines in universal health coverage benefit packages
- Medicines are an essential component of cancer treatment but not the only component needed. Improving survival requires investment in other health service components not included in this analysis.

For peer review only

For peer review only - http://bmjopen.bmj.com/site/about/guidelines.xhtml

Background

Important global and local forces have shaped the pediatric oncology landscape over the past two decades. The pediatric oncology community, together with researchers and other partners, has advocated widely for the need to address disparities in survival between countries at different levels of health system development (1–3). Global efforts to close this gap have featured the development of resource-appropriate treatment guidelines (4,5), the inclusion of key medicines to treat pediatric cancer in iterative updates of the World Health Organization's (WHO) Essential Medicines List for Children (6), the establishment of twinning programs (7,8) and global working groups like the WHO Global Initiative for Childhood Cancer (9), the development and strengthening of pediatric cancer registries and analytical efforts to provide evidence on the burden of pediatric cancer (10–14). These pediatric oncology focused endeavors have been supported by broader global commitments and efforts to achieve universal health coverage and the sustainable development agenda which, among other targets, emphasizes reduction in preventable mortality from non-communicable diseases and access to quality assured essential medicines (15,16).

At the country level, efforts have included the development and implementation of standardized protocols to treat pediatric cancer, the establishment of national cancer plans and cancer registries, and the addition of pediatric cancer medicines to national essential medicines and reimbursement lists (17), among others. The extent to which different countries have engaged in such efforts varies. Thailand has been in the vanguard of these positive global trends. In Thailand, national treatment guidelines for childhood leukemia were developed in 2005 and implemented in 2006, together with coverage for diagnostics, chemotherapy and hospital treatment by the National Health Security Office (18–20). Pediatric cancer registries are in place across the country and regular analyses of incidence and survival have been published over time (18–22). At 66%, 5-year net survival for pediatric acute lymphoblastic leukemia (the most common form of cancer in children in most regions of the world) in Thailand is higher than the global (56%), Asian (53%), South-Eastern Asian (40%) and the upper middle-income countries averages (63%) (13) (see Supplementary table 1 for more indicators).

A key component of pediatric cancer treatment is chemotherapy. Yet, despite calls for making medicines affordable and the need to address shortages affecting pediatric cancer medicines globally (1,23,24), analyses of availability of pediatric cancer medicines at the country level are limited. Previous multi-

For peer review only - http://bmjopen.bmj.com/site/about/guidelines.xhtml

country studies reviewed the inclusion of pediatric cancer medicines in national essential medicines and reimbursement lists (25) and surveyed physician and health care professionals regarding the level of access to essential pediatric chemotherapies (26). Subnational studies have assessed medicines availability and affordability at the pharmacy level (27). These are important contributions which need to be complemented by evidence on the actual volumes of medicines needed and available, and the corresponding budget required to provide a full course of treatment to all children with cancers. Further, existing supply chain vulnerabilities impacting access to quality medicines have been dramatically exposed during the COVID-19 pandemic (28,29), calling for even greater attention to availability of medicines. One example is reliance on a limited number of countries for active pharmaceutical ingredients and manufacturing (28) which puts importing countries at risk of shortages if exporting countries decide to reduce the volume of production, stockpile, or even temporarily suspend production. This has affected products used to address the pandemic but could also affect low-margin products like generic chemotherapy medicines (29) used in the treatment of childhood cancer.

The objective of our study was to develop a model to estimate needs and budgetary requirements for treating children with acute lymphoblastic leukemia (ALL) and apply it to Thailand as an example case. We populated the model with a unique set of data including the latest available estimates of diagnosed and undiagnosed children, pharmaceutical sales data and local data on cancer stage distribution. Further, we compared needed quantities with sold quantities of pediatric cancer medicines used for the treatment of ALL to identify possible unmet need. Ultimately, we aimed to generate a transferable approach that can be customized to other jurisdictions and populated with local data to estimate the need for and availability of essential pediatric cancer medicines, as well as their corresponding budget impact.

Methods

Data sources

Cancer incidence by risk group or stage

We used age-stratified (<1; 1-4, 5-9, 10-14 years old) estimates of the number of children with incident ALL, diagnosed and undiagnosed, from the Global Childhood Cancer (GCC) microsimulation model (12). These GCC estimates were calibrated against the International Incidence of Childhood Cancer data (30) and included relevant demographic, incidence and health system variables to estimate the number of

For peer review only - http://bmjopen.bmj.com/site/about/guidelines.xhtml

diagnosed and undiagnosed cases (12). As published international estimates do not sub-classify patients by risk group, we estimated the proportion of children with ALL by risk group (standard, high, relapse) using data from Thai pediatric ALL patients (19) on the proportion of children per risk group treated with standard vs. high risk protocols.

Therapeutic guidelines

We used the national treatment guidelines published in 2016 by the Thai National Health Security Office (NHSO) (31) which were developed using treatment regimens implemented in advanced health care systems. NHSO guidelines are the basis for health insurance reimbursement for approximately 80% of the Thai population. The guidelines include separate protocols for standard risk ALL, high risk ALL and for relapsed ALL (the latter with separate treatment schemes for standard and high-risk relapse). All protocols specify recommended doses of each medication by age group, risk group, and treatment phase. We identified all antineoplastic agents and chemoprotective agents in the 2016 NHSO pediatric ALL treatment guidelines (31).

Medications sold

We estimated the volumes of each medication of interest sold in Thailand from January 1, 2017 through December 31, 2017 using IQVIA (formerly IMS Health and Quintiles) data. IQVIA is a multinational company that conducts multi-sample audits of pharmaceutical purchase data based on invoices from pharmacies, wholesalers, distributors, and manufacturers in the hospital and retail sectors in a number of countries worldwide (32). The proprietary data are extrapolated to represent national level sales. Quality checks (e.g. comparison with manufacturer data) are conducted to ensure accuracy and representativeness of the data (32). IQVIA sales data record quarterly sales volumes of each product with information on formulation, strength, number of units per pack and number of packs sold. We used this information to calculate the total volume, in milligrams (mg), of each active ingredient sold in 2017. We expressed volume of medicines sold as the number of smallest units for the relevant formulation (i.e. the one recommended in the Thai pediatric ALL guidelines) of each international nonproprietary name (INN). We defined the smallest unit as the unit with the smallest amount (mg or active units) of active ingredient for the relevant formulation listed in the 2019 WHO model list of essential medicines for children (6).

For peer review only - http://bmjopen.bmj.com/site/about/guidelines.xhtml

Prices

We extracted price data from the Pharmaceutical Information Center, Ministry of Public Health, Thailand (33). These represent list prices and may be higher than the prices actually paid. As such, they provide a conservative estimate of the maximum price paid.

Analyses

Pediatric ALL medication need

Building on earlier work (2014) by some of the authors (RS, DRG, AKW and ALF), we have updated a model that we created to forecast medicines' need. We used data on weight and height of Thai children by age (34) to calculate the average body surface area for each age group according to the Mosteller formula (35). We multiplied the number of patients in each age and risk-group by the milligrams (or active units) per body surface area needed, for each ALL medication, according to the treatment protocols. We then summed the number of mg needed across different age and risk groups to arrive at a total number of milligrams for each antineoplastic and chemoprotective agents needed to treat both diagnosed and undiagnosed children with ALL in Thailand. We present the volume of ALL medicines needed as the number of smallest units.

Comparing volumes needed and volumes sold

Of the ten anticancer medicines included in the pediatric ALL protocols, most are also used for high-incidence adult cancers like breast cancer and lung cancer. Only two medicines, asparaginase and 6-mercaptopurine, are used predominantly for pediatric ALL, with a relatively small proportion of the total volume used for adolescents and young adults with ALL. In Thailand, asparaginase is a minor agent in treatment protocol for acute myeloid leukemia (in 2017 there were 97 children diagnosed with this type of cancer in Thailand). For these two medications, we compared the smallest units of both estimated needs and national sales volumes between January and December 2017.

Expenditure

Using price and volume data, we calculated the budget needed to purchase antineoplastic and chemoprotective agents for diagnosed only and diagnosed and undiagnosed children in 2017 and 2021.

For peer review only - http://bmjopen.bmj.com/site/about/guidelines.xhtml

Prices were adjusted for inflation (36). For the 2017 estimates, we calculated what percentage of the national health care expenditure (37) was required to pay for these medicines.

We conducted sensitivity analyses by varying the proportion of children who relapse and the protocol on which they relapse (standard vs. high risk) and by using the upper bound of the estimate of the number of children with ALL (12). Data preparation was conducted in Excel and the final analysis was conducted in Stata 15.1 (StataCorp LLC).

This study was approved by the Harvard Pilgrim Health Care Institute Institutional Review Board. The funders of this study had no role in study design, collection, analysis, or interpretation of the data, or writing of the report. The corresponding authors had full access to all data and the final responsibility to submit for publication. We used the STROBE reporting guidelines for Observational studies in epidemiology (38) to guide the writing of this study.

Patient and public involvement

Patients or the public were not involved in the design, or conduct, or reporting, or dissemination plans of our research.

Results

Based on the Global Childhood Cancer microsimulation model, there were an estimated 318 (95% uncertainty interval 197-434) children 0-14 years old with diagnosed ALL and 215 children with undiagnosed ALL in Thailand in 2017, for a total to 533 (95% uncertainty interval 296-795) children with pediatric ALL (Table 1). Children who develop ALL from age 10-14 are considered high risk, so all children in this age group were allocated to the high-risk protocol. Based on published evidence of stage distribution and relapse (19), 65% of 0-9 year old children were categorized as standard-risk and 35% as high-risk. Further, we estimated that 69 diagnosed children (22%) would relapse. Of these relapses, we estimated that 28% would be treated on the standard-risk relapse protocol and 72% on the high-risk relapse protocol based on evidence from previous local studies on immunotype and site of relapse (19,21), risk allocation according to the national treatment guidelines (31) and assuming equal distribution of very early, early and late relapse.

For peer review only - http://bmjopen.bmj.com/site/about/guidelines.xhtml

<Table 1: Children with ALL by age and risk group in Thailand in 2017 and 2021>

<Table 2: Children with ALL who relapse by risk-group in Thailand in 2017 and 2021>

Ten anti-cancer medicines and four chemoprotective agents are employed in the treatment of pediatric ALL in Thailand (Table 3). Thirteen of these fourteen medicines are included in the WHO essential medicines list for children and all medicines are available as generics. Providing these medicines to all diagnosed children would cost an estimated US$ 814,952 (US$ 1,365,422 including undiagnosed children), which represents 0.005% (0.008% including undiagnosed) of total health expenditures in Thailand in 2017 (Table 2). Five medicines would account for more than 90% of total expenditures (asparaginase 28%, methotrexate 26%, 6-mercaptopurine 20%, cytarabine 9% and vincristine 9%). For medicines used almost exclusively for the treatment of pediatric ALL, asparaginase and 6-mercaptopurine, the volume sold is more than sufficient (2.3 and 1.5 times, respectively the amount needed for ALL) to treat all children diagnosed with ALL, including children who relapse.

<Table 3: Medications for pediatric ALL – volumes needed, estimated cost in 2017 and 2021 and actual sales in 2017>

Sensitivity analysis showed that even if the number of children with pediatric ALL is 27% higher (upper bound of 95% uncertainty interval), or if 50% of children relapse, or if the ratio of standard vs. high risk relapse is 60% greater, the total expenditure required to treat diagnosed and undiagnosed children would still be less than 0.02% of total health expenditure (see supplementary material).

Discussion

In this study, we contribute to global and local efforts to improve pediatric cancer treatment by developing a model to estimate the volume of medicines needed and available, and the corresponding budget required to implement national treatment protocols for pediatric ALL. We find that providing essential medicines to all children with pediatric ALL (diagnosed and undiagnosed) in Thailand would cost US$ 1,365,422 which corresponds to less than 0.01% of the country’s 2017 health care expenditure. To help inform planning going forward, we also estimated volume and budgetary requirements for 2021 and found that accounting for inflation and changes in incidence, the expected budget impact of pediatric ALL antineoplastic and chemoprotective medications is US$ 1,421,880 (diagnosed and

For peer review only - http://bmjopen.bmj.com/site/about/guidelines.xhtml

undiagnosed). Treating the most common form of pediatric cancer thus requires a relatively modest investment. The strengths of our model are that it is based on local treatment protocols, local incidence estimates of both diagnosed and undiagnosed children, and local medicines prices and sales; the model can readily be customized for use by other countries who populate it with their own data.

Access to essential pediatric oncology medicines is a long-standing (1) and complex issue. Certain barriers to access disproportionately impact countries with developing health systems, such as limited affordability, inefficient procurement practices, supply chain weaknesses, and poor quality medicines (39). However, countries at all levels of health system development are affected by recurrent shortages of essential pediatric antineoplastic medicines (40). These shortages are due to a range of factors including thin profit margins for generic antineoplastics, interruptions in sole source production due to issues in the manufacture of sterile injectables, and poor forecasting of need (41). Shortages introduce risks of sub-optimal treatment, due to treatment interruption, substitution, and increased use of substandard or falsified medicines procured reactively through available alternative suppliers (42,43). Availability challenges have been amplified during the COVID-19 pandemic. It is therefore very timely to reinforce the importance to address them and use models to estimate procurement volumes and budgets (44).

A national essential medicines list linked with standard treatment guidelines and universal health coverage have been identified as keys to improved access to pediatric cancer treatment and ultimately health outcomes. Given the imperative of using scarce resources wisely on the path to universal health coverage, it is important to know the extent of treatment needs and expected budget impact. Accurate forecasts of need can help increase procurement efficiency. If procurement is aggregated across different hospitals, it can generate economies of scale (44). Knowledge of the types of medicines needed and volumes required can enable measures to proactively prevent or mitigate the impact of anticipated or recurrent shortages.

There are limitations to our study. Our model includes some assumptions. We assume that all children complete the full first course of treatment and that those who relapse complete the full relapse protocol. We made these assumptions because we wanted to estimate the volume and budget that would be needed if every child received a full treatment course. In reality, some children will relapse, die

For peer review only - http://bmjopen.bmj.com/site/about/guidelines.xhtml

from ALL or treatment toxicity, or abandon therapy during their treatment course. We also did not account for wastage due to larger-than-needed vial sizes. The Thai guidelines include separate treatment regimens for low risk infants and intermediate/high risk infants. Given that less than 4% of children with ALL in Thailand were infants (<1 year old) we included them in the 0-4 year old group, which was assigned proportions of 0.65 and 0.35 to either the standard or high risk protocols, respectively. The distributions of risk status and assigned treatment protocol were based on assumptions informed by local data. Sensitivity analyses varying these distributions did not change our overall conclusions. The IQVIA sales data do not capture medications obtained through special procurement channels, such as donated products. We may therefore have underestimated the available volumes of some products. However, as we found no gaps in availability, this would not affect our finding of sufficient amounts of the pediatric cancer medicines we studied in Thailand at the time of the study. Finally, while availability of medicines is an essential component of effective pediatric cancer treatment, improving outcomes of care requires other health systems inputs (e.g. pediatric oncologists and nurses) and processes (e.g. diagnostic capacity, referrals, access to specialist care, ability to prevent and treat side effects). Global childhood cancer 5-year survival rates are projected to improve from 37% to ≥50% with expanded treatment access or improved service delivery compared with only to 42% with improved access to chemotherapy (13). That is, equitable access to appropriate medicines is a necessary but not sufficient component of a health system equipped to treat children with cancers.

Conclusions

The unacceptable survival disparities among children with cancer between high- and low- and middle-income countries have been recognized as an injustice which demands redress. Medicines are an important component in the set of interventions needed to bridge the gap in global 5-year net survival for childhood cancer which ranges from 12% in eastern Africa, 40% in South-East Asia, to 90% in Western and Northern Europe, North America, Australia and New Zealand (13). The introduction and expansion of universal health coverage in low- and middle-income countries provides an opportunity to save lives by including pediatric cancer medicines in benefit packages. To do so requires sound, context-specific data on required volumes and expected costs of pediatric cancer medications.

In this study, we developed a model to generate such evidence that can be applied and customized to the needs and circumstances of different countries. This evidence can be used both for advocacy as well

For peer review only - http://bmjopen.bmj.com/site/about/guidelines.xhtml

as to inform the procurement process with the aim of increasing efficiency. Ultimately, evidence-based estimates of needs, together with market analysis, can help countries move from passive purchasing based on historical data to strategic procurement and proactive mitigation of potential shortages.

Acknowledgments

This work would not have been possible without the numerous contributions from Dr. Peter Stephens from IQVIA, United Kingdom. We gratefully acknowledge Dr. Stephens and the IQVIA for providing the sales data and information on medicines' sales data. We gratefully acknowledge the contribution of Prof. Dr. Suradej Hongeng, Department of Pediatrics, Faculty of Medicine, Ramathibodi Hospital, Mahidol University, Bangkok, Thailand in providing important information on the treatment of pediatric ALL in Thailand. We gratefully acknowledge the Global Task Force on Expanded Access to Cancer Care and Control, led by Dr. Felicia Knaul, which provided funding for the initial development in 2014 of the approach to estimate gaps between needs for and availability of medications to treat children with cancers.

Contributors

RS and AF contributed equally to this paper. RS, DRG and AW with contribution from ALF developed a model to estimate need and availability as part of the POEMS project in 2014. The model was updated and enhanced between 2019 and 2020 by AF and RS who also compiled the data and conducted the analysis. SG, AD and ALF advised on clinical aspects. JY and ZW advised on analytical aspects. AF wrote the first draft of the paper; all authors contributed the revisions of the manuscript to its final version and approved the final version.

Funding

The initial development of a model (POEMS project 2014) underlying this project was funded by the Global Task Force for Cancer Care and Control, an initiative promoted by the Harvard Global Equity Initiative, Harvard Medical School, Harvard T.H. Chan School of Public Health, Dana-Farber Cancer Institute, Fred Hutchinson Cancer Research Center, and University of Washington School of Medicine. At that time, RS was a Research Fellow in the Department of Population Medicine and supported by the Thai Royal Golden Jubilee PhD program (grant number PHD/0127/2552). There were no external funds

For peer review only - http://bmjopen.bmj.com/site/about/guidelines.xhtml

provided for the present analyses. AF was supported by a postdoctoral Fellowship from the Swiss National Science Foundation and AKW by a Department of Population Medicine Ebert Award, Harvard Medical School and Harvard Pilgrim Health Care Institute.

Competing interests

RS is currently employed by Takeda (Thailand) Ltd. AF reports personal fees from the European Society of Medical Oncology, separate from the submitted work. AKW reports other from Global Task Force for Cancer Care and Control, during the initial development of the model. The other authors declare no competing interests.

Patient and public involvement

Patients or the public were not involved in the design, or conduct, or reporting, or dissemination plans of our research.

Ethics approval

This study was approved by the Harvard Pilgrim Health Care Institute Institutional Review Board.

Data availability statement

All data relevant to the study are included in the article or uploaded as supplementary information.

For peer review only

Tables

Table 1: Estimated numbers (%) of Thai children aged 0-14 years with ALL in 2017 and 2021

	Diagnosed								Diagnosed and undiagnosed							
	Standard risk				High risk				Standard risk				High risk			
	n	LB	UB	%	n	LB	UB	%	n	LB	UB	%	n	LB	UB	%
**2017**																
Age group																
0-4	95	58	128	29.8	51	32	70	16.1	159	87	238	29.8	86	47	129	16.1
5-9	68	43	92	21.3	37	23	50	11.5	113	64	169	21.3	61	34	92	11.5
10-14	0	0	0	0	68	41	94	21.3	0	0	0	0.0	113	64	167	21.2
Total by risk group	162	101	221	51.1	155	96	213	48.9	272	151	408	51.1	261	145	387	48.9
Total by diagnosis status	318	197	434	100					533	296	795	100				
**2021**																
0-4	86	51	120	29.0	47	28	65	15.7	144	78	219	29.0	78	42	118	15.7
5-9	64	42	90	21.6	35	22	48	11.7	108	62	163	21.8	59	33	88	11.8
10-14	0	0	0	0.0	65	40	91	21.9	0	0	0	0.0	108	59	161	21.7
Total by risk group	150	93	210	50.7	146	90	204	49.3	252	140	382	50.8	244	134	367	49.2
Total by diagnosis status	297	183	414	100					496	274	749	100				

Notes: LB: lower bound; UB: upper bound of 95% uncertainty interval (calculated as the 2.5 and 97.5 percentiles). Numbers (n, LB, UB) were rounded to the nearest full digit.

For peer review only

For peer review only - http://bmjopen.bmj.com/site/about/guidelines.xhtml

Sources: Number of incident pediatric ALL cases (diagnosed, all) by age group (12), risk group proportions for children (19).

Table 2: Estimated numbers (%) of Thai children with ALL who relapse by risk-group in 2017 and 2021

	Diagnosed								Diagnosed and undiagnosed							
	Standard risk				High risk				Standard risk				High risk			
	n	LB	UB	%	n	LB	UB	%	n	LB	UB	%	n	LB	UB	%
**2017**																
Total relapse by risk group*	19	12	26	28.0	50	31	68	72.0	32	18	48	28.0	83	46	124	72.0
Total relapse by diagnosis status	69	43	94	100					116	64	172	100				
**2021**																
Total relapse by risk group*	18	11	25	28.0	46	29	65	72.0	30	17	46	28.0	78	43	117	72.0
Total relapse by diagnosis status	64	40	90	100					108	59	162	100				

Notes: LB: lower bound; UB: upper bound of 95% uncertainty interval (calculated as the 2.5 and 97.5 percentiles). Risk classification for relapse treatment is independent of the original risk classification for the first full course of treatment. There is a second risk assessment for children who relapse. Numbers (n, LB, UB) were rounded to the nearest full digit.

Sources: Number of incident pediatric ALL cases (diagnosed, all) by age group (12), risk group proportions for children (19,21).

For peer review only

Table 3: Medications for pediatric ALL – estimated volumes needed, cost, and actual sales in Thailand in 2017 and 2021

			2017					2021			
			Total smallest units		Expenditure (US$)			Total smallest units		Expenditure (US$)	
	Route	Smallest unit	DX	DX + uDX	DX	DX + uDX	Percentage	DX	DX + uDX	DX	DX + uDX
Asparaginase	IM	10000	4,607 [10,365]	7,719	225,847	378,394	27.7	4,348	7,263	236,209	394,571
Methotrexate	*all*				213,031	356,925	26.1			222,654	371,993
Methotrexate	IV	50	83,180	139,364	*190,358*	*318,934*		78,482	131,109	*199,042*	*332,511*
Methotrexate	oral	2.5	235,036	393,798	*19,285*	*32,311*		221,110	369,701	*20,105*	*33,617*
Methotrexate	IT	10	6,730	11,281	*3,388*	*5,680*		6,285	10,512	*3507*	*5865*
Mercaptopurine	oral	50	223,501 [340,035]	374,472	164,313	275,303	20.2	210,281	351,584	171,324	286,448
Cytarabine	IV	100	18,905	31,674	73,572	123,268	9.0	17,798	29,752	76,763	128,319
Vincristine	IV	1	17,363	29,092	70,469	118,070	8.6	16,341	27,320	73,507	122,891
Tioguanine	oral	40	5,429	9,096	12,011	20,125	1.5	5,107	8,539	12,522	20,938
Mitoxantrone	IV	10	112	188	11,586	19,413	1.4	105	176	12,081	20,199
Doxorubicin	IV	10	3,401	5,698	9,705	16,260	1.2	3,211	5,363	10,155	16,961
Cyclophosphamide	IV	500	1,386	2,322	6,551	10,976	0.8	1,309	2,186	6,854	11,448
Etoposide	IV	100	375	628	2,112	3,538	0.3		589	2,202	3,681
Leucovorin	IV	30	3,438	5,761	21,030	35,236	2.6	3,235	5,409	21,928	36,663
MESNA	IV	400	822	1,378	2,565	4,298	0.3	780	1,301	2,401	4,004
Prednisolone	oral	5	183,024	306,654	1,863	3,122	0.2	172,180	287,889	1,943	3,249
Dexamethasone	oral	2	21,048	35,265	295	494	0.0	19,801	33,107	307	514
Antineoplastic agents					789,198	1,322,272				824,270	1,377,449
Chemoprotective agents					25,754	43,149				26,579	44,430

For peer review only

For peer review only - http://bmjopen.bmj.com/site/about/guidelines.xhtml

Total expenditure on antineoplastic and chemoprotective agents	814,952	1,365,422	850,849	1,421,880
Total health expenditure (in million constant (2017) US$)	17,055			
Percentage of total health budget	0.005	0.008		

Notes: Route of administration: IM: intramuscular; IV: intravenous; mg: milligrams; [volume sold] is provided for medicines used mainly for the treatment of pediatric ALL. DX: diagnosed; DX + uDX: diagnosed and undiagnosed

Source: Authors' estimation based on Thai ALL treatment protocol (31), incidence data (12), IQVIA sales data, WHO Global Health Expenditure Database (37).

For peer review only

For peer review only - http://bmjopen.bmj.com/site/about/guidelines.xhtml

Supplementary material

Key indicators in Thailand in comparison to regional, upper middle income and global averages

Incidence by age and risk group

ALL treatment protocol

Body surface area

Price of medicines

Sensitivity analysis

For peer review only

For peer review only - http://bmjopen.bmj.com/site/about/guidelines.xhtml

References

1. Pui C-H, Schrappe M, Masera G, Nachman J, Gadner H, Eden OB, et al. Ponte di Legno Working Group: statement on the right of children with leukemia to have full access to essential treatment and report on the Sixth International Childhood Acute Lymphoblastic Leukemia Workshop. Leukemia. 2004 Jun;18(6):1043–53.

2. Magrath I, Steliarova-Foucher E, Epelman S, Ribeiro RC, Harif M, Li C-K, et al. Paediatric cancer in low-income and middle-income countries. Lancet Oncol. 2013 Mar;14(3):e104-116.

3. Atun R, Bhakta N, Denburg A, Frazier AL, Friedrich P, Gupta S, et al. Sustainable care for children with cancer: a Lancet Oncology Commission. Lancet Oncol. 2020 Apr 1;21(4):e185–224.

4. Hunger SP, Sung L, Howard SC. Treatment strategies and regimens of graduated intensity for childhood acute lymphoblastic leukemia in low-income countries: A proposal. Pediatr Blood Cancer. 2009 May;52(5):559–65.

5. Yeoh AEJ, Tan D, Li C-K, Hori H, Tse E, Pui C-H, et al. Management of adult and paediatric acute lymphoblastic leukaemia in Asia: resource-stratified guidelines from the Asian Oncology Summit 2013. Lancet Oncol. 2013 Nov;14(12):e508-523.

6. WHO Model Lists of Essential Medicines [Internet]. World Health Organization. World Health Organization; [cited 2020 Mar 25]. Available from: http://www.who.int/medicines/publications/essentialmedicines/en/

7. Rodriguez-Galindo C, Friedrich P, Alcasabas P, Antillon F, Banavali S, Castillo L, et al. Toward the Cure of All Children With Cancer Through Collaborative Efforts: Pediatric Oncology As a Global Challenge. J Clin Oncol Off J Am Soc Clin Oncol. 2015 Sep 20;33(27):3065–73.

8. Hopkins J, Burns E, Eden T. International twinning partnerships: An effective method of improving diagnosis, treatment and care for children with cancer in low-middle income countries. J Cancer Policy. 2013 Mar 1;1(1):e8–19.

9. Global Initiative for Childhood Cancer [Internet]. World Health Organization. World Health Organization; [cited 2020 Mar 25]. Available from: http://www.who.int/cancer/childhood-cancer/en/

10. Bhakta N, Force LM, Allemani C, Atun R, Bray F, Coleman MP, et al. Childhood cancer burden: a review of global estimates. Lancet Oncol. 2019 Jan 1;20(1):e42–53.

11. Global Burden of Disease Cancer Collaboration, Fitzmaurice C, Allen C, Barber RM, Barregard L, Bhutta ZA, et al. Global, Regional, and National Cancer Incidence, Mortality, Years of Life Lost, Years Lived With Disability, and Disability-Adjusted Life-years for 32 Cancer Groups, 1990 to 2015: A Systematic Analysis for the Global Burden of Disease Study. JAMA Oncol. 2017 Apr 1;3(4):524–48.

12. Ward ZJ, Yeh JM, Bhakta N, Frazier AL, Atun R. Estimating the total incidence of global childhood cancer: a simulation-based analysis. Lancet Oncol. 2019 Apr;20(4):483–93.

For peer review only - http://bmjopen.bmj.com/site/about/guidelines.xhtml

13. Ward ZJ, Yeh JM, Bhakta N, Frazier AL, Girardi F, Atun R. Global childhood cancer survival estimates and priority-setting: a simulation-based analysis. Lancet Oncol. 2019 Jul 1;20(7):972–83.

14. Miranda-Filho A, Piñeros M, Ferlay J, Soerjomataram I, Monnereau A, Bray F. Epidemiological patterns of leukaemia in 184 countries: a population-based study. Lancet Haematol. 2018 Jan 1;5(1):e14–24.

15. United Nations. Sustainable Development Goals [Internet]. [cited 2019 Jan 12]. Available from: https://www.un.org/sustainabledevelopment/

16. Political Declaration of the High-level Meeting on Universal Health Coverage "Universal health coverage: moving together to build a healthier world" [Internet]. United Nations General Assembly; 2019 [cited 2020 Mar 26]. Available from: https://www.un.org/pga/73/wp-content/uploads/sites/53/2019/07/FINAL-draft-UHC-Political-Declaration.pdf

17. Chivukula MV, Tisocki K. Essential cancer medicines in the national lists of countries of the WHO South-East Asia Region: a descriptive assessment. WHO South-East Asia J Public Health. 2018;7(2):90–8.

18. Kanjanapongkul S. Outcome of acute lymphoblastic leukemia treatment using national protocols at the Queen Sirikit National Institute of Child Health. J Med Assoc Thai. 2014 Jun;97 Suppl 6:S1-5.

19. Seksarn P, Wiangnon S, Veerakul G, Chotsampancharoen T, Kanjanapongkul S, Chainansamit S-O. Outcome of Childhood Acute Lymphoblastic Leukemia Treated Using the Thai National Protocols. Asian Pac J Cancer Prev. 2015;16(11):4609–14.

20. Tharnprisan P, Khiewyoo J, Sripraya P, Wiangnon S. Relapse-free rate with childhood acute lymphoblastic leukemia treated under the thai national protocol. Asian Pac J Cancer Prev APJCP. 2013;14(2):1127–30.

21. Surapolchai P, Anurathapan U, Sermcheep A, Pakakasama S, Sirachainan N, Songdej D, et al. Long-Term Outcomes of Modified St Jude Children's Research Hospital Total Therapy XIIIB and XV Protocols for Thai Children With Acute Lymphoblastic Leukemia. Clin Lymphoma Myeloma Leuk. 2019 Aug;19(8):497–505.

22. Wiangnon S, Jetsrisuparb A, Komvilaisak P, Suwanrungruang K. Childhood cancer incidence and survival 1985-2009, Khon Kaen, Thailand. Asian Pac J Cancer Prev APJCP. 2014;15(18):7989–93.

23. Denburg A, Arora B, Arora RS, Auste C, Bagai P, Barr R, et al. Access to essential medicines for children with cancer: a joint SIOP-CCI position statement. Lancet Oncol. 2017 Jan;18(1):20–2.

24. Mehta PS, Wiernikowski JT, Petrilli JAS, Barr RD, Working Group on Essential Medicines of the Pediatric Oncology in Developing Countries committee of SIOP. Essential medicines for pediatric oncology in developing countries. Pediatr Blood Cancer. 2013 May;60(5):889–91.

25. Barr R, Robertson J. Access to Cytotoxic Medicines by Children With Cancer: A Focus on Low and Middle Income Countries. Pediatr Blood Cancer. 2016 Feb;63(2):287–91.

For peer review only

For peer review only - http://bmjopen.bmj.com/site/about/guidelines.xhtml

26. Cohen P, Friedrich P, Lam C, Jeha S, Metzger M, Qaddoumi I, et al. Global Access to Essential Medicines for Childhood Cancer: A Cross-Sectional Survey. - PubMed - NCBI. 2018;4(Dec):1–11.

27. Faruqui N, Martiniuk A, Sharma A, Sharma C, Rathore B, Arora RS, et al. Evaluating access to essential medicines for treating childhood cancers: a medicines availability, price and affordability study in New Delhi, India. BMJ Glob Health [Internet]. 2019 Apr 23 [cited 2020 Mar 26];4(2). Available from: https://www.ncbi.nlm.nih.gov/pmc/articles/PMC6509613/

28. USP. USP Global Public Policy Position. Key Elements to Building a More Resilient Supply Chain [Internet]. The United States Pharmacopeia; [cited 2020 May 28]. Available from: https://www.usp.org/sites/default/files/usp/document/our-impact/covid-19/global-policy-supply-chain.pdf

29. GlobalData Healthcare. Governments can avoid potential cancer drug shortages amid Covid-19 pandemic [Internet]. 2020 [cited 2020 May 31]. Available from: https://www.pharmaceutical-technology.com/comment/cancer-drug-shortages-covid-19/

30. Steliarova-Foucher E, Colombet M, Ries L, Hesseling P, Moreno F, Shin H, et al. International Incidence of Childhood Cancer, volume III (electronic version) [Internet]. Lyon, France: International Agency for Research on Cancer; 2017 [cited 2020 Feb 25]. Available from: http://iicc.iarc.fr/results

31. Cancer Treatment Guidelines - National protocol for the treatment of childhood cancers 2016 [Internet]. The Thai Pediatric Oncology Group; The Thai Society of Hematology; National Health Security Office; [cited 2019 Aug 9]. Available from: https://www.nhso.go.th/FrontEnd/page-information_detail.aspx?ContentID=NjAwMDAwMjY5

32. IQVIA Quality Assurance [Internet]. Frankfurt am Main: IQVIA; [cited 2020 Feb 14]. (acts 2018). Available from: https://www.iqvia.com/-/media/iqvia/pdfs/library/publications/acts-2018-32nd-edition.pdf?_=1581584888234

33. Drug and Medical Supply Information Center, Ministry of Public Health. Average medicine prices [Internet]. [cited 2020 May 20]. Available from: http://dmsic.moph.go.th/

34. [Report on the Thai population Health: 2000. Ministry of Public Health] (in Thai) [Internet]. [cited 2020 May 9]. Available from: https://www.hiso.or.th/hiso/picture/reportHealth/report/report6.pdf

35. Mosteller R. Simplified Calculation of Body-Surface Area. N Engl J Med. 1987 Oct 22;317(17):1098–1098.

36. Thailand: Inflation rate from 2004 to 2021 [Internet]. Statista. Available from: https://www.statista.com/statistics/332274/inflation-rate-in-thailand/

37. Global Health Expenditure Database [Internet]. World Health Organization. [cited 2020 Apr 10]. Available from: https://apps.who.int/nha/database

For peer review only - http://bmjopen.bmj.com/site/about/guidelines.xhtml

38. EQUATOR Network. The Strengthening the Reporting of Observational Studies in Epidemiology (STROBE) Statement: guidelines for reporting observational studies [Internet]. [cited 2020 Jun 19]. Available from: https://www.equator-network.org/reporting-guidelines/strobe/

39. Silverman R, Keller J, Glassman A, Chalkidou K. Tackling the Triple Transition in Global Health Procurement [Internet]. Washington, DC: Center for Global Development; 2019 [cited 2020 May 28]. Available from: https://www.cgdev.org/better-health-procurement

40. Unguru Y, Bernhardt MB, Berg SL, Johnson L-M, Pyke-Grimm K, Woodman C, et al. Chemotherapy and Supportive Care Agents as Essential Medicines for Children With Cancer. JAMA Pediatr. 2019 01;173(5):477–84.

41. WHO Executive Board. Addressing the global shortages of medicines, and the safety and accessibility of children's medication [Internet]. Geneva: World Health Organization; 2015 Dec [cited 2020 Jun 1]. (138th session). Available from: https://apps.who.int/iris/bitstream/handle/10665/250709/B138_41-en.pdf;jsessionid=7060D7B9E132A783D8027DAD372DC812?sequence=1

42. Dandan N. Shortage of Critical Leukemia Drug Threatens Ill Children [Internet]. 2017 [cited 2020 May 20]. Available from: https://www.sixthtone.com/news/1000776/shortage-of-critical-leukemia-drug-threatens-ill-children

43. Pisani E, Nistor A, Hasnida A, Parmaksiz K, Xu J, Kok M. Identifying market risk for substandard and falsified medicines: an analytic framework based on qualitative research in China, Indonesia, Turkey and Romania. Wellcome Open Research [Internet]. 4(70). Available from: https://wellcomeopenresearch.org/articles/4-70/v1

44. Ferrario A, Kanavos PG, Humbert T, Iwamoto K, Bak Pedersen H. Challenges and opportunities in improving access to medicines through efficient public procurement in the WHO European Region. Copenhagen: World Health Organization Regional Office for Europe; 2016.

For peer review only

For peer review only - http://bmjopen.bmj.com/site/about/guidelines.xhtml

Key indicators in Thailand in comparison to regional, upper middle income and global ave

Indicator
Population (million)[1], 2018
Percentage of the population ages 0-14[1], 2018
GDP per capita, PPP (current international $)[1], 2018
Income share held by highest 20%[1], 2018
Current health expenditure per capita, PPP (current international $)[1], 2017
omestic general government health expenditure (as percentage of current health expenditure)[1], 201
Year UHC started to be implemented
Five-year net survival ALL, 2015-2019[3]

Notes: *Comparison are made where relevant

Sources: [1]World Bank Open Data (https://data.worldbank.org/, accessed 13 April 2020); [2]Global Hea

For peer review only

erages*

Thailand	East Asia & Pacific (East Asia & Pacific excluding high income)	Upper middle income	Global
69	-	-	-
17%	20% (20%)	21%	26%
19,051.3	19,329.8 (16,113.5)	19,028.9	17,948.4
44.00%	-	-	-
670.88	975.21 (647.38)	964.5	1,355.60
76.10%	68.2% (56.8%)	54.60%	74.30%
2001	-	-	-
66.50%	52.6% (Asia) 39.7% (South-East Asia)	63.20%	56.10%

alth Expenditure Database (WHO); [3](Ward et al. Lancet Oncology 2019)

For peer review only

Calculation of number of children with ALL by risk group

1) Calculation of the number of children with ALL

Mean estimate

2017	<1	1-4	5-9	10-15
Diagnosed	11	135	104	68
Diagnosed and undiagnosed	19	226	175	113

2021	<1	1-4	5-9	10-15
Diagnosed	10	123	99	65
Diagnosed and undiagnosed	17	205	167	108

Notes: 95% uncertainty interval calculated as the 2.5 and 97.5 percentiles
Source: Ward et al. Lancet Oncology, 2019 https://doi.org/10.7910/DVN/CTJN63

2) Calculatation of children by risk group

All children age 10-15 are classified as high risk. Question: what is the proportion of children age
1. Derive proportion of Thai children with ALL who are 0-9 vs. 10-15

Proportion 0-9	0.79
Proportion 10-15	0.21

2. Based on the above proportion, estimate the number of children in the Seksarn et al. 2015 pa

Number 0-9	376
Number 10-15	102

3. Given that all 10-19 year olds are high risk and that 234 patients in the Seksarn paper were h

Number low risk in Seskarn (all 0-9 years old - 0-9 years
Number high risk in Seskarn (all 10-15 years old children

4. Therefore we can now estimate the proportion of 0-9 year olds that are high risk:

5. Thus, in the modeling, all 10-19 year olds are assigned to the high risk treatment. O assigned to standard risk treatment

2017 DIAGNOSED

Age group	1<br>0-4	2<br>5-9	3<br>10-14
**Standard risk**	95	68	0
**High risk**	51	37	68

2021

	1<br>0-4	2<br>5-9	3<br>10-14
**Standard risk**	86	64	0
**High risk**	47	35	65

Calculation of number of children who relapse by risk group

1. According to (Bunyatisai et al. 2019; Seksarn et al. 2015) on average 22% of childre

For peer review only

2. Estimate the proportion of children who relapse on the standard risk protocol vs. hi

	Surapolchai et al. 2019 (2	ksarn et al. 20	rtion across studies	
a) Immunotype				
-pre-B lineage/ B cell	138	309	0.87	
-T-cell lineage	14	51	0.13	
Relapse	15	95		
b) Site of relapse				
Isolated extramedullary	0.333	0.305	0.31	
- Central nervous system (CNS)	4	28		
- Testis	1	1		
Isolated marrow	0.467	0.526	0.52	
- Bone marrow (BM)	7	50		*
Combined	0.143	0.168	0.17	
- CNS+BM	2	15		
- BM + testis		1		
Other relapse sites	1			

2017	DIAGNOSED		
Age group	1	2	3
	**0-4**	**5-9**	**10-14**
**Total number of relapses**	32	23	15
**Standard risk**	9	6	4
**High risk**	23	16	11

2021	DIAGNOSED		
Age group	1	2	3
	**0-4**	**5-9**	**10-14**
**Total number of relapses**	29	21	14
**Standard risk**	8	6	4
**High risk**	21	15	10

Note: The risk group to which children who relapse are allocated is independent of the risk grou

Data sources	Accessed	
Ward et al. 2019	5-Jun-20	https://doi.org/10.7910/DVN/CTJN63
Seksarn et al. 2015	5-Jun-20	http://dx.doi.org/10.7314/APJCP.2015.16.

For peer review only

Bunyatisai et al. 2019	5-Jun-20	https://he01.tci-thaijo.org/index.php/jtaro/
Thai ALL protocols 2016	5-Jun-20	https://www.nhso.go.th/FrontEnd/page-inf

For peer review only

Lower bound of 95% uncertainty interval (calculated as the 2.5 ar

Total
318
533

2017	<1	1-4	5-9
Diagnosed	5	85	66
Diagnosed and	7	127	98

Total
297
496

2021	<1	1-4	5-9
Diagnosed	4	75	64
Diagnosed and	7	113	95

ed 0-9 who are high risk?

aper (N=478) who are 0-9 vs. 10-15

Source: Seksarn et al. Asian

nigh risk, estimate that (234-102)=132 0-9 year olds were high risk

old high risk)	244
+ 35% of 0-9 years old)	234

0.35

)f the 0-9 year olds, 35% are assigned to high risk treatment, while 65% are

DIAGNOSED AND NOT DIAGNOSED

Age group	1	2	3
	**0-4**	**5-9**	**10-14**
**tandard risk**	159	113	0
**High risk**	86	61	113

	1	2	3
	**0-4**	**5-9**	**10-14**
**tandard risk**	144	108	0
**High risk**	78	59	108

		Scenario	0
	Proportion relapse	Basecase (=0	0.22
**en with ALL relapse**	0.22	Sensitivity an	0.5

For peer review only

gh risk protocol according to the risk allocation table for relapse (Thai ALL pro

3. Allocate different sites of relapse to either standard or high ris
Variables for risk allocation (relapse)
a) Immunophenotype: use proportions from the literature (these refer t
b) Site of relapse: use data from the literature to calculate proportions
c) Time of relapse: Assume equal distribution across time of relapse (1/
d) Intermediate risk: Assume equal allocation to SR and HR protocol (1/
Protocol: assume that among intermediate risk children, 1/2 receive SR

Immunophenotyion	immunophe	Site of relapse	rtion site of re	Very early<br>months from 1s
		Isolated extra	0.3	High
Non T-cell	0.87	Isolated marr	0.5	High
		Combined	0.2	High
		Isolated extra	0.3	High
T-cell	0.13	Isolated marr	0.5	High
		Combined	0.2	High

Source risk allocation table: p.55 Thai protocols

Proportion SR r
Proportion HR r

DIAGNOSED AND NOT DIAGNOSED

Age group	1<br>**0-4**	2<br>**5-9**	3<br>**10-14**
	53	38	25
	15	11	7
	38	27	18

DIAGNOSED AND NOT DIAGNOSED

Age group	1<br>**0-4**	2<br>**5-9**	3<br>**10-14**
	48	36	23
	13	10	7
	35	26	17

p for the baseline treatment

11.4609

For peer review only

/article/view/203011
formation_detail.aspx?ContentID=NjAwMDAwMjY5

For peer review only

nd 97.5 percentiles)

10-15	Total
41	197
64	296

10-15	Total
40	183
59	274

Upper bound of 95% uncertainty interval (calculated as the 2.!

2017	<1	1-4	5-9
Diagnosed	20	178	142
Diagnosed an	35	332	261

2021	<1	1-4	5-9
Diagnosed	19	166	138
Diagnosed an	31	306	251

Pac J Cancer Prev 2015

For peer review only

otocols 2016)

sk relapse treatment protocol

:o first DX), assumption: the same proportion applied to relapse

'3 each)
/2 each)
. and 1/2 HR protocol

ɔortion very e	Early	'roportion earl	Late	Proportion late
0.33		0.33		0.33
st aDXer 1st DX	and <6 months from stop		≥6 months after stopping therapy	
0.09	Intermediate	0.09	Standard	0.09
0.15	High	0.15	Intermediate	0.15
0.05	Intermediate	0.05	Intermediate	0.05
0.01	Intermediate	0.01	Standard	0.01
0.02	High	0.02	High	0.02
0.01	High	0.01	High	0.01

0.28	
0.72	

Scenario		0
Basecase (=0)		
	SR	0.28
	HR	0.72
Sensitivity analysis (=1)		
	SR	0.5
	HR	0.5

For peer review only

For peer review only

5 and 97.5 percentiles)

10-15	Total
94	434
167	795

10-15	Total
91	414
161	749

For peer review only

	Age group (years)	Age group (#)	Risk group description	Risk group (#)	Treatment phase
pedi_ALL	0-4	1	Standard risk	1	Induction
pedi_ALL	0-4	1	Standard risk	1	Induction
pedi_ALL	0-4	1	Standard risk	1	Induction
pedi_ALL	0-4	1	Standard risk	1	Induction
pedi_ALL	0-4	1	Standard risk	1	Consolidation
pedi_ALL	0-4	1	Standard risk	1	Consolidation
pedi_ALL	0-4	1	Standard risk	1	Consolidation
pedi_ALL	0-4	1	Standard risk	1	Interim Maintenance
pedi_ALL	0-4	1	Standard risk	1	Interim Maintenance
pedi_ALL	0-4	1	Standard risk	1	Interim Maintenance
pedi_ALL	0-4	1	Standard risk	1	Interim Maintenance
pedi_ALL	0-4	1	Standard risk	1	Delayed Intensification
pedi_ALL	0-4	1	Standard risk	1	Delayed Intensification
pedi_ALL	0-4	1	Standard risk	1	Delayed Intensification
pedi_ALL	0-4	1	Standard risk	1	Delayed Intensification
pedi_ALL	0-4	1	Standard risk	1	Delayed Intensification
pedi_ALL	0-4	1	Standard risk	1	Delayed Intensification
pedi_ALL	0-4	1	Standard risk	1	Delayed Intensification
pedi_ALL	0-4	1	Standard risk	1	Delayed Intensification
pedi_ALL	0-4	1	Standard risk	1	Maintenance
pedi_ALL	0-4	1	Standard risk	1	Maintenance
pedi_ALL	0-4	1	Standard risk	1	Maintenance
pedi_ALL	0-4	1	Standard risk	1	Maintenance
pedi_ALL	0-4	1	Standard risk	1	Maintenance
pedi_ALL	0-4	1	High risk	2	Induction
pedi_ALL	0-4	1	High risk	2	Induction
pedi_ALL	0-4	1	High risk	2	Induction
pedi_ALL	0-4	1	High risk	2	Induction
pedi_ALL	0-4	1	High risk	2	Induction
pedi_ALL	0-4	1	High risk	2	Consolidation
pedi_ALL	0-4	1	High risk	2	Consolidation
pedi_ALL	0-4	1	High risk	2	Consolidation
pedi_ALL	0-4	1	High risk	2	Consolidation
pedi_ALL	0-4	1	High risk	2	Consolidation
pedi_ALL	0-4	1	High risk	2	Consolidation
pedi_ALL	0-4	1	High risk	2	Consolidation
pedi_ALL	0-4	1	High risk	2	Interim Maintenance
pedi_ALL	0-4	1	High risk	2	Interim Maintenance
pedi_ALL	0-4	1	High risk	2	Interim Maintenance
pedi_ALL	0-4	1	High risk	2	Interim Maintenance
pedi_ALL	0-4	1	High risk	2	Interim Maintenance
pedi_ALL	0-4	1	High risk	2	Delayed Intensification
pedi_ALL	0-4	1	High risk	2	Delayed Intensification
pedi_ALL	0-4	1	High risk	2	Delayed Intensification
pedi_ALL	0-4	1	High risk	2	Delayed Intensification
pedi_ALL	0-4	1	High risk	2	Delayed Intensification
pedi_ALL	0-4	1	High risk	2	Delayed Intensification
pedi_ALL	0-4	1	High risk	2	Delayed Intensification
pedi_ALL	0-4	1	High risk	2	Delayed Intensification

pedi_ALL	0-4	1	High risk	2	Delayed Intensification
pedi_ALL	0-4	1	High risk	2	Maintenance
pedi_ALL	0-4	1	High risk	2	Maintenance
pedi_ALL	0-4	1	High risk	2	Maintenance
pedi_ALL	0-4	1	High risk	2	Maintenance
pedi_ALL	0-4	1	High risk	2	Maintenance
pedi_ALL	0-4	1	Relapse	3	Phase 1
pedi_ALL	0-4	1	Relapse	3	Phase 1
pedi_ALL	0-4	1	Relapse	3	Phase 1
pedi_ALL	0-4	1	Relapse	3	Phase 1
pedi_ALL	0-4	1	Relapse	3	Phase 1
pedi_ALL	0-4	1	Relapse	3	Phase 2
pedi_ALL	0-4	1	Relapse	3	Phase 2
pedi_ALL	0-4	1	Relapse	3	Phase 2
pedi_ALL	0-4	1	Relapse	3	Phase 2
pedi_ALL	0-4	1	Relapse	3	Phase 2
pedi_ALL	0-4	1	Relapse	3	Phase 2
pedi_ALL	0-4	1	Relapse	3	Phase 2
pedi_ALL	0-4	1	Relapse	3	Phase 2
pedi_ALL	0-4	1	Relapse	3	Phase 3
pedi_ALL	0-4	1	Relapse	3	Phase 3
pedi_ALL	0-4	1	Relapse	3	Phase 3
pedi_ALL	0-4	1	Relapse	3	Phase 3
pedi_ALL	0-4	1	Relapse	3	Phase 3
pedi_ALL	0-4	1	Relapse	3	Phase 3
pedi_ALL	0-4	1	Relapse	3	Phase 3
pedi_ALL	0-4	1	Relapse	4	Phase 1
pedi_ALL	0-4	1	Relapse	4	Phase 1
pedi_ALL	0-4	1	Relapse	4	Phase 1
pedi_ALL	0-4	1	Relapse	4	Phase 1
pedi_ALL	0-4	1	Relapse	4	Phase 1
pedi_ALL	0-4	1	Relapse	4	Phase 2
pedi_ALL	0-4	1	Relapse	4	Phase 2
pedi_ALL	0-4	1	Relapse	4	Phase 2
pedi_ALL	0-4	1	Relapse	4	Phase 2
pedi_ALL	0-4	1	Relapse	4	Phase 2
pedi_ALL	0-4	1	Relapse	4	Phase 2
pedi_ALL	0-4	1	Relapse	4	Phase 2
pedi_ALL	0-4	1	Relapse	4	Phase 2
pedi_ALL	0-4	1	Relapse	4	Phase 3
pedi_ALL	0-4	1	Relapse	4	Phase 3
pedi_ALL	0-4	1	Relapse	4	Phase 3
pedi_ALL	0-4	1	Relapse	4	Phase 3
pedi_ALL	0-4	1	Relapse	4	Phase 3
pedi_ALL	0-4	1	Relapse	4	Phase 3
pedi_ALL	0-4	1	Relapse	4	Phase 3
pedi_ALL	0-4	1	Relapse	4	Phase 4
pedi_ALL	0-4	1	Relapse	3	Phase 5
pedi_ALL	0-4	1	Relapse	3	Phase 5
pedi_ALL	0-4	1	Relapse	3	Phase 5
pedi_ALL	0-4	1	Relapse	3	Phase 5
pedi_ALL	0-4	1	Relapse	3	Phase 5
pedi_ALL	0-4	1	Relapse	3	Phase 5
pedi_ALL	0-4	1	Relapse	3	Phase 5
pedi_ALL	0-4	1	Relapse	3	Phase 5
pedi_ALL	0-4	1	Relapse	3	Phase 5

For peer review only

pedi_ALL	0-4	1	Relapse	3	Phase 6
pedi_ALL	0-4	1	Relapse	3	Phase 6
pedi_ALL	0-4	1	Relapse	3	Phase 6
pedi_ALL	0-4	1	Relapse	3	Phase 6
pedi_ALL	0-4	1	Relapse	3	Phase 6
pedi_ALL	5-9	2	Standard risk	1	Induction
pedi_ALL	5-9	2	Standard risk	1	Induction
pedi_ALL	5-9	2	Standard risk	1	Induction
pedi_ALL	5-9	2	Standard risk	1	Induction
pedi_ALL	5-9	2	Standard risk	1	Consolidation
pedi_ALL	5-9	2	Standard risk	1	Consolidation
pedi_ALL	5-9	2	Standard risk	1	Consolidation
pedi_ALL	5-9	2	Standard risk	1	Interim Maintenance
pedi_ALL	5-9	2	Standard risk	1	Interim Maintenance
pedi_ALL	5-9	2	Standard risk	1	Interim Maintenance
pedi_ALL	5-9	2	Standard risk	1	Interim Maintenance
pedi_ALL	5-9	2	Standard risk	1	Delayed Intensification
pedi_ALL	5-9	2	Standard risk	1	Delayed Intensification
pedi_ALL	5-9	2	Standard risk	1	Delayed Intensification
pedi_ALL	5-9	2	Standard risk	1	Delayed Intensification
pedi_ALL	5-9	2	Standard risk	1	Delayed Intensification
pedi_ALL	5-9	2	Standard risk	1	Delayed Intensification
pedi_ALL	5-9	2	Standard risk	1	Delayed Intensification
pedi_ALL	5-9	2	Standard risk	1	Delayed Intensification
pedi_ALL	5-9	2	Standard risk	1	Maintenance
pedi_ALL	5-9	2	Standard risk	1	Maintenance
pedi_ALL	5-9	2	Standard risk	1	Maintenance
pedi_ALL	5-9	2	Standard risk	1	Maintenance
pedi_ALL	5-9	2	Standard risk	1	Maintenance
pedi_ALL	5-9	2	High risk	2	Induction
pedi_ALL	5-9	2	High risk	2	Induction
pedi_ALL	5-9	2	High risk	2	Induction
pedi_ALL	5-9	2	High risk	2	Induction
pedi_ALL	5-9	2	High risk	2	Induction
pedi_ALL	5-9	2	High risk	2	Consolidation
pedi_ALL	5-9	2	High risk	2	Consolidation
pedi_ALL	5-9	2	High risk	2	Consolidation
pedi_ALL	5-9	2	High risk	2	Consolidation
pedi_ALL	5-9	2	High risk	2	Consolidation
pedi_ALL	5-9	2	High risk	2	Consolidation
pedi_ALL	5-9	2	High risk	2	Consolidation
pedi_ALL	5-9	2	High risk	2	Interim Maintenance
pedi_ALL	5-9	2	High risk	2	Interim Maintenance
pedi_ALL	5-9	2	High risk	2	Interim Maintenance
pedi_ALL	5-9	2	High risk	2	Interim Maintenance
pedi_ALL	5-9	2	High risk	2	Interim Maintenance
pedi_ALL	5-9	2	High risk	2	Delayed Intensification
pedi_ALL	5-9	2	High risk	2	Delayed Intensification
pedi_ALL	5-9	2	High risk	2	Delayed Intensification
pedi_ALL	5-9	2	High risk	2	Delayed Intensification
pedi_ALL	5-9	2	High risk	2	Delayed Intensification
pedi_ALL	5-9	2	High risk	2	Delayed Intensification
pedi_ALL	5-9	2	High risk	2	Delayed Intensification
pedi_ALL	5-9	2	High risk	2	Delayed Intensification
pedi_ALL	5-9	2	High risk	2	Delayed Intensification
pedi_ALL	5-9	2	High risk	2	Maintenance

pedi_ALL	5-9	2	High risk	2	Maintenance
pedi_ALL	5-9	2	High risk	2	Maintenance
pedi_ALL	5-9	2	High risk	2	Maintenance
pedi_ALL	5-9	2	High risk	2	Maintenance
pedi_ALL	5-9	2	Relapse	3	Phase 1
pedi_ALL	5-9	2	Relapse	3	Phase 1
pedi_ALL	5-9	2	Relapse	3	Phase 1
pedi_ALL	5-9	2	Relapse	3	Phase 1
pedi_ALL	5-9	2	Relapse	3	Phase 1
pedi_ALL	5-9	2	Relapse	3	Phase 2
pedi_ALL	5-9	2	Relapse	3	Phase 2
pedi_ALL	5-9	2	Relapse	3	Phase 2
pedi_ALL	5-9	2	Relapse	3	Phase 2
pedi_ALL	5-9	2	Relapse	3	Phase 2
pedi_ALL	5-9	2	Relapse	3	Phase 2
pedi_ALL	5-9	2	Relapse	3	Phase 2
pedi_ALL	5-9	2	Relapse	3	Phase 2
pedi_ALL	5-9	2	Relapse	3	Phase 3
pedi_ALL	5-9	2	Relapse	3	Phase 3
pedi_ALL	5-9	2	Relapse	3	Phase 3
pedi_ALL	5-9	2	Relapse	3	Phase 3
pedi_ALL	5-9	2	Relapse	3	Phase 3
pedi_ALL	5-9	2	Relapse	3	Phase 3
pedi_ALL	5-9	2	Relapse	3	Phase 3
pedi_ALL	5-9	2	Relapse	4	Phase 1
pedi_ALL	5-9	2	Relapse	4	Phase 1
pedi_ALL	5-9	2	Relapse	4	Phase 1
pedi_ALL	5-9	2	Relapse	4	Phase 1
pedi_ALL	5-9	2	Relapse	4	Phase 1
pedi_ALL	5-9	2	Relapse	4	Phase 2
pedi_ALL	5-9	2	Relapse	4	Phase 2
pedi_ALL	5-9	2	Relapse	4	Phase 2
pedi_ALL	5-9	2	Relapse	4	Phase 2
pedi_ALL	5-9	2	Relapse	4	Phase 2
pedi_ALL	5-9	2	Relapse	4	Phase 2
pedi_ALL	5-9	2	Relapse	4	Phase 2
pedi_ALL	5-9	2	Relapse	4	Phase 2
pedi_ALL	5-9	2	Relapse	4	Phase 3
pedi_ALL	5-9	2	Relapse	4	Phase 3
pedi_ALL	5-9	2	Relapse	4	Phase 3
pedi_ALL	5-9	2	Relapse	4	Phase 3
pedi_ALL	5-9	2	Relapse	4	Phase 3
pedi_ALL	5-9	2	Relapse	4	Phase 3
pedi_ALL	5-9	2	Relapse	4	Phase 3
pedi_ALL	5-9	2	Relapse	4	Phase 4
pedi_ALL	5-9	2	Relapse	3	Phase 5
pedi_ALL	5-9	2	Relapse	3	Phase 5
pedi_ALL	5-9	2	Relapse	3	Phase 5
pedi_ALL	5-9	2	Relapse	3	Phase 5
pedi_ALL	5-9	2	Relapse	3	Phase 5
pedi_ALL	5-9	2	Relapse	3	Phase 5
pedi_ALL	5-9	2	Relapse	3	Phase 5
pedi_ALL	5-9	2	Relapse	3	Phase 5
pedi_ALL	5-9	2	Relapse	3	Phase 5
pedi_ALL	5-9	2	Relapse	3	Phase 6
pedi_ALL	5-9	2	Relapse	3	Phase 6

For peer review only

pedi_ALL	5-9	2	Relapse	3	Phase 6
pedi_ALL	5-9	2	Relapse	3	Phase 6
pedi_ALL	5-9	2	Relapse	3	Phase 6
pedi_ALL	10-14	3	High risk	2	Induction
pedi_ALL	10-14	3	High risk	2	Induction
pedi_ALL	10-14	3	High risk	2	Induction
pedi_ALL	10-14	3	High risk	2	Induction
pedi_ALL	10-14	3	High risk	2	Induction
pedi_ALL	10-14	3	High risk	2	Consolidation
pedi_ALL	10-14	3	High risk	2	Consolidation
pedi_ALL	10-14	3	High risk	2	Consolidation
pedi_ALL	10-14	3	High risk	2	Consolidation
pedi_ALL	10-14	3	High risk	2	Consolidation
pedi_ALL	10-14	3	High risk	2	Consolidation
pedi_ALL	10-14	3	High risk	2	Consolidation
pedi_ALL	10-14	3	High risk	2	Interim Maintenance
pedi_ALL	10-14	3	High risk	2	Interim Maintenance
pedi_ALL	10-14	3	High risk	2	Interim Maintenance
pedi_ALL	10-14	3	High risk	2	Interim Maintenance
pedi_ALL	10-14	3	High risk	2	Interim Maintenance
pedi_ALL	10-14	3	High risk	2	Delayed Intensification
pedi_ALL	10-14	3	High risk	2	Delayed Intensification
pedi_ALL	10-14	3	High risk	2	Delayed Intensification
pedi_ALL	10-14	3	High risk	2	Delayed Intensification
pedi_ALL	10-14	3	High risk	2	Delayed Intensification
pedi_ALL	10-14	3	High risk	2	Delayed Intensification
pedi_ALL	10-14	3	High risk	2	Delayed Intensification
pedi_ALL	10-14	3	High risk	2	Delayed Intensification
pedi_ALL	10-14	3	High risk	2	Delayed Intensification
pedi_ALL	10-14	3	High risk	2	Maintenance
pedi_ALL	10-14	3	High risk	2	Maintenance
pedi_ALL	10-14	3	High risk	2	Maintenance
pedi_ALL	10-14	3	High risk	2	Maintenance
pedi_ALL	10-14	3	High risk	2	Maintenance
pedi_ALL	10-14	3	Relapse	3	Phase 1
pedi_ALL	10-14	3	Relapse	3	Phase 1
pedi_ALL	10-14	3	Relapse	3	Phase 1
pedi_ALL	10-14	3	Relapse	3	Phase 1
pedi_ALL	10-14	3	Relapse	3	Phase 1
pedi_ALL	10-14	3	Relapse	3	Phase 2
pedi_ALL	10-14	3	Relapse	3	Phase 2
pedi_ALL	10-14	3	Relapse	3	Phase 2
pedi_ALL	10-14	3	Relapse	3	Phase 2
pedi_ALL	10-14	3	Relapse	3	Phase 2
pedi_ALL	10-14	3	Relapse	3	Phase 2
pedi_ALL	10-14	3	Relapse	3	Phase 2
pedi_ALL	10-14	3	Relapse	3	Phase 2
pedi_ALL	10-14	3	Relapse	3	Phase 3
pedi_ALL	10-14	3	Relapse	3	Phase 3
pedi_ALL	10-14	3	Relapse	3	Phase 3
pedi_ALL	10-14	3	Relapse	3	Phase 3
pedi_ALL	10-14	3	Relapse	3	Phase 3
pedi_ALL	10-14	3	Relapse	3	Phase 3
pedi_ALL	10-14	3	Relapse	3	Phase 3
pedi_ALL	10-14	3	Relapse	4	Phase 1
pedi_ALL	10-14	3	Relapse	4	Phase 1

For peer review only

pedi_ALL	10-14	3	Relapse	4	Phase 1
pedi_ALL	10-14	3	Relapse	4	Phase 1
pedi_ALL	10-14	3	Relapse	4	Phase 1
pedi_ALL	10-14	3	Relapse	4	Phase 2
pedi_ALL	10-14	3	Relapse	4	Phase 2
pedi_ALL	10-14	3	Relapse	4	Phase 2
pedi_ALL	10-14	3	Relapse	4	Phase 2
pedi_ALL	10-14	3	Relapse	4	Phase 2
pedi_ALL	10-14	3	Relapse	4	Phase 2
pedi_ALL	10-14	3	Relapse	4	Phase 2
pedi_ALL	10-14	3	Relapse	4	Phase 2
pedi_ALL	10-14	3	Relapse	4	Phase 3
pedi_ALL	10-14	3	Relapse	4	Phase 3
pedi_ALL	10-14	3	Relapse	4	Phase 3
pedi_ALL	10-14	3	Relapse	4	Phase 3
pedi_ALL	10-14	3	Relapse	4	Phase 3
pedi_ALL	10-14	3	Relapse	4	Phase 3
pedi_ALL	10-14	3	Relapse	4	Phase 3
pedi_ALL	10-14	3	Relapse	4	Phase 4
pedi_ALL	10-14	3	Relapse	3	Phase 5
pedi_ALL	10-14	3	Relapse	3	Phase 5
pedi_ALL	10-14	3	Relapse	3	Phase 5
pedi_ALL	10-14	3	Relapse	3	Phase 5
pedi_ALL	10-14	3	Relapse	3	Phase 5
pedi_ALL	10-14	3	Relapse	3	Phase 5
pedi_ALL	10-14	3	Relapse	3	Phase 5
pedi_ALL	10-14	3	Relapse	3	Phase 5
pedi_ALL	10-14	3	Relapse	3	Phase 5
pedi_ALL	10-14	3	Relapse	3	Phase 6
pedi_ALL	10-14	3	Relapse	3	Phase 6
pedi_ALL	10-14	3	Relapse	3	Phase 6
pedi_ALL	10-14	3	Relapse	3	Phase 6
pedi_ALL	10-14	3	Relapse	3	Phase 6

For peer review only

Treatment phase (#)	smallest_unit	INN
0	1	Vincristine
0	10,000	Asparaginase
0	5	Prednisolone
0	11	Methotrexate
1	1	Vincristine
1	50	Mercaptopurine
1	11	Methotrexate
2	1	Vincristine
2	50	Methotrexate
2	30	Leucovorin
2	11	Methotrexate
3	1	Vincristine
3	10	Doxorubicin
3	2	Dexamethasone
3	500	Cyclophosphamide
3	40	Tioguanine
3	10,000	Asparaginase
3	11	Methotrexate
3	100	Cytarabine
4	1	Vincristine
4	5	Prednisolone
4	50	Mercaptopurine
4	2.5	Methotrexate
4	11	Methotrexate
0	1	Vincristine
0	10	Doxorubicin
0	10,000	Asparaginase
0	5	Prednisolone
0	11	Methotrexate
1	1	Vincristine
1	50	Mercaptopurine
1	11	Methotrexate
1	100	Cytarabine
1	10,000	Asparaginase
1	400	MESNA
1	500	Cyclophosphamide
2	1	Vincristine
2	50	Methotrexate
2	11	Methotrexate
2	50	Mercaptopurine
2	30	Leucovorin
3	1	Vincristine
3	10	Doxorubicin
3	2	Dexamethasone
3	500	Cyclophosphamide
3	400	MESNA
3	40	Tioguanine
3	10,000	Asparaginase
3	11	Methotrexate

For peer review only

3	100	Cytarabine
4	1	Vincristine
4	5	Prednisolone
4	50	Mercaptopurine
4	11	Methotrexate
4	2.5	Methotrexate
5	1	Vincristine
5	10	Mitoxantrone
5	10,000	Asparaginase
5	11	Methotrexate
5	2	Dexamethasone
6	2	Dexamethasone
6	1	Vincristine
6	50	Methotrexate
6	30	Leucovorin
6	10,000	Asparaginase
6	500	Cyclophosphamide
6	100	Etoposide
6	11	Methotrexate
7	2	Dexamethasone
7	1	Vincristine
7	100	Cytarabine
7	10,000	Asparaginase
7	50	Methotrexate
7	30	Leucovorin
7	11	Methotrexate
5	1	Vincristine
5	10	Mitoxantrone
5	10,000	Asparaginase
5	11	Methotrexate
5	2	Dexamethasone
6	2	Dexamethasone
6	1	Vincristine
6	50	Methotrexate
6	30	Leucovorin
6	10,000	Asparaginase
6	500	Cyclophosphamide
6	100	Etoposide
6	11	Methotrexate
7	2	Dexamethasone
7	1	Vincristine
7	100	Cytarabine
7	10,000	Asparaginase
7	50	Methotrexate
7	30	Leucovorin
7	11	Methotrexate
8	100	Cytarabine
9	2	Dexamethasone
9	1	Vincristine
9	50	Mercaptopurine
9	2.5	Methotrexate
9	2.5	Methotrexate
9	500	Cyclophosphamide
9	100	Etoposide
9	100	Cytarabine
9	11	Methotrexate

For peer review only

10	2	Dexamethasone
10	1	Vincristine
10	50	Mercaptopurine
10	2.5	Methotrexate
10	11	Methotrexate
0	1	Vincristine
0	10,000	Asparaginase
0	5	Prednisolone
0	11	Methotrexate
1	1	Vincristine
1	50	Mercaptopurine
1	11	Methotrexate
2	1	Vincristine
2	50	Methotrexate
2	30	Leucovorin
2	11	Methotrexate
3	1	Vincristine
3	10	Doxorubicin
3	2	Dexamethasone
3	500	Cyclophosphamide
3	40	Tioguanine
3	10,000	Asparaginase
3	11	Methotrexate
3	100	Cytarabine
4	1	Vincristine
4	5	Prednisolone
4	50	Mercaptopurine
4	2.5	Methotrexate
4	11	Methotrexate
0	1	Vincristine
0	10	Doxorubicin
0	10,000	Asparaginase
0	5	Prednisolone
0	11	Methotrexate
1	1	Vincristine
1	50	Mercaptopurine
1	11	Methotrexate
1	100	Cytarabine
1	10,000	Asparaginase
1	400	MESNA
1	500	Cyclophosphamide
2	1	Vincristine
2	50	Methotrexate
2	11	Methotrexate
2	50	Mercaptopurine
2	30	Leucovorin
3	1	Vincristine
3	10	Doxorubicin
3	2	Dexamethasone
3	500	Cyclophosphamide
3	400	MESNA
3	40	Tioguanine
3	10,000	Asparaginase
3	11	Methotrexate
3	100	Cytarabine
4	1	Vincristine

For peer review only

4	5	Prednisolone
4	50	Mercaptopurine
4	11	Methotrexate
4	2.5	Methotrexate
5	1	Vincristine
5	10	Mitoxantrone
5	10,000	Asparaginase
5	11	Methotrexate
5	2	Dexamethasone
6	2	Dexamethasone
6	1	Vincristine
6	50	Methotrexate
6	30	Leucovorin
6	10,000	Asparaginase
6	500	Cyclophosphamide
6	100	Etoposide
6	11	Methotrexate
7	2	Dexamethasone
7	1	Vincristine
7	100	Cytarabine
7	10,000	Asparaginase
7	50	Methotrexate
7	30	Leucovorin
7	11	Methotrexate
5	1	Vincristine
5	10	Mitoxantrone
5	10,000	Asparaginase
5	11	Methotrexate
5	2	Dexamethasone
6	2	Dexamethasone
6	1	Vincristine
6	50	Methotrexate
6	30	Leucovorin
6	10,000	Asparaginase
6	500	Cyclophosphamide
6	100	Etoposide
6	11	Methotrexate
7	2	Dexamethasone
7	1	Vincristine
7	100	Cytarabine
7	10,000	Asparaginase
7	50	Methotrexate
7	30	Leucovorin
7	11	Methotrexate
8	100	Cytarabine
9	2	Dexamethasone
9	1	Vincristine
9	50	Mercaptopurine
9	2.5	Methotrexate
9	2.5	Methotrexate
9	500	Cyclophosphamide
9	100	Etoposide
9	100	Cytarabine
9	11	Methotrexate
10	2	Dexamethasone
10	1	Vincristine

For peer review only

10	50	Mercaptopurine
10	2.5	Methotrexate
10	11	Methotrexate
0	1	Vincristine
0	10	Doxorubicin
0	10,000	Asparaginase
0	5	Prednisolone
0	11	Methotrexate
1	1	Vincristine
1	50	Mercaptopurine
1	11	Methotrexate
1	100	Cytarabine
1	10,000	Asparaginase
1	400	MESNA
1	500	Cyclophosphamide
2	1	Vincristine
2	50	Methotrexate
2	11	Methotrexate
2	50	Mercaptopurine
2	30	Leucovorin
3	1	Vincristine
3	10	Doxorubicin
3	2	Dexamethasone
3	500	Cyclophosphamide
3	400	MESNA
3	40	Tioguanine
3	10,000	Asparaginase
3	11	Methotrexate
3	100	Cytarabine
4	1	Vincristine
4	5	Prednisolone
4	50	Mercaptopurine
4	11	Methotrexate
4	2.5	Methotrexate
5	1	Vincristine
5	10	Mitoxantrone
5	10,000	Asparaginase
5	11	Methotrexate
5	2	Dexamethasone
6	2	Dexamethasone
6	1	Vincristine
6	50	Methotrexate
6	30	Leucovorin
6	10,000	Asparaginase
6	500	Cyclophosphamide
6	100	Etoposide
6	11	Methotrexate
7	2	Dexamethasone
7	1	Vincristine
7	100	Cytarabine
7	10,000	Asparaginase
7	50	Methotrexate
7	30	Leucovorin
7	11	Methotrexate
5	1	Vincristine
5	10	Mitoxantrone

For peer review only

5	10,000	Asparaginase
5	11	Methotrexate
5	2	Dexamethasone
6	2	Dexamethasone
6	1	Vincristine
6	50	Methotrexate
6	30	Leucovorin
6	10,000	Asparaginase
6	500	Cyclophosphamide
6	100	Etoposide
6	11	Methotrexate
7	2	Dexamethasone
7	1	Vincristine
7	100	Cytarabine
7	10,000	Asparaginase
7	50	Methotrexate
7	30	Leucovorin
7	11	Methotrexate
8	100	Cytarabine
9	2	Dexamethasone
9	1	Vincristine
9	50	Mercaptopurine
9	2.5	Methotrexate
9	2.5	Methotrexate
9	500	Cyclophosphamide
9	100	Etoposide
9	100	Cytarabine
9	11	Methotrexate
10	2	Dexamethasone
10	1	Vincristine
10	50	Mercaptopurine
10	2.5	Methotrexate
10	11	Methotrexate

For peer review only

Dosage and formulation according to Thai guidelines	form	oral	IV	IM
1.5 mg/m2/dose IV push (Max 2 mg)	IV		1	
10,000 IU/m2/dose IM	IM			1
30 mg/m2/dose PO BID (No max dose)	tab	1		
	IV			
1.5 mg/m2/dose IV push (Max 2 mg)	IV		1	
75 mg/m2/dose PO daily (hs)	tab	1		
	IV			
1.5 mg/m2/dose IV push (Max 2 mg)	IV		1	
2.5 gm/m2/dose IV infusion in 24 hr (No max	IV		1	
15 mg/m2/dose PO/IV after each HD MTX infu	IV	1	1	
	IV			
1.5 mg/m2/dose IV push (Max 2 mg)	IV		1	
25 mg/m2/dose IV	IV		1	
5 mg/m2/dose PO BID	tab	1		
1000 mg/m2/dose IV	IV			
60 mg/m2/dose PO daily (hs)	tab	1		
10,000 IU/m2/dose IM	IM			1
	IV			
75 mg/m2/dose IV over 15-30 min or SC daily	IV		1	
1.5 mg/m2/dose IV push (Max 2 mg)	IV		1	
20 mg/m2/dose PO BID	tab	1		
50 mg/m2/dose PO daily (hs)	tab	1		
20 mg/m2/dose PO weekly	tab	1		
	IV			
1.5 mg/m2/dose IV push over 1 min (Max 2 m	IV		1	
25 mg/m2/dose IV push	IV		1	
10,000 IU/m2/dose IM	IM			1
30 mg/m2/dose PO BID (No max dose)	tab	1		
	IV			
1.5 mg/m2/dose IV push	IV		1	
60 mg/m2/dose PO daily (hs)	tab	1		
	IV			
75 mg/m2/dose IV or SC daily	IV		1	
25,000 IU/m2/dose IM	IM			1
250 mg/m2/dose IV drip in 15 minutes at hr 0	IV		1	
1,000 mg/m2/dose IV	IV		1	
1.5 mg/m2/dose IV push (Max 2 mg)	IV		1	
5 gm/m2/dose IV infusion (No max dose)	IV		1	
	IV			
25 mg/m2/dose PO daily (hs)	tab	1		
15 mg/m2/dose PO/IV	IV	1	1	
1.5 mg/m2/dose IV push (Max 2 mg)	IV		1	
25 mg/m2/dose IV	IV		1	
5 mg/m2/dose PO BID	tab	1		
1,000 mg/m2/dose IV	IV		1	
250 mg/m2/dose IV drip	IV		1	
60 mg/m2/dose PO daily (hs)	tab	1		
10,000 IU/m2/dose IM	IM			1
	IV			

For peer review only

75 mg/m2/dose IV or SC daily	IV		1	
1.5 mg/m2/dose IV push (Max 2 mg)	IV		1	
20 mg/m2/dose PO BID	tab	1		
50 mg/m2/dose PO daily (hs)	tab	1		
	IV			
20 mg/m2/dose PO	tab	1		
1.5 mg/m2 IV push (max 2 mg)	IV		1	
10 mg/m2 IV	IV		1	
10,000 unit/m2 IM	IM			1
	IV			
20 mg/m2/day PO/IV	tab	1	1	
6 mg/m2/day PO BID	tab	1		
1.5 mg/m2 IV push (max 2 mg)	IV		1	
1,000 mg/m2 IV drip	IV		1	
15 mg/m2 IV	IV		1	
10,000 unit/m2 IM	IM			1
440 mg/m2 IV drip	IV		1	
100 mg/m2 IV drip	IV		1	
	IV			
6 mg/m2/day PO BID	tab	1		
1.5 mg/m2IV push (max 2 mg)	IV		1	
3,000 mg IV drip	IV		1	
10,000 unit/m2 IM	IM			1
1,000 mg/m2 IV drip	IV		1	
15 mg/m2 IV	IV		1	
	IV			
1.5 mg/m2 IV push (max 2 mg)	IV		1	
10 mg/m2 IV	IV		1	
10,000 unit/m2 IM	IM			1
	IV			
20 mg/m2/day PO/IV	tab	1	1	
6 mg/m2/day PO BID	tab	1		
1.5 mg/m2 IV push (max 2 mg)	IV		1	
1,000 mg/m2 IV drip	IV		1	
15 mg/m2 IV	IV		1	
10,000 unit/m2 IM	IM			1
440 mg/m2 IV drip	IV		1	
100 mg/m2 IV drip	IV		1	
	IV			
6 mg/m2/day PO BID	tab	1		
1.5 mg/m2IV push (max 2 mg)	IV		1	
3,000 mg IV drip	IV		1	
10,000 unit/m2 IM	IM			1
1,000 mg/m2 IV drip	IV		1	
15 mg/m2 IV	IV		1	
	IV			
1,000 mg/m2 IV drip	IV		1	
6 mg/m2/day PO BID	tab	1		
1.5 mg /m2 IV push (max 2 mg)	IV		1	
75 mg/m2 PO hs	tab	1		
20 mg/m2 PO hs	tab	1		
25 mg/m2/dose PO	tab	1		
300 mg/m2 IV drip	IV		1	
150 mg/m2 IV drip	IV		1	
50 mg/m2 IV/SC	IV		1	
	IV			

For peer review only

6 mg/m2/day PO BID	tab	1		
1.5 mg/m2 IV push (max 2 mg)	IV		1	
75 mg/m2 PO	tab	1		
20 mg/m2 PO	tab	1		
	IV			
1.5 mg/m2/dose IV push (Max 2 mg)	IV		1	
10,000 IU/m2/dose IM	IM			1
30 mg/m2/dose PO BID (No max dose)	tab	1		
	IV			
1.5 mg/m2/dose IV push (Max 2 mg)	IV		1	
75 mg/m2/dose PO daily (hs)	tab	1		
	IV			
1.5 mg/m2/dose IV push (Max 2 mg)	IV		1	
2.5 gm/m2/dose IV infusion in 24 hr (No max	IV		1	
15 mg/m2/dose PO/IV after each HD MTX infu	IV	1	1	
	IV			
1.5 mg/m2/dose IV push (Max 2 mg)	IV		1	
25 mg/m2/dose IV	IV		1	
5 mg/m2/dose PO BID	tab	1		
1000 mg/m2/dose IV	IV			
60 mg/m2/dose PO daily (hs)	tab	1		
10,000 IU/m2/dose IM	IM			1
	IV			
75 mg/m2/dose IV over 15-30 min or SC daily	IV		1	
1.5 mg/m2/dose IV push (Max 2 mg)	IV		1	
20 mg/m2/dose PO BID	tab	1		
50 mg/m2/dose PO daily (hs)	tab	1		
20 mg/m2/dose PO weekly	tab	1		
	IV			
1.5 mg/m2/dose IV push over 1 min (Max 2 m	IV		1	
25 mg/m2/dose IV push	IV		1	
10,000 IU/m2/dose IM	IM			1
30 mg/m2/dose PO BID (No max dose)	tab	1		
	IV			
1.5 mg/m2/dose IV push	IV		1	
60 mg/m2/dose PO daily (hs)	tab	1		
	IV			
75 mg/m2/dose IV or SC daily	IV		1	
25,000 IU/m2/dose IM	IM			1
250 mg/m2/dose IV drip in 15 minutes at hr 0	IV		1	
1,000 mg/m2/dose IV	IV		1	
1.5 mg/m2/dose IV push (Max 2 mg)	IV		1	
5 gm/m2/dose IV infusion (No max dose)	IV		1	
	IV			
25 mg/m2/dose PO daily (hs)	tab	1		
15 mg/m2/dose PO/IV	IV			
1.5 mg/m2/dose IV push (Max 2 mg)	IV		1	
25 mg/m2/dose IV	IV		1	
5 mg/m2/dose PO BID	tab	1		
1,000 mg/m2/dose IV	IV		1	
250 mg/m2/dose IV drip	IV		1	
60 mg/m2/dose PO daily (hs)	tab	1		
10,000 IU/m2/dose IM	IM			1
	IV			
75 mg/m2/dose IV or SC daily	IV		1	
1.5 mg/m2/dose IV push (Max 2 mg)	IV		1	

For peer review only

20 mg/m2/dose PO BID	tab	1		
50 mg/m2/dose PO daily (hs)	tab	1		
	IV			
20 mg/m2/dose PO	tab	1		
1.5 mg/m2 IV push (max 2 mg)	IV		1	
10 mg/m2 IV	IV		1	
10,000 unit/m2 IM	IM			1
	IV			
20 mg/m2/day PO/IV	tab	1	1	
6 mg/m2/day PO BID	tab	1		
1.5 mg/m2 IV push (max 2 mg)	IV		1	
1,000 mg/m2 IV drip	IV		1	
15 mg/m2 IV	IV		1	
10,000 unit/m2 IM	IM			1
440 mg/m2 IV drip	IV		1	
100 mg/m2 IV drip	IV		1	
	IV			
6 mg/m2/day PO BID	tab	1		
1.5 mg/m2IV push (max 2 mg)	IV		1	
3,000 mg IV drip	IV		1	
10,000 unit/m2 IM	IM			1
1,000 mg/m2 IV drip	IV		1	
15 mg/m2 IV	IV		1	
	IV			
1.5 mg/m2 IV push (max 2 mg)	IV		1	
10 mg/m2 IV	IV		1	
10,000 unit/m2 IM	IM			1
	IV			
20 mg/m2/day PO/IV	tab	1	1	
6 mg/m2/day PO BID	tab	1		
1.5 mg/m2 IV push (max 2 mg)	IV		1	
1,000 mg/m2 IV drip	IV		1	
15 mg/m2 IV	IV		1	
10,000 unit/m2 IM	IM			1
440 mg/m2 IV drip	IV		1	
100 mg/m2 IV drip	IV		1	
	IV			
6 mg/m2/day PO BID	tab	1		
1.5 mg/m2IV push (max 2 mg)	IV		1	
3,000 mg IV drip	IV		1	
10,000 unit/m2 IM	IM			1
1,000 mg/m2 IV drip	IV		1	
15 mg/m2 IV	IV		1	
	IV			
1,000 mg/m2 IV drip	IV		1	
6 mg/m2/day PO BID	tab	1		
1.5 mg /m2 IV push (max 2 mg)	IV		1	
75 mg/m2 PO hs	tab	1		
20 mg/m2 PO hs	tab	1		
25 mg/m2/dose PO	tab	1		
300 mg/m2 IV drip	IV		1	
150 mg/m2 IV drip	IV		1	
50 mg/m2 IV/SC	IV		1	
	IV			
6 mg/m2/day PO BID	tab	1		
1.5 mg/m2 IV push (max 2 mg)	IV		1	

For peer review only

75 mg/m2 PO	tab	1		
20 mg/m2 PO	tab	1		
	IV			
1.5 mg/m2/dose IV push over 1 min (Max 2 m	IV		1	
25 mg/m2/dose IV push	IV		1	
10,000 IU/m2/dose IM	IM			1
30 mg/m2/dose PO BID (No max dose)	tab	1		
	IV			
1.5 mg/m2/dose IV push	IV		1	
60 mg/m2/dose PO daily (hs)	tab	1		
	IV			
75 mg/m2/dose IV or SC daily	IV		1	
25,000 IU/m2/dose IM	IM			1
250 mg/m2/dose IV drip in 15 minutes at hr 0	IV		1	
1,000 mg/m2/dose IV	IV		1	
1.5 mg/m2/dose IV push (Max 2 mg)	IV		1	
5 gm/m2/dose IV infusion (No max dose)	IV		1	
	IV			
25 mg/m2/dose PO daily (hs)	tab	1		
15 mg/m2/dose PO/IV	IV			
1.5 mg/m2/dose IV push (Max 2 mg)	IV		1	
25 mg/m2/dose IV	IV		1	
5 mg/m2/dose PO BID	tab	1		
1,000 mg/m2/dose IV	IV		1	
250 mg/m2/dose IV drip	IV		1	
60 mg/m2/dose PO daily (hs)	tab	1		
10,000 IU/m2/dose IM	IM			1
	IV			
75 mg/m2/dose IV or SC daily	IV		1	
1.5 mg/m2/dose IV push (Max 2 mg)	IV		1	
20 mg/m2/dose PO BID	tab	1		
50 mg/m2/dose PO daily (hs)	tab	1		
	IV			
20 mg/m2/dose PO	tab	1		
1.5 mg/m2 IV push (max 2 mg)	IV		1	
10 mg/m2 IV	IV		1	
10,000 unit/m2 IM	IM			1
	IV			
20 mg/m2/day PO/IV	tab	1	1	
6 mg/m2/day PO BID	tab	1		
1.5 mg/m2 IV push (max 2 mg)	IV		1	
1,000 mg/m2 IV drip	IV		1	
15 mg/m2 IV	IV		1	
10,000 unit/m2 IM	IM			1
440 mg/m2 IV drip	IV		1	
100 mg/m2 IV drip	IV		1	
	IV			
6 mg/m2/day PO BID	tab	1		
1.5 mg/m2IV push (max 2 mg)	IV		1	
3,000 mg IV drip	IV		1	
10,000 unit/m2 IM	IM			1
1,000 mg/m2 IV drip	IV		1	
15 mg/m2 IV	IV		1	
	IV			
1.5 mg/m2 IV push (max 2 mg)	IV		1	
10 mg/m2 IV	IV		1	

For peer review only

10,000 unit/m2 IM	IM			1
	IV			
20 mg/m2/day PO/IV	tab	1	1	
6 mg/m2/day PO BID	tab	1		
1.5 mg/m2 IV push (max 2 mg)	IV		1	
1,000 mg/m2 IV drip	IV		1	
15 mg/m2 IV	IV		1	
10,000 unit/m2 IM	IM			1
440 mg/m2 IV drip	IV		1	
100 mg/m2 IV drip	IV		1	
	IV			
6 mg/m2/day PO BID	tab	1		
1.5 mg/m2IV push (max 2 mg)	IV		1	
3,000 mg IV drip	IV		1	
10,000 unit/m2 IM	IM			1
1,000 mg/m2 IV drip	IV		1	
15 mg/m2 IV	IV		1	
	IV			
1,000 mg/m2 IV drip	IV		1	
6 mg/m2/day PO BID	tab	1		
1.5 mg /m2 IV push (max 2 mg)	IV		1	
75 mg/m2 PO hs	tab	1		
20 mg/m2 PO hs	tab	1		
25 mg/m2/dose PO	tab	1		
300 mg/m2 IV drip	IV		1	
150 mg/m2 IV drip	IV		1	
50 mg/m2 IV/SC	IV		1	
	IV			
6 mg/m2/day PO BID	tab	1		
1.5 mg/m2 IV push (max 2 mg)	IV		1	
75 mg/m2 PO	tab	1		
20 mg/m2 PO	tab	1		
	IV			

For peer review only

IT	SC	mg_dose	dose_unit	Average Amount (mg dose * BSA)/Person Dose (mg)	Number of Doses/Cycle	Average Amount/Cycle (mg)
		1.5	mg/m2	0.832	4	3.327
		10,000	IU/m2	5544.609	6	33267.655
		30	mg/m2	16.634	28	465.747
1		11	mg	10.500	3	31.500
		2	mg/m2	0.832	2	1.663
		75	mg/m2	41.585	28	1164.368
1		11	mg	10.500	2	21.000
		1.5	mg/m2	0.832	4	3.327
1		2,500	mg/m2	1386.152	4	5544.609
		15	mg/m2	8.317	24	199.606
1		11	mg	10.500	4	42.000
		1.5	mg/m2	0.832	3	2.495
		25	mg/m2	13.862	3	41.585
		5	mg/m2	2.772	14	38.812
		1,000	mg/m2	554.461	1	554.461
		60	mg/m2	33.268	14	465.747
		10,000	IU/m2	5544.609	2	11089.218
1		11	mg	10.500	2	21.000
		75	mg/m2	41.585	8	332.677
		1.5	mg/m2	0.832	3	2.495
		20	mg/m2	11.089	15	166.338
		50	mg/m2	27.723	84	2328.736
		20	mg/m2	11.089	12	133.071
1		11	mg	10.500	1	10.500
		1.5	mg/m2	0.832	4	3.327
		25	mg/m2	13.862	4	55.446
		10,000	IU/m2	5544.609	6	33267.655
		30	mg/m2	16.634	28	465.747
1		11	mg	10.500	3	31.500
		1.5	mg/m2	0.832	4	3.327
		60	mg/m2	33.268	28	931.494
1		11	mg	10.500	4	42.000
	1	75	mg/m2	41.585	16	665.353
		25,000	IU/m2	13861.523	4	55446.091
		250	mg/m2	138.615	6	831.691
		1,000	mg/m2	554.461	2	1108.922
		1.5	mg/m2	0.832	4	3.327
		5,000	gm/m2	2772.305	4	11089.218
1		11	mg	10.500	2	21.000
		25	mg/m2	13.862	56	776.245
		15	mg/m2	8.317	24	199.606
		1.5	mg/m2	0.832	5	4.158
		25	mg/m2	13.862	3	41.585
		5	mg/m2	2.772	14	38.812
		1,000	mg/m2	554.461	1	554.461
		250	mg/m2	138.615	3	415.846
		60	mg/m2	33.268	14	465.747
		10,000	IU/m2	5544.609	4	22178.436
1		11	mg	10.500	3	31.500

For peer review only

	1	75	mg/m2	41.585	8	332.677
		1.5	mg/m2	0.832	3	2.495
		20	mg/m2	11.089	15	166.338
		50	mg/m2	27.723	84	2328.736
1		11	mg	10.500	1	10.500
		20	mg/m2	11.089	12	133.071
		2	mg/m2	0.832	4	3.327
		10	mg/m2	5.545	2	11.089
		10,000	IU/m2	5544.609	6	33267.655
1		11	mg	10.500	2	21.000
		20	mg/m2	11.089	10	110.892
		6	mg/m2	3.327	5	16.634
		2	mg/m2	0.832	1	0.832
		1,000	mg/m2	554.461	1	554.461
		15	mg/m2	8.317	6	49.901
		10,000	IU/m2	5544.609	3	16633.827
		440	mg/m2	243.963	5	1219.814
		100	mg/m2	55.446	5	277.230
1		11	mg	10.500	1	10.500
		6	mg/m2	3.327	5	16.634
		2	mg/m2	0.832	1	0.832
		3,000	mg/m2	1663.383	8	13307.062
		10,000	IU/m2	5544.609	5	27723.046
		1,000	mg/m2	554.461	1	554.461
		15	mg/m2	8.317	6	49.901
1		11	mg	10.500	2	21.000
		2	mg/m2	0.832	4	3.327
		10	mg/m2	5.545	2	11.089
		10,000	IU/m2	5544.609	6	33267.655
1		11	mg	10.500	2	21.000
		20	mg/m2	11.089	10	110.892
		6	mg/m2	3.327	5	16.634
		2	mg/m2	0.832	1	0.832
		1,000	mg/m2	554.461	1	554.461
		15	mg/m2	8.317	6	49.901
		10,000	IU/m2	5544.609	3	16633.827
		440	mg/m2	243.963	5	1219.814
		100	mg/m2	55.446	5	277.230
1		11	mg	10.500	1	10.500
		6	mg/m2	3.327	5	16.634
		2	mg/m2	0.832	1	0.832
		3,000	mg/m2	1663.383	8	13307.062
		10,000	IU/m2	5544.609	5	27723.046
		1,000	mg/m2	554.461	1	554.461
		15	mg/m2	8.317	6	49.901
1		11	mg	10.500	2	21.000
		1,000	mg/m2	554.461	5	2772.305
		6	mg/m2	3.327	5	16.634
		1.5	mg/m2	0.832	1	0.832
		75	mg/m2	41.585	42	1746.552
		20	mg/m2	11.089	4	44.357
		25	mg/m2	13.862	4	55.446
		300	mg/m2	166.338	2	332.677
		150	mg/m2	83.169	2	166.338
	1	50	mg/m2	27.723	8	221.784
1		10.5	mg	10.500	2	21.000

For peer review only

		6	mg/m2	3.327	5	16.634
		1.5	mg/m2	0.832	1	0.832
		75	mg/m2	41.585	525	21831.898
		20	mg/m2	11.089	75	831.691
1		10.5	mg	10.500	6	63.000
		1.5	mg/m2	1.324	4	5.297
		10,000	IU/m2	8829.028	6	52974.166
		30	mg/m2	26.487	28	741.638
1		11	mg	10.500	3	31.500
		2	mg/m2	1.324	2	2.649
		75	mg/m2	66.218	28	1854.096
1		11	mg	10.500	2	21.000
		1.5	mg/m2	1.324	4	5.297
1		2,500	mg/m2	2207.257	4	8829.028
		15	mg/m2	13.244	24	317.845
1		11	mg	10.500	4	42.000
		1.5	mg/m2	1.324	3	3.973
		25	mg/m2	22.073	3	66.218
		5	mg/m2	4.415	14	61.803
		1,000	mg/m2	882.903	1	882.903
		60	mg/m2	52.974	14	741.638
		10,000	IU/m2	8829.028	2	17658.055
1		11	mg	10.500	2	21.000
		75	mg/m2	66.218	8	529.742
		1.5	mg/m2	1.324	3	3.973
		20	mg/m2	17.658	15	264.871
		50	mg/m2	44.145	84	3708.192
		20	mg/m2	17.658	12	211.897
1		11	mg	10.500	1	10.500
		1.5	mg/m2	1.324	4	5.297
		25	mg/m2	22.073	4	88.290
		10,000	IU/m2	8829.028	6	52974.166
		30	mg/m2	26.487	28	741.638
1		11	mg	10.500	3	31.500
		1.5	mg/m2	1.324	4	5.297
		60	mg/m2	52.974	28	1483.277
1		11	mg	10.500	4	42.000
	1	75	mg/m2	66.218	16	1059.483
		25,000	IU/m2	22072.569	4	88290.276
		250	mg/m2	220.726	6	1324.354
		1,000	mg/m2	882.903	2	1765.806
		1.5	mg/m2	1.324	4	5.297
		5,000	gm/m2	4414.514	4	17658.055
1		11	mg	10.500	2	21.000
		25	mg/m2	22.073	56	1236.064
		15	mg/m2	13.244	24	317.845
		1.5	mg/m2	1.324	5	6.622
		25	mg/m2	22.073	3	66.218
		5	mg/m2	4.415	14	61.803
		1,000	mg/m2	882.903	1	882.903
		250	mg/m2	220.726	3	662.177
		60	mg/m2	52.974	14	741.638
		10,000	IU/m2	8829.028	4	35316.111
1		11	mg	10.500	3	31.500
	1	75	mg/m2	66.218	8	529.742
		1.5	mg/m2	1.324	3	3.973

For peer review only

		20	mg/m2	17.658	15	264.871
		50	mg/m2	44.145	84	3708.192
1		11	mg	10.500	1	10.500
		20	mg/m2	17.658	12	211.897
		1.5	mg/m2	1.324	4	5.297
		10	mg/m2	8.829	2	17.658
		10,000	IU/m2	8829.028	6	52974.166
1		11	mg	10.500	2	21.000
		20	mg/m2	17.658	10	176.581
		6	mg/m2	5.297	5	26.487
		1.5	mg/m2	1.324	1	1.324
		1,000	mg/m2	882.903	1	882.903
		15	mg/m2	13.244	6	79.461
		10,000	IU/m2	8829.028	3	26487.083
		440	mg/m2	388.477	5	1942.386
		100	mg/m2	88.290	5	441.451
1		11	mg	10.500	1	10.500
		6	mg/m2	5.297	5	26.487
		2	mg/m2	1.324	1	1.324
		3,000	mg/m2	2648.708	8	21189.666
		10,000	IU/m2	8829.028	5	44145.138
		1,000	mg/m2	882.903	1	882.903
		15	mg/m2	13.244	6	79.461
1		11	mg	10.500	2	21.000
		1.5	mg/m2	1.324	4	5.297
		10	mg/m2	8.829	2	17.658
		10,000	IU/m2	8829.028	6	52974.166
1		11	mg	10.500	2	21.000
		20	mg/m2	17.658	10	176.581
		6	mg/m2	5.297	5	26.487
		1.5	mg/m2	1.324	1	1.324
		1,000	mg/m2	882.903	1	882.903
		15	mg/m2	13.244	6	79.461
		10,000	IU/m2	8829.028	3	26487.083
		440	mg/m2	388.477	5	1942.386
		100	mg/m2	88.290	5	441.451
1		11	mg	10.500	1	10.500
		6	mg/m2	5.297	5	26.487
		2	mg/m2	1.324	1	1.324
		3,000	mg/m2	2648.708	8	21189.666
		10,000	IU/m2	8829.028	5	44145.138
		1,000	mg/m2	882.903	1	882.903
		15	mg/m2	13.244	6	79.461
1		11	mg	10.500	2	21.000
		1,000	mg/m2	882.903	5	4414.514
		6	mg/m2	5.297	5	26.487
		1.5	mg/m2	1.324	1	1.324
		75	mg/m2	66.218	42	2781.144
		20	mg/m2	17.658	4	70.632
		25	mg/m2	22.073	4	88.290
		300	mg/m2	264.871	2	529.742
		150	mg/m2	132.435	2	264.871
	1	50	mg/m2	44.145	8	353.161
1		10.5	mg	10.500	2	21.000
		6	mg/m2	5.297	5	26.487
		1.5	mg/m2	1.324	1	1.324

		75	mg/m2	66.218	525	34764.296
		20	mg/m2	17.658	75	1324.354
1		10.5	mg	10.500	6	63.000
		1.5	mg/m2	1.896	4	7.585
		25	mg/m2	31.605	4	126.418
		10,000	IU/m2	12641.820	6	75850.920
		30	mg/m2	37.925	28	1061.913
1		11	mg	10.500	3	31.500
		1.5	mg/m2	1.896	4	7.585
		60	mg/m2	75.851	28	2123.826
1		11	mg	10.500	4	42.000
	1	75	mg/m2	94.814	16	1517.018
		25,000	IU/m2	31604.550	4	126418.200
		250	mg/m2	316.046	6	1896.273
		1,000	mg/m2	1264.182	2	2528.364
		1.5	mg/m2	1.896	4	7.585
		5,000	gm/m2	6320.910	4	25283.640
1		11	mg	10.500	2	21.000
		25	mg/m2	31.605	56	1769.855
		15	mg/m2	18.963	24	455.106
		1.5	mg/m2	1.896	5	9.481
		25	mg/m2	31.605	3	94.814
		5	mg/m2	6.321	14	88.493
		1,000	mg/m2	1264.182	1	1264.182
		250	mg/m2	316.046	3	948.137
		60	mg/m2	75.851	14	1061.913
		10,000	IU/m2	12641.820	4	50567.280
1		11	mg	10.500	3	31.500
	1	75	mg/m2	94.814	8	758.509
		1.5	mg/m2	1.896	3	5.689
		20	mg/m2	25.284	15	379.255
		50	mg/m2	63.209	84	5309.564
1		11	mg	10.500	1	10.500
		20	mg/m2	25.284	12	303.404
		1.5	mg/m2	1.896	4	7.585
		10	mg/m2	12.642	2	25.284
		10,000	IU/m2	12641.820	6	75850.920
1		11	mg	10.500	2	21.000
		20	mg/m2	25.284	10	252.836
		6	mg/m2	7.585	5	37.925
		1.5	mg/m2	1.896	1	1.896
		1,000	mg/m2	1264.182	1	1264.182
		15	mg/m2	18.963	6	113.776
		10,000	IU/m2	12641.820	3	37925.460
		440	mg/m2	556.240	5	2781.200
		100	mg/m2	126.418	5	632.091
1		11	mg	10.500	1	10.500
		6	mg/m2	7.585	5	37.925
		1.5	mg/m2	1.896	1	1.896
		3,000	mg/m2	3792.546	8	30340.368
		10,000	IU/m2	12641.820	5	63209.100
		1,000	mg/m2	1264.182	1	1264.182
		15	mg/m2	18.963	6	113.776
1		10.50	mg	10.500	2	21.000
		1.5	mg/m2	1.896	4	7.585
		10	mg/m2	12.642	2	25.284

For peer review only

		10,000	IU/m2	12641.820	6	75850.920
1		11	mg	10.500	2	21.000
		20	mg/m2	25.284	10	252.836
		6	mg/m2	7.585	5	37.925
		1.5	mg/m2	1.896	1	1.896
		1,000	mg/m2	1264.182	1	1264.182
		15	mg/m2	18.963	6	113.776
		10,000	IU/m2	12641.820	3	37925.460
		440	mg/m2	556.240	5	2781.200
		100	mg/m2	126.418	5	632.091
1		11	mg	10.500	1	10.500
		6	mg/m2	7.585	5	37.925
		1.5	mg/m2	1.896	1	1.896
		3,000	mg/m2	3792.546	8	30340.368
		10,000	IU/m2	12641.820	5	63209.100
		1000	mg/m2	1264.182	1	1264.182
		15	mg/m2	18.963	6	113.776
1		10.5	mg	10.500	2	21.000
		1000	mg/m2	1264.182	5	6320.910
		6	mg/m2	7.585	5	37.925
		1.5	mg/m2	1.896	1	1.896
		75	mg/m2	94.814	42	3982.173
		20	mg/m2	25.284	4	101.135
		25	mg/m2	31.605	4	126.418
		300	mg/m2	379.255	2	758.509
		150	mg/m2	189.627	2	379.255
	1	50	mg/m2	63.209	8	505.673
1		10.5	mg	10.500	2	21.000
		6	mg/m2	7.585	5	37.925
		1.5	mg/m2	1.896	1	1.896
		75	mg/m2	94.814	525	49777.166
		20	mg/m2	25.284	75	1896.273
1		10.5	mg	10.500	6	63.000

For peer review only

Number of Cycles/Case	Average Amount/Case (mg)	BSA
1	3.326765	0.55
1	33267.65	0.55
1	465.7472	0.55
1	31.5	1.00
1	1.663383	0.55
1	1164.368	0.55
1	21	1.00
1	3.326765	0.55
1	5544.609	0.55
1	199.6059	0.55
1	42	1.00
1	2.495074	0.55
1	41.58457	0.55
1	38.81226	0.55
1	554.4609	0.55
1	465.7472	0.55
1	11089.22	0.55
1	21	1.00
1	332.6765	0.55
9	22.45567	0.55
9	1497.044	0.55
9	20958.62	0.55
9	1197.636	0.55
9	94.5	1.00
1	3.326765	0.55
1	55.44609	0.55
1	33267.65	0.55
1	465.7472	0.55
1	31.5	1.00
1	3.326765	0.55
1	931.4943	0.55
1	42	1.00
1	665.3531	0.55
1	55446.09	0.55
1	831.6914	0.55
1	1108.922	0.55
1	3.326765	0.55
1	11089.22	0.55
1	21	1.00
1	776.2453	0.55
1	199.6059	0.55
1	4.158457	0.55
1	41.58457	0.55
1	38.81226	0.55
1	554.4609	0.55
1	415.8457	0.55
1	465.7472	0.55
1	22178.44	0.55
1	31.5	1.00

	INN	Average Amount/Case (mg) across treatment phases
0-4 years old Standard risk	Asparaginase	44,357
	Cyclophosphamide	554
	Cytarabine	333
	Dexamethasone	39
	Doxorubicin	42
	Leucovorin	200
	Mercaptopurine	22,123
	Methotrexate IV	5,755
	Methotrexate tab	1,198
	Prednisolone	1,963
	Tioguanine	466
	Vincristine	33
0-4 years old High risk	Asparaginase	110,892
	Cyclophosphamide	1,663
	Cytarabine	998
	Dexamethasone	39
	Doxorubicin	97
	Leucovorin	200
	Mercaptopurine	22,666
	MESNA	1,248
	Methotrexate IV	11,310
	Methotrexate tab	1,198
	Prednisolone	1,963
	Tioguanine	466
	Vincristine	37
0-4 years old Relapse SR	Asparaginase	77,625
	Cyclophosphamide	1,885
	Cytarabine	13,751
	Dexamethasone	477
	Etoposide	610
	Leucovorin	100
	Mercaptopurine	25,325
	Methotrexate IV	1,266
	Methotrexate tab	1,031
	Mitoxantrone	11
	Vincristine	22
0-4 years old Relapse HR	Asparaginase	77,625
	Cyclophosphamide	1,220
	Cytarabine	16,079
	Dexamethasone	144
	Etoposide	277
	Leucovorin	100
	Methotrexate IV	1,161
	Mitoxantrone	11

For peer review only

1	332.6765	0.55
9	22.45567	0.55
9	1497.044	0.55
9	20958.62	0.55
9	94.5	1.00
9	1197.636	0.55
1	3.326765	0.55
1	11.08922	0.55
1	33267.65	0.55
1	21	1.00
1	110.8922	0.55
1	16.63383	0.55
1	0.831691	0.55
1	554.4609	0.55
1	49.90148	0.55
1	16633.83	0.55
1	1219.814	0.55
1	277.2305	0.55
1	10.5	1.00
1	16.63383	0.55
1	0.831691	0.55
1	13307.06	0.55
1	27723.05	0.55
1	554.4609	0.55
1	49.90148	0.55
1	21	1.00
1	3.326765	0.55
1	11.08922	0.55
1	33267.65	0.55
1	21	1.00
1	110.8922	0.55
1	16.63383	0.55
1	0.831691	0.55
1	554.4609	0.55
1	49.90148	0.55
1	16633.83	0.55
1	1219.814	0.55
1	277.2305	0.55
1	10.5	1.00
1	16.63383	0.55
1	0.831691	0.55
1	13307.06	0.55
1	27723.05	0.55
1	554.4609	0.55
1	49.90148	0.55
1	21	1.00
1	2772.305	0.55
2	33.26765	0.55
2	1.663383	0.55
2	3493.104	0.55
2	88.71375	0.55
2	110.8922	0.55
2	665.3531	0.55
2	332.6765	0.55
2	443.5687	0.55
2	42	1.00

	Vincristine	5
5-9 years old Standard risk	Asparaginase	70,632.221
	Cyclophosphamide	883
	Cytarabine	530
	Dexamethasone	62
	Doxorubicin	66
	Leucovorin	318
	Mercaptopurine	35,228
	Methotrexate IV	9,039
	Methotrexate tab	1,907
	Prednisolone	3,125
	Tioguanine	742
	Vincristine	53
5-9 years old High risk	Asparaginase	176,581
	Cyclophosphamide	2,649
	Cytarabine	1,589
	Dexamethasone	62
	Doxorubicin	155
	Leucovorin	318
	Mercaptopurine	36,093
	MESNA	1,987
	Methotrexate IV	17,879
	Methotrexate tab	1,907
	Prednisolone	3,125
	Tioguanine	742
	Vincristine	58
5-9 years old Relapse SR	Asparaginase	123,606
	Cyclophosphamide	3,002
	Cytarabine	21,896
	Dexamethasone	759
	Etoposide	971
	Leucovorin	159
	Mercaptopurine	40,327
	Methotrexate IV	1,923
	Methotrexate tab	1,642
	Mitoxantrone	18
	Vincristine	34
5-9 years old Relapse HR	Asparaginase	123,606
	Cyclophosphamide	1,942
	Cytarabine	25,604
	Dexamethasone	230
	Etoposide	441
	Leucovorin	159
	Methotrexate IV	1,818
	Mitoxantrone	18
	Vincristine	8
10-14 years old High risk	Asparaginase	252,836
	Cyclophosphamide	3,793
	Cytarabine	2,276
	Dexamethasone	88

For peer review only

18	299.4089	0.55
18	14.97044	0.55
1	21831.9	0.55
1	831.6914	0.55
1	63	1.00
1	5.297417	0.88
1	52974.17	0.88
1	741.6383	0.88
1	31.5	1.00
1	2.648708	0.88
1	1854.096	0.88
1	21	1.00
1	5.297417	0.88
1	8829.028	0.88
1	317.845	0.88
1	42	1.00
1	3.973062	0.88
1	66.21771	0.88
1	61.80319	0.88
1	882.9028	0.88
1	741.6383	0.88
1	17658.06	0.88
1	21	1.00
1	529.7417	0.88
9	35.75756	0.88
9	2383.837	0.88
9	33373.72	0.88
9	1907.07	0.88
9	94.5	1.00
1	5.297417	0.88
1	88.29028	0.88
1	52974.17	0.88
1	741.6383	0.88
1	31.5	1.00
1	5.297417	0.88
1	1483.277	0.88
1	42	1.00
1	1059.483	0.88
1	88290.28	0.88
1	1324.354	0.88
1	1765.806	0.88
1	5.297417	0.88
1	17658.06	0.88
1	21	1.00
1	1236.064	0.88
1	317.845	0.88
1	6.621771	0.88
1	66.21771	0.88
1	61.80319	0.88
1	882.9028	0.88
1	662.1771	0.88
1	741.6383	0.88
1	35316.11	0.88
1	31.5	1.00
1	529.7417	0.88
9	35.75756	0.88

	Doxorubicin	221
	Leucovorin	455
	Mercaptopurine	51,680
	MESNA	2,844
	Methotrexate IV	25,504
	Methotrexate tab	2,731
	Prednisolone	4,475
	Tioguanine	1,062
	Vincristine	83
10-14 years old Relapse SR	Asparaginase	176,985
	Cyclophosphamide	4,298
	Cytarabine	31,352
	Dexamethasone	1,087
	Etoposide	1,391
	Leucovorin	228
	Mercaptopurine	57,742
	Methotrexate IV	2,686
	Methotrexate tab	2,351
	Mitoxantrone	25
	Vincristine	49
10-14 years old Relapse HR	Asparaginase	176,985
	Cyclophosphamide	2,781
	Cytarabine	36,661
	Dexamethasone	329
	Etoposide	632
	Leucovorin	228
	Methotrexate IV	2,581
	Mitoxantrone	25
	Vincristine	11

For peer review only

9	2383.837	0.88
9	33373.72	0.88
9	94.5	1.00
9	1907.07	0.88
1	5.297417	0.88
1	17.65806	0.88
1	52974.17	0.88
1	21	1.00
1	176.5806	0.88
1	26.48708	0.88
1	1.324354	0.88
1	882.9028	0.88
1	79.46125	0.88
1	26487.08	0.88
1	1942.386	0.88
1	441.4514	0.88
1	10.5	1.00
1	26.48708	0.88
1	1.324354	0.88
1	21189.67	0.88
1	44145.14	0.88
1	882.9028	0.88
1	79.46125	0.88
1	21	1.00
1	5.297417	0.88
1	17.65806	0.88
1	52974.17	0.88
1	21	1.00
1	176.5806	0.88
1	26.48708	0.88
1	1.324354	0.88
1	882.9028	0.88
1	79.46125	0.88
1	26487.08	0.88
1	1942.386	0.88
1	441.4514	0.88
1	10.5	1.00
1	26.48708	0.88
1	1.324354	0.88
1	21189.67	0.88
1	44145.14	0.88
1	882.9028	0.88
1	79.46125	0.88
1	21	1.00
1	4414.514	0.88
2	52.97417	0.88
2	2.648708	0.88
2	5562.287	0.88
2	141.2644	0.88
2	176.5806	0.88
2	1059.483	0.88
2	529.7417	0.88
2	706.3222	0.88
2	42	1.00
18	476.7675	0.88
18	23.83837	0.88

For peer review only

1	34764.3	0.88
1	1324.354	0.88
1	63	1.00
1	7.585092	1.26
1	126.4182	1.26
1	75850.92	1.26
1	1061.913	1.26
1	31.5	1.00
1	7.585092	1.26
1	2123.826	1.26
1	42	1.00
1	1517.018	1.26
1	126418.2	1.26
1	1896.273	1.26
1	2528.364	1.26
1	7.585092	1.26
1	25283.64	1.26
1	21	1.00
1	1769.855	1.26
1	455.1055	1.26
1	9.481365	1.26
1	94.81365	1.26
1	88.49274	1.26
1	1264.182	1.26
1	948.1365	1.26
1	1061.913	1.26
1	50567.28	1.26
1	31.5	1.00
1	758.5092	1.26
9	51.19937	1.26
9	3413.291	1.26
9	47786.08	1.26
9	94.5	1.00
9	2730.633	1.26
1	7.585092	1.26
1	25.28364	1.26
1	75850.92	1.26
1	21	1.00
1	252.8364	1.26
1	37.92546	1.26
1	1.896273	1.26
1	1264.182	1.26
1	113.7764	1.26
1	37925.46	1.26
1	2781.2	1.26
1	632.091	1.26
1	10.5	1.00
1	37.92546	1.26
1	1.896273	1.26
1	30340.37	1.26
1	63209.1	1.26
1	1264.182	1.26
1	113.7764	1.26
1	21	1.00
1	7.585092	1.26
1	25.28364	1.26

For peer review only

1	75850.92	1.26
1	21	1.00
1	252.8364	1.26
1	37.92546	1.26
1	1.896273	1.26
1	1264.182	1.26
1	113.7764	1.26
1	37925.46	1.26
1	2781.2	1.26
1	632.091	1.26
1	10.5	1.00
1	37.92546	1.26
1	1.896273	1.26
1	30340.37	1.26
1	63209.1	1.26
1	1264.182	1.26
1	113.7764	1.26
1	21	1.00
1	6320.91	1.26
2	75.85092	1.26
2	3.792546	1.26
2	7964.347	1.26
2	202.2691	1.26
2	252.8364	1.26
2	1517.018	1.26
2	758.5092	1.26
2	1011.346	1.26
2	42	1.00
18	682.6583	1.26
18	34.13291	1.26
1	49777.17	1.26
1	1896.273	1.26
1	63	1.00

For peer review only

DX 2017	DX + uDX 2017	DX 2021	DX + uDX 2021	
4,198,059	7,052,242	3,823,511	6,382,478	
52,476	88,153	47,794	79,781	Asparaginase
31,485	52,892	28,676	47,869	Cyclophosphamide
3,673	6,171	3,346	5,585	Cytarabine
3,936	6,611	3,585	5,984	Doxorubicin
18,891	31,735	17,206	28,721	Etoposide
2,093,782	3,517,306	1,906,976	3,183,261	Mercaptopurine
544,632	914,918	496,041	828,026	Methotrexate IV
113,348	190,411	103,235	172,327	Methotrexate tab
185,764	312,062	169,190	282,425	Mitoxantrone
44,080	74,049	40,147	67,016	Tioguanine
3,149	5,289	2,868	4,787	Vincristine
5,677,702	9,537,868	5,171,142	8,632,040	Dexamethasone
85,166	143,068	77,567	129,481	Leucovorin
51,099	85,841	46,540	77,688	MESNA
1,987	3,338	1,810	3,021	Prednisolone
4,968	8,346	4,525	7,553	
10,220	17,168	9,308	15,538	Antineoplastic agent
1,160,522	1,949,540	1,056,981	1,764,389	Chemoprotective ag
63,874	107,301	58,175	97,110	
579,060	972,752	527,397	880,368	**Total expenditure on a**
61,319	103,009	55,848	93,226	Total health expendi
100,495	168,820	91,529	152,787	Percentage of total h
23,846	40,059	21,719	36,255	
1,874	3,147	1,706	2,849	
687,771	1,155,374	626,408	1,045,646	
16,703	28,059	15,213	25,394	
121,834	204,666	110,964	185,229	
4,225	7,097	3,848	6,423	
5,404	9,078	4,922	8,216	
884	1,485	805	1,344	
224,385	376,941	204,366	341,142	
11,221	18,850	10,220	17,059	
9,138	15,350	8,322	13,892	
98	165	89	149	
192	322	174	291	
1,767,540	2,969,259	1,609,842	2,687,263	
27,776	46,660	25,298	42,228	
366,133	615,061	333,467	556,647	
3,283	5,514	2,990	4,991	
6,313	10,604	5,749	9,597	
2,273	3,818	2,070	3,455	
26,446	44,426	24,087	40,207	
253	424	230	384	

For peer review only

114	191	103	173
4,788,237	8,005,361	4,538,249	7,644,633
59,853	100,067	56,728	95,558
35,912	60,040	34,037	57,335
4,190	7,005	3,971	6,689
4,489	7,505	4,255	7,167
21,547	36,024	20,422	34,401
2,388,133	3,992,674	2,263,452	3,812,761
612,766	1,024,471	580,774	978,308
129,282	216,145	122,533	206,405
211,879	354,237	200,818	338,275
50,276	84,056	47,652	80,269
3,591	6,004	3,404	5,733
6,475,895	10,826,922	6,137,795	10,339,052
97,138	162,404	92,067	155,086
58,283	97,442	55,240	93,051
2,267	3,789	2,148	3,619
5,666	9,474	5,371	9,047
11,657	19,488	11,048	18,610
1,323,673	2,213,023	1,254,565	2,113,302
72,854	121,803	69,050	116,314
655,676	1,096,212	621,444	1,046,816
69,940	116,931	66,288	111,662
114,623	191,637	108,639	183,001
27,199	45,473	25,779	43,424
2,137	3,573	2,025	3,412
784,460	1,311,524	743,504	1,252,425
19,051	31,851	18,057	30,416
138,962	232,327	131,706	221,858
4,819	8,057	4,567	7,693
6,164	10,305	5,842	9,840
1,009	1,686	956	1,610
255,930	427,885	242,568	408,604
12,206	20,407	11,569	19,488
10,422	17,425	9,878	16,639
112	187	106	179
219	365	207	349
2,016,028	3,370,557	1,910,773	3,218,677
31,680	52,966	30,026	50,579
417,606	698,187	395,803	666,726
3,744	6,260	3,549	5,978
7,200	12,038	6,824	11,495
2,592	4,334	2,457	4,138
29,657	49,582	28,108	47,348
288	482	273	460
130	217	123	207
17,082,386	28,616,529	16,425,011	27,285,599
256,236	429,248	246,375	409,284
153,741	257,549	147,825	245,570
5,979	10,016	5,749	9,550

For peer review only

14,947	25,039	14,372	23,875
30,748	51,510	29,565	49,114
3,491,640	5,849,219	3,357,272	5,577,176
192,177	321,936	184,781	306,963
1,723,136	2,886,610	1,656,825	2,752,356
184,490	309,059	177,390	294,684
302,358	506,513	290,723	482,955
71,746	120,189	68,985	114,600
5,637	9,443	5,420	9,004
726,450	1,216,954	698,494	1,160,355
17,642	29,555	16,963	28,180
128,685	215,575	123,733	205,549
4,462	7,476	4,291	7,128
5,708	9,562	5,488	9,117
934	1,565	898	1,492
237,004	397,031	227,884	378,566
11,024	18,468	10,600	17,609
9,651	16,168	9,280	15,416
104	174	100	166
202	339	195	323
1,866,944	3,127,517	1,795,099	2,982,059
29,338	49,147	28,209	46,861
386,724	647,843	371,842	617,712
3,467	5,808	3,334	5,538
6,668	11,170	6,411	10,650
2,400	4,021	2,308	3,834
27,224	45,607	26,177	43,485
267	447	256	426
120	201	115	192

For peer review only

For peer review only

For peer review only

For peer review only

Smallest unit	Total mg		Total smallest units		Ex[illegible]
	DX	DX+uDX	DX	DX+uDX	DX
10,000	46,071,471	77,190,107	4,607	7,719	225,847
500	693,059	1,161,177	1,386	2,322	6,551
100	1,890,465	3,167,422	18,905	31,674	73,572
10	34,006	56,975	3,401	5,698	9,705
100	37,456	62,756	375	628	2,112
50	11,175,069	18,723,618	223,501	374,472	164,313
50	4,233,049	7,092,303	84,661	141,846	193,746
2.5	587,590	984,496	235,036	393,798	19,285
10	1,121	1,879	112	188	11,586
40	217,147	363,826	5,429	9,096	12,011
1	17,363	29,092	17,363	29,092	70,469
2	42,096	70,530	21,048	35,265	295
30	103,155	172,834	3,438	5,761	21,030
400	328,905	551,040	822	1,378	2,565
5	915,120	1,533,268	183,024	306,654	1,863

[illegible]s	789,198
ents	25,754
**antineoplasti and chemoprotective agents**	**814,952**
iture (in million constant (2017) US$)	17,055
health budget	0.005

For peer review only

For peer review only

For peer review only

For peer review only

For peer review only

For peer review only

**penditure (US$)**		**Total mg**		**Total smallest units**	
DX+uDX	Proportion	DX	DX+uDX	DX	DX+uDX
378,394	0.28	43,479,829	72,630,227	4,348	7,263
10,976	0.01	654,297	1,092,848	1,309	2,186
123,268	0.09	1,779,834	2,975,235	17,798	29,752
16,260	0.01	32,106	53,625	3,211	5,363
3,538	0.00	35,236	58,916		589
275,303	0.20	10,514,065	17,579,200	210,281	351,584
324,614	0.24	3,993,241	6,671,071	79,865	133,421
32,311	0.02	552,774	924,252	221,110	369,701
19,413	0.01	1,055	1,764	105	176
20,125	0.01	204,281	341,563	5,107	8,539
118,070	0.09	16,341	27,320	16,341	27,320
494	0.00	39,601	66,214	19,801	33,107
35,236	0.03	97,043	162,258	3,235	5,409
4,298	0.00	312,007	520,388	780	1,301
3,122	0.00	860,899	1,439,443	172,180	287,889
1,322,272					
43,149					
**1,365,422**					
0.008					

For peer review only

For peer review only

For peer review only

For peer review only

For peer review only

For peer review only

Expenditure (US$)	
DX	DX+uDX
236,209	394,571
6,854	11,448
76,763	128,319
10,155	16,961
2,202	3,681
171,324	286,448
202,549	338,377
20,105	33,617
12,081	20,199
12,522	20,938
73,507	122,891
307	514
21,928	36,663
2,401	4,004
1,943	3,249
824,270	1,377,449
26,579	44,430
**850,849**	**1,421,880**

For peer review only

Children

Applied value				
	0-4	5-9	10-14	15-19
**Age group**	1	2	3	4
**Applied BSA**	0.554	0.883	1.264	1.532
**Applied weight**	12.425	23.540	39.305	52.135

Adults

Applied value		
	**Male**	**Female**
BSA	1.80	1.58
weight	68.83	57.40

Age	Boy		Girl		Both Sex
	Weight (kg	leight (cm	Veight (kg	leight (cm	Veight (kg
1	9.65	75.6	9.1	73.85	9.375
2	12.45	87	11.7	85.35	12.075
3	14.65	95.1	14	93.65	14.325
4	16.75	101.85	11.1	100.95	13.925
5	18.8	108.55	18.05	107.5	18.425
6	21	114.5	20.4	114.1	20.7
7	23.55	120.1	23.2	119.6	23.375
8	26.15	125.3	25.9	124.7	26.025
9	29.05	130.05	29.3	130.5	29.175
10	32.2	134.8	32.75	136.6	32.475
11	35.4	139.95	36.3	142.75	35.85
12	39.05	146	39.8	147.85	39.425
13	41.6	152.65	43.05	151.85	42.325
14	47.15	158.65	45.75	154.65	46.45

For peer review only

For peer review only - http://bmjopen.bmj.com/site/about/guidelines.xhtml

15	51	163.35	47.55	155.95	49.275
16	54	167.1	48.65	156.55	51.325
17	56.05	168.8	49.2	156.85	52.625
18	57.5	169.45	49.5	157	53.5
19	58.15	169.65	49.75	157	53.95

Sources: Weight and heigh of Thai children [Report on the Thai population Health: 2000.
Mosteller R. Simplified Calculation of Body-Surface Area. N Engl J Med. 1987 Oct 22;317(

Average Tł	Weight (kg	Height (cm)
Male	68.83	169.46
Female	57.4	157

For peer review only

General Business

ตารางแสดงน้ำหนักและส่วนสูงที่ควรจะเป็นในช่วงอายุ 0-19 ปีตามเกณฑ์มาตรฐานการเจริญเติบโตของเพศชายและเพศหญิง (อ้างอิงจากกรมอนามัย กระทรวงสาธารณสุข 2543)

For peer review only

General Business

Body Surface Area					
	The Mosteller formula			The Dubois formula	
AGE	Boy	Girl	Both	Boy	Girl
1	0.450	0.432	0.441	0.433	0.415
2	0.549	0.527	0.538	0.535	0.513
3	0.622	0.603	0.613	0.611	0.593
4	0.688	0.558	0.626	0.680	0.567
5	0.753	0.734	0.744	0.748	0.730
6	0.817	0.804	0.811	0.815	0.803
7	0.886	0.878	0.882	0.885	0.877
8	0.954	0.947	0.951	0.955	0.947
9	1.024	1.031	1.028	1.025	1.032
10	1.098	1.115	1.106	1.100	1.118
11	1.173	1.200	1.186	1.176	1.206
12	1.258	1.279	1.268	1.265	1.287
13	1.328	1.348	1.338	1.342	1.356
14	1.441	1.402	1.422	1.455	1.410
15	1.521	1.435	1.478	1.537	1.442
16	1.583	1.455	1.519	1.601	1.460
17	1.621	1.464	1.543	1.638	1.470
18	1.645	1.469	1.557	1.661	1.474
19	1.655	1.473	1.564	1.670	1.478

Average Thai population	
Male	1.800
Female	1.582

1) The Mosteller formula

Aged Gr	1
	0-4 yrs
BSA	0.554
weight	12.425
high	89.169

3) The Haycock formula

Aged Gr	1
	0-4 yrs
BSA	0.557
weight	12.425
high	89.169

5) The Haycock formula

Aged Gr	1
	0-4 yrs
BSA	0.573
weight	12.425
high	89.169

leight (cm)
74.725
86.175
94.375
101.4
108.025
114.3
119.85
125
130.275
135.7
141.35
146.925
152.25
156.65

Age	Aged Gr	Formula 1
0-4 yr	1	0.554
5-9 yr	2	0.883
10-14 yr	3	1.264
15-19 yr	4	1.532

For peer review only

General Business

159.65
161.825
162.825
163.225
163.325

Ministry of Public Health] (in Thai) [Internet]. Available from: https://www.hiso.or.th/hiso
(17):1098–1098.

For peer review only

For peer review only - http://bmjopen.bmj.com/site/about/guidelines.xhtml

For peer review only

General Business

The Haycock formula			The Gehan formula			The Boyd form	
Both	Boy	Girl	Both	Boy	Girl	Both	Boy
0.424	0.456	0.438	0.447	0.469	0.451	0.460	0.472
0.524	0.553	0.531	0.542	0.568	0.545	0.556	0.570
0.602	0.625	0.607	0.616	0.641	0.622	0.631	0.643
0.626	0.691	0.552	0.624	0.707	0.570	0.641	0.708
0.739	0.754	0.734	0.744	0.770	0.751	0.761	0.771
0.809	0.817	0.803	0.810	0.834	0.820	0.827	0.834
0.881	0.885	0.877	0.881	0.903	0.894	0.898	0.903
0.951	0.953	0.946	0.949	0.970	0.963	0.967	0.970
1.029	1.023	1.029	1.026	1.040	1.046	1.043	1.041
1.109	1.097	1.113	1.105	1.113	1.129	1.121	1.114
1.191	1.171	1.197	1.184	1.188	1.213	1.200	1.188
1.276	1.256	1.275	1.265	1.272	1.291	1.281	1.271
1.349	1.322	1.344	1.333	1.339	1.360	1.349	1.334
1.433	1.436	1.399	1.418	1.451	1.414	1.433	1.446
1.489	1.516	1.433	1.474	1.530	1.447	1.489	1.524
1.530	1.577	1.453	1.515	1.591	1.467	1.529	1.583
1.554	1.615	1.463	1.539	1.629	1.476	1.553	1.621
1.567	1.640	1.468	1.555	1.653	1.482	1.568	1.646
1.574	1.651	1.472	1.562	1.663	1.485	1.575	1.656
1.793			1.807			1.813	
1.570			1.590			1.599	

2) The Dubois formula							
2	3	4	Aged Gr	1	2	3	4
5-9 yrs	10-14 yr	15-19 yr		0-4 yrs	5-9 yrs	10-14 yr	15-19 yr
0.883	1.264	1.532	BSA	0.544	0.882	1.271	1.543
23.540	39.305	52.135	weight	12.425	23.540	39.305	52.135
119.490	146.575	162.170	high	89.169	119.490	146.575	162.170

4) The Boyd formula							
2	3	4	Aged Gr	1	2	3	4
5-9 yrs	10-14 yr	15-19 yr		0-4 yrs	5-9 yrs	10-14 yr	15-19 yr
0.882	1.261	1.529	BSA	0.572	0.899	1.277	1.542
23.540	39.305	52.135	weight	12.425	23.540	39.305	52.135
119.490	146.575	162.170	high	89.169	119.490	146.575	162.170

2	3	4
5-9 yrs	10-14 yr	15-19 yr
0.899	1.275	1.539
23.540	39.305	52.135
119.490	146.575	162.170

BSA				Weight	Height
Formula 2	Formula 3	Formula 4	Formula 5		
0.544	0.557	0.572	0.573	12.425	89.169
0.882	0.882	0.899	0.899	23.540	119.490
1.271	1.261	1.277	1.275	39.305	146.575
1.543	1.529	1.542	1.539	52.135	162.170

For peer review only - http://bmjopen.bmj.com/site/about/guidelines.xhtml

o/picture/reportHealth/report/report6.pdf (last accessed 9 May 2020)

For peer review only

General Business

For peer review only

General Business

nula	
Girl	Both
0.453	0.463
0.547	0.559
0.623	0.633
0.558	0.637
0.751	0.761
0.820	0.827
0.894	0.898
0.963	0.967
1.047	1.044
1.129	1.122
1.212	1.200
1.289	1.280
1.357	1.346
1.411	1.429
1.446	1.485
1.466	1.525
1.476	1.549
1.481	1.564
1.485	1.571

For peer review only

For peer review only - http://bmjopen.bmj.com/site/about/guidelines.xhtml

INN	Formulation	Form	Brand name
Asparaginase	ASPARAGINASE VIAL DRY 0.01 M		LEUNASE
Cyclophosphamide	CYCLOPHOSPHAMIDE VIAL DRY 1 G	IV	ENDOXAN
Cyclophosphamide	CYCLOPHOSPHAMIDE VIAL DRY 1 G	IV	CYCLOXAN
Cyclophosphamide	CYCLOPHOSPHAMIDE VIAL DRY 200 MG	IV	ENDOXAN
Cyclophosphamide	CYCLOPHOSPHAMIDE VIAL DRY 200 MG	IV	CYCLOXAN
Cyclophosphamide	CYCLOPHOSPHAMIDE VIAL DRY 500 MG	IV	ENDOXAN
Cyclophosphamide	CYCLOPHOSPHAMIDE VIAL DRY 500 MG	IV	ONCOMIDE
Cytarabine	CYTARABINE VIAL 1 G /10ML (10 ML)	IV	CYTARINE
Cytarabine	CYTARABINE VIAL 100 MG (1 ML)	IV	CYTARINE
Cytarabine	CYTARABINE VIAL 100 MG /ML (20 ML)	IV	CYTOSAR CS
Cytarabine	CYTARABINE VIAL 20 MG /ML (5 ML)	IV	CYTOSAR CS
Cytarabine	CYTARABINE VIAL 500 MG (5 ML)	IV	CYTARINE
Doxorubicin	DOXORUBICIN VIAL 10 MG /5ML (5 ML)	IV	ADRIM
Doxorubicin	DOXORUBICIN VIAL 2 MG /ML (25 ML)	IV	DOXORUBIN
Doxorubicin	DOXORUBICIN VIAL 2 MG /ML (5 ML)	IV	DOXORUBIN
Doxorubicin	DOXORUBICIN VIAL 50 MG (25 ML)	IV	DOXORUBICIN
Doxorubicin	DOXORUBICIN VIAL 50 MG /25ML (25 ML	IV	ADRIM
Etoposide	ETOPOSIDE VIAL 100 MG (5 ML)	IV	ETOPOS
Etoposide	ETOPOSIDE VIAL 100 MG /5ML (5 ML)	IV	EPOSIN
Etoposide	ETOPOSIDE VIAL 100 MG /5ML (5 ML)	IV	FYTOSID
Etoposide	ETOPOSIDE VIAL 100 MG /5ML (5 ML)	IV	SEDOL
Mercaptopurine	MERCAPTOPURINE TAB 50 MG (25 tab)	tab	PURI-NETHOL
Mercaptopurine	MERCAPTOPURINE TAB 50 MG (100 tab)	tab	PURINETONE
MESNA	Mesna 400mg/4ml Injection	IV	
Methotrexate	METHOTREXATE VIAL 1000 MG (10 ML)	IV	EMTHEXATE
Methotrexate	METHOTREXATE VIAL 1000 MG (10 ML)	IV	ALLTREX
Methotrexate	METHOTREXATE VIAL 50 MG /2ML (2 ML	IV	BIOTREXATE
Methotrexate	METHOTREXATE VIAL 50 MG /2ML (2 ML	IV	EMTHEXATE
Methotrexate	METHOTREXATE VIAL 50 MG /2ML (2 ML	IV	ALLTREX
Methotrexate	METHOTREXATE TAB 2.5 MG	tab	BIOTREXATE
Methotrexate	METHOTREXATE TAB 2.5 MG	tab	EMTHEXATE
Methotrexate	METHOTREXATE TAB 2.5 MG	tab	METREX
Methotrexate	METHOTREXATE TAB 2.5 MG	tab	BIOTREXATE
Methotrexate	METHOTREXATE TAB 2.5 MG	tab	ONKOMET
Methotrexate	METHOTREXATE TAB 2.5 MG	tab	UNITREXATE
Methotrexate	METHOTREXATE TAB SC 2.5 MG	tab	METHOTREXA
Tioguanine	TIOGUANINE TAB 40 MG	tab	LANVIS
Vincristine	VINCRISTINE VIAL 1 MG /ML (1 ML)	IV	V.C.S.
Dexamethasone	Dexamethasone tab 0.5 mg	tab	
Leucovorin	Calcium folinate (leucovorin calcium) ster	IV	
Mitoxantrone	Mitoxantrone hydrochloride sterile sol 1(	IV	
Mitoxantrone	Mitoxantrone hydrochloride sterile sol 2(	IV	
Prednisolone	Prednisolone tab 5 mg	tab	

For peer review only

Source: Pharmaceutical Information Center Ministry of Public Health as announced on December 9,
http://dmsic.moph.go.th/index/drugsearch/3

Exchange rates Official exchange rate (LCU per US$, period average)
Source: https://data.worldbank.org/indicator/PA.NUS.FCRF (accessed 29 May 2020) 2020 data not y

Thailand	Inflation (%)	
2017	0.67	
2018	1.06	
2019	0.71	
2020	-1.06	*forecasted
2021	0.56	*forecasted

Source: https://www.statista.com/statistics/332274/inflation-rate-in-thailand/ (accessed 28 May 20

For peer review only

Manufacturer	strength	Units per pac	ng (or units)	Year	e local curre	ocal currenc
KYOWA HAKKO KO	10,000	1	10000	2018	1,675	THB
BAXTER HEALTHCA	1,000	1	1,000	2018	423	THB
BIOCHEM GES.M B	1,000	1	1,000	2018	200	THB
BAXTER HEALTHCA	200	10	2,000	2018	877	THB
BIOCHEM GES.M B	200	1	200	2018	50	THB
BAXTER HEALTHCA	500	1	500	2018	203	THB
BORYUNG	500	1	500	2018	110	THB
FRESENIUS	1,000	1	1,000	2018	879	THB
FRESENIUS	100	1	100	2018	129	THB
PFIZER INTER. CORI	2,000	1	2,000	2018	2,825	THB
PFIZER INTER. CORI	100	1	100	2018	205	THB
FRESENIUS	500	1	500	2018	508	THB
FRESENIUS	10	1	10	2018	118	THB
PHARMACHEMIE B.	50	1	50	2018	546	THB
PHARMACHEMIE B.	10	1	10	2018	139	THB
KHANDELWAL	50	1	50	2018	330	THB
FRESENIUS	50	1	50	2018	278	THB
TEVA INT'L	100	1	100	2018	189	THB
PHARMACHEMIE B.	100	1	100	2018	250	THB
FRESENIUS	100	1	100	2018	171	THB
VENUS REMEDIES	100	1	100	2018	160	THB
ASPEN PHARMACA	50	25	1,250	2018	856	THB
KOREA UNITED PH/	50	100	5,000	2018	1,600	THB
	400	1	400	2019	8.7	RM
PHARMACHEMIE B.	1,000	1	1,000	2018	975	THB
NAPROD LIFE SCIEN	1,000	1	1,000	2018	945	THB
BIOCHEM GES.M B	50	1	50	2018	95	THB
PHARMACHEMIE B.	50	1	50	2018	110	THB
NAPROD LIFE SCIEN	50	1	50	2018	90	THB
BIOCHEM GES.MBH	2.5	100	250	2018	207	THB
PHARMACHEMIE BY	2.5	100	250	2018	321	THB
DAE HAN NEW PHA	2.5	100	250	2018	270	THB
BIOCHEM GES.MBH	2.5	50	125	2018	94	THB
KLAB	2.5	100	250	2018	240	THB
KENYAKU LTD	2.5	100	250	2018	247	THB
REMEDICA	2.5	100	250	2018	490	THB
ASPEN PHARMACA	40	25	1,000	2018	1,890	THB
BORYUNG	1	1	1	2019	140	THB
	0.5	1,000	500	2019	120.91	THB
	50	1	50	2019	352.03	THB
	10	1	10	2019	3,954.72	THB
	20	1	20	2019	6,364.36	THB
	5	500	2,500	2019	175.81	THB

For peer review only

2019

yet available, we used 2019

)20)

For peer review only

xchange rat	Price USD	Price per mg	Notes		price_mg_USD_201
32.31	52	0.005		Asparaginase	0.0049021
32.31	13	0.013		Cyclophosphamide	0.0094528
32.31	6	0.006		Cytarabine	0.0389176
32.31	27	0.014		Doxorubicin	0.2853954
32.31	2	0.008		Etoposide	0.0563812
32.31	6	0.013		Mercaptopurine	0.0147035
32.31	3	0.007		Methotrexate IV	0.0457699
32.31	27	0.027		Methotrexate tab	0.0328201
32.31	4	0.040		Mitoxantrone	10.33291
32.31	87	0.044		Tioguanine	0.0553137
32.31	6	0.064		Vincristine	4.058514
32.31	16	0.031			
32.31	4	0.364		Dexamethasone	0.0070022
32.31	17	0.338		Leucovorin	0.2038696
32.31	4	0.431		MESNA	0.0077992
32.31	10	0.204		Prednisolone	0.0020363
32.31	9	0.172			
32.31	6	0.059			
32.31	8	0.077			
32.31	5	0.053			
32.31	5	0.050			
32.31	26	0.021			
32.31	50	0.010			
3.9055	2	0.006	We could not find the price for MESNA in Thailand so we used the pric		
32.31	30	0.030			
32.31	29	0.029			
32.31	3	0.059			
32.31	3	0.068			
32.31	3	0.056			
32.31	6	0.026			
32.31	10	0.040			
32.31	8	0.033			
32.31	3	0.023			
32.31	7	0.030			
32.31	8	0.031			
32.31	15	0.061			
32.31	58	0.058			
32.31	4	4.338			
32.31	3.7	0.007			
32.31	10.9	0.218			
32.31	122.4	12.240			
32.31	197	9.849			
32.31	5	0.002			

For peer review only

For peer review only

price_mg_USD_2021
0.00543
0.01048
0.04313
0.31628
0.06248
0.01629
0.05072
0.03637
11.45239
0.06130
4.49822
0.00776
0.22596
0.00770
0.00226

ce published in the Philippines (https://dpri.doh.gov.ph/download/2019%20DPRI%20Fourth%20Editi

For peer review only

For peer review only

ion.pdf, accessed 10 May 2020)

For peer review only

Sensitivity analysis

1) Relapse 50%, relapse 0.5 SR: 0.5 HR	Diagnosed	Underdiagnosed
Total expenditure (US$)	1,015,410	1,701,286
Percentage of total health budget (%)	0.006%	0.010%
Asparaginase (smallest units)	5,632	9,436
Mercaptopurine (smallest units)	268,192	449,351

2) Incidence UB	Diagnosed	Underdiagnosed
Total expenditure (US$)	1,117,398	2,031,973
Percentage of total health budget (%)	0.007%	0.012%
Asparaginase (smallest units)	6,323	11,480
Mercaptopurine (smallest units)	306,075	557,666

3) Incidence UB, relapse 50%	Diagnosed	Underdiagnosed
Total expenditure (US$)	1,362,849	2,479,242
Percentage of total health budget (%)	0.008%	0.015%
Asparaginase (smallest units)	7,726	14,037
Mercaptopurine (smallest units)	331,720	604,396

For peer review only

Total health expenditure (in million constant (2017) US$
17054.69

For peer review only

;)

For peer review only

STROBE Statement—checklist of items that should be included in reports of observational studies

	Item No	Recommendation	Page No
**Title and abstract**	1	(*a*) Indicate the study's design with a commonly used term in the title or the abstract	1,3
		(*b*) Provide in the abstract an informative and balanced summary of what was done and what was found	3
**Introduction**			
Background/rationale	2	Explain the scientific background and rationale for the investigation being reported	5-6
Objectives	3	State specific objectives, including any prespecified hypotheses	6
**Methods**			
Study design	4	Present key elements of study design early in the paper	6-7
Setting	5	Describe the setting, locations, and relevant dates, including periods of recruitment, exposure, follow-up, and data collection	6-7
Participants	6	(*a*) *Cohort study*—Give the eligibility criteria, and the sources and methods of selection of participants. Describe methods of follow-up<br>*Case-control study*—Give the eligibility criteria, and the sources and methods of case ascertainment and control selection. Give the rationale for the choice of cases and controls<br>*Cross-sectional study*—Give the eligibility criteria, and the sources and methods of selection of participants	6-7
		(*b*) *Cohort study*—For matched studies, give matching criteria and number of exposed and unexposed<br>*Case-control study*—For matched studies, give matching criteria and the number of controls per case	n/a
Variables	7	Clearly define all outcomes, exposures, predictors, potential confounders, and effect modifiers. Give diagnostic criteria, if applicable	8-9
Data sources/ measurement	8*	For each variable of interest, give sources of data and details of methods of assessment (measurement). Describe comparability of assessment methods if there is more than one group	6-8
Bias	9	Describe any efforts to address potential sources of bias	9
Study size	10	Explain how the study size was arrived at	6-7
Quantitative variables	11	Explain how quantitative variables were handled in the analyses. If applicable, describe which groupings were chosen and why	8-9
Statistical methods	12	(*a*) Describe all statistical methods, including those used to control for confounding	n/a
		(*b*) Describe any methods used to examine subgroups and interactions	n/a
		(*c*) Explain how missing data were addressed	n/a
		(*d*) *Cohort study*—If applicable, explain how loss to follow-up was addressed<br>*Case-control study*—If applicable, explain how matching of cases and controls was addressed<br>*Cross-sectional study*—If applicable, describe analytical methods taking account of sampling strategy	n/a
		(*e*) Describe any sensitivity analyses	8

Continued on next page

For peer review only

For peer review only - http://bmjopen.bmj.com/site/about/guidelines.xhtml

**Results**			
Participants	13*	(a) Report numbers of individuals at each stage of study—eg numbers potentially eligible, examined for eligibility, confirmed eligible, included in the study, completing follow-up, and analysed	9-10
		(b) Give reasons for non-participation at each stage	n/a
		(c) Consider use of a flow diagram	n/a
Descriptive data	14*	(a) Give characteristics of study participants (eg demographic, clinical, social) and information on exposures and potential confounders	9-10
		(b) Indicate number of participants with missing data for each variable of interest	n/a
		(c) *Cohort study*—Summarise follow-up time (eg, average and total amount)	n/a
Outcome data	15*	*Cohort study*—Report numbers of outcome events or summary measures over time	n/a
		Case-control study—Report numbers in each exposure category, or summary measures of exposure	n/a
		Cross-sectional study—Report numbers of outcome events or summary measures	9-10
Main results	16	(*a*) Give unadjusted estimates and, if applicable, confounder-adjusted estimates and their precision (eg, 95% confidence interval). Make clear which confounders were adjusted for and why they were included	9-10
		(*b*) Report category boundaries when continuous variables were categorized	9-10
		(*c*) If relevant, consider translating estimates of relative risk into absolute risk for a meaningful time period	n/a
Other analyses	17	Report other analyses done—eg analyses of subgroups and interactions, and sensitivity analyses	10
**Discussion**			
Key results	18	Summarise key results with reference to study objectives	10-11
Limitations	19	Discuss limitations of the study, taking into account sources of potential bias or imprecision. Discuss both direction and magnitude of any potential bias	11-12
Interpretation	20	Give a cautious overall interpretation of results considering objectives, limitations, multiplicity of analyses, results from similar studies, and other relevant evidence	11-12
Generalisability	21	Discuss the generalisability (external validity) of the study results	
**Other information**			
Funding	22	Give the source of funding and the role of the funders for the present study and, if applicable, for the original study on which the present article is based	13-14

*Give information separately for cases and controls in case-control studies and, if applicable, for exposed and unexposed groups in cohort and cross-sectional studies.

Note: An Explanation and Elaboration article discusses each checklist item and gives methodological background and published examples of transparent reporting. The STROBE checklist is best used in conjunction with this article (freely available on the Web sites of PLoS Medicine at http://www.plosmedicine.org/, Annals of Internal Medicine at http://www.annals.org/, and Epidemiology at http://www.epidem.com/). Information on the STROBE Initiative is available at www.strobe-statement.org.

For peer review only

BMJ Open

What are the volume and budget needs to provide chemotherapy to all children with acute lymphoblastic leukemia in Thailand? Development and application of an estimation tool

Journal:	*BMJ Open*
Manuscript ID	bmjopen-2020-041901.R1
Article Type:	Original research
Date Submitted by the Author:	25-Aug-2020
Complete List of Authors:	Sruamsiri, Rosarin; Naresuan University, Center of Pharmaceutical Outcomes Research<br>Ferrario, Alessandra; Harvard Medical School, Population Medicine; Harvard Pilgrim Health Care Institute, Population Medicine<br>Ross-Degnan, Dennis; Harvard Medical School, Department of Population Medicine; Harvard Pilgrim Health Care Institute, Department of Population Medicine<br>Denburg, Avram; Hospital for Sick Children, Child Health and Evaluative Sciences; The Hospital for Sick Children<br>Frazier, A. Lindsay; Dana-Farber/Boston Children's Cancer and Blood Disorders Center<br>Gupta, Sumit; Hospital for Sick Children, Child Health Evaluative Sciences; Hospital for Sick Children, Division of Haematology/Oncology<br>Ward, Zachary; Harvard University T H Chan School of Public Health, Center for Health Decision Science<br>Yeh, Jennifer; Harvard Medical School, Department of Pediatrics<br>Wagner, Anita; Harvard Medical School, Department of Population Medicine; Harvard Pilgrim Health Care Institute, Department of Population Medicine
**Primary Subject Heading**:	Oncology
Secondary Subject Heading:	Paediatrics, Global health
Keywords:	Leukaemia < ONCOLOGY, Paediatric oncology < ONCOLOGY, Health policy < HEALTH SERVICES ADMINISTRATION & MANAGEMENT

SCHOLARONE™
Manuscripts

I, the Submitting Author has the right to grant and does grant on behalf of all authors of the Work (as defined in the below author licence), an exclusive licence and/or a non-exclusive licence for contributions from authors who are: i) UK Crown employees; ii) where BMJ has agreed a CC-BY licence shall apply, and/or iii) in accordance with the terms applicable for US Federal Government officers or employees acting as part of their official duties; on a worldwide, perpetual, irrevocable, royalty-free basis to BMJ Publishing Group Ltd ("BMJ") its licensees and where the relevant Journal is co-owned by BMJ to the co-owners of the Journal, to publish the Work in this journal and any other BMJ products and to exploit all rights, as set out in our licence.

The Submitting Author accepts and understands that any supply made under these terms is made by BMJ to the Submitting Author unless you are acting as an employee on behalf of your employer or a postgraduate student of an affiliated institution which is paying any applicable article publishing charge ("APC") for Open Access articles. Where the Submitting Author wishes to make the Work available on an Open Access basis (and intends to pay the relevant APC), the terms of reuse of such Open Access shall be governed by a Creative Commons licence – details of these licences and which Creative Commons licence will apply to this Work are set out in our licence referred to above.

Other than as permitted in any relevant BMJ Author's Self Archiving Policies, I confirm this Work has not been accepted for publication elsewhere, is not being considered for publication elsewhere and does not duplicate material already published. I confirm all authors consent to publication of this Work and authorise the granting of this licence.

For peer review only

What are the volume and budget needs to provide chemotherapy to all children with acute lymphoblastic leukemia in Thailand? Development and application of an estimation tool

Authors: Rosarin Sruamsiri[1†], Alessandra Ferrario[2*†], Dennis Ross-Degnan[2], Avram E Denburg[3,4], A Lindsay Frazier[5], Sumit Gupta[3,4], Zachary J Ward[6], Jennifer M Yeh[7], Anita K Wagner[2]

[1]Center of Pharmaceutical Outcomes Research, Naresuan University, Phitsanulok, Thailand

[2]Department of Population Medicine, Harvard Medical School and Harvard Pilgrim Health Care Institute, Boston, USA

[3]Child Health Evaluative Sciences, Hospital for Sick Children, Toronto, Canada

[4]Division of Haematology/Oncology, Hospital for Sick Children, Toronto, Canada

[5]Dana-Farber/Boston Children's Cancer and Blood Disorders Center, Boston, USA

[6]Center for Health Decision Science, Harvard TH Chan School of Public Health, Boston, USA

[7]Department of Pediatrics, Harvard Medical School, Boston, USA; Division of General Pediatrics, Boston Children's Hospital, Boston, USA

[†]Joint first authors (RS and AF contributed equally to this paper)

*Corresponding author

Alessandra Ferrario
Division of Health Policy and Insurance Research
Department of Population Medicine
Harvard Medical School and Harvard Pilgrim Health Care Institute
401 Park Drive, Suite 401 East
Boston, MA 02215
Email: Alessandra_Ferrario@hphci.harvard.edu
Tel. +1 617 867 4863

Key words: leukemia; pediatric oncology; health policy

Word count and reference count: 3039 words and 44 references

For peer review only - http://bmjopen.bmj.com/site/about/guidelines.xhtml

Abstract *(300 words)*

Objective: Insufficient access to anti-cancer medicines may contribute to the wide survival differences of children with cancers across the globe. We developed a tool to estimate the volume of medicines and budget requirements to provide chemotherapy to children with acute lymphoblastic leukemia (ALL).

Design: Development and application of an estimation tool.

Setting: Pediatric oncology hospital departments in Thailand.

Participants: 318 children aged 0 to 14 years, diagnosed with ALL and 215 children with undiagnosed ALL.

Interventions: Estimates of volume and budget requirements for administering a full course of chemotherapy for ALL and a further course for children who relapse, according to national treatment guidelines.

Primary and secondary outcome measures: Primary outcome measures were the volume (mg) and cost (US$) of medicines needed to treat children with ALL. For medicines whose main indication is pediatric ALL (asparaginase and 6-mercaptopurine), we estimated the difference between volume needed and actual sales in 2017 (secondary outcome).

Results: Ten anti-cancer medicines and four chemoprotective agents are needed for the treatment of pediatric ALL according to the Thai treatment guidelines. Of these fourteen medicines, thirteen are included in the WHO essential medicines list for children. All are available as generics. We estimated that essential chemotherapy and chemoprotective agents to treat all children diagnosed with ALL in Thailand in 2017 would cost US$ 814 952 (US$ 1 365 422 for diagnosed and undiagnosed children), which corresponds to 0.005% (0.008%) of the country's total health expenditure. The volumes of asparaginase and 6-mercaptopurine available on the Thai market in 2017 were more than sufficient (2.3 and 1.5 times the amounts needed, respectively) to treat all children diagnosed with ALL.

Conclusions: Procuring sufficient quantities of essential medicines to treat children with ALL requires relatively modest resources. Medicine cost should not be a major barrier to ALL treatment in similar settings.

For peer review only

For peer review only - http://bmjopen.bmj.com/site/about/guidelines.xhtml

Strengths and limitations of this study

- The tool is dynamic and facilitates transparency for estimating the volume of medicines and budget requirements.
- It allows users to input local data of disease incidence, treatment protocols, and medicine prices.
- Our application uses data on cancer incidence, prices of medicines, and the volume of medicines available in Thailand.
- While informed by local data, we had to make some assumptions on cancer stage distributions.

For peer review only

For peer review only - http://bmjopen.bmj.com/site/about/guidelines.xhtml

Background

Important global and local forces have shaped the pediatric oncology landscape over the past two decades. The pediatric oncology community, together with researchers and other partners, has advocated widely for the need to address disparities in survival between countries at different levels of health system development (1–3). Global efforts to close this gap have featured the development of resource-appropriate treatment guidelines (4,5), the inclusion of key medicines to treat pediatric cancer in iterative updates of the World Health Organization's (WHO) Essential Medicines List for Children (6), the establishment of twinning programs (7,8) and global working groups like the WHO Global Initiative for Childhood Cancer (9), the development and strengthening of pediatric cancer registries and analytical efforts to provide evidence on the burden of pediatric cancer (10–14). These pediatric oncology focused endeavors have been supported by broader global commitments and efforts to achieve universal health coverage and the sustainable development agenda which, among other targets, emphasizes reduction in preventable mortality from non-communicable diseases and access to quality-assured essential medicines (15,16).

At the country level, efforts have included the development and implementation of standardized protocols to treat pediatric cancers, the establishment of national cancer plans and cancer registries, and the addition of pediatric cancer medicines to national essential medicines and reimbursement lists (17), among others. The extent to which different countries have engaged in such efforts varies. Thailand has been in the vanguard of these positive global trends. In Thailand, national treatment guidelines for childhood leukemia were developed in 2005 and implemented in 2006, together with coverage for diagnostics, chemotherapy and hospital treatment by the National Health Security Office (18–20). Pediatric cancer registries are in place across the country and regular analyses of incidence and survival have been published over time (18–22). At 66%, 5-year net survival of children with acute lymphoblastic leukemia (the most common form of cancer in children in most regions of the world) in Thailand is higher than the global (56%), Asian (53%), South-Eastern Asian (40%) and the upper middle-income countries averages (63%) but lower than survival in high income countries (88% on average) (13) (see Supplementary table 1 for more indicators).

A key component of pediatric cancer treatment is chemotherapy. Yet, despite calls for making medicines affordable and the need to address shortages affecting pediatric cancer medicines globally (1,23,24),

For peer review only - http://bmjopen.bmj.com/site/about/guidelines.xhtml

For peer review only

analyses of availability of pediatric cancer medicines at the country level are limited. Previous multi-country studies reviewed the inclusion of pediatric cancer medicines in national essential medicines and reimbursement lists (25) and surveyed physician and health care professionals regarding the level of access to essential pediatric chemotherapies (26). Subnational studies have assessed medicines availability and affordability at the pharmacy level (27). These are important contributions which need to be complemented by evidence on the actual volumes of medicines needed and available, and the corresponding budget required to provide a full course of treatment to all children with cancers. Further, longstanding supply chain vulnerabilities impacting access to quality medicines have been dramatically exposed during the COVID-19 pandemic (28,29), calling for even greater attention to availability of medicines.

Together with other health system barriers, insufficient access to anti-cancer medicines may contribute to the wide survival differences of children with cancers across the globe. The objective of our study was to develop a tool to estimate medicine needs and budgetary requirements for treating children with acute lymphoblastic leukemia (ALL) and apply it to Thailand as an example case. We populated the tool with a unique set of data including the latest available estimates of children with diagnosed and undiagnosed ALL, pharmaceutical sales data and local data on cancer stage distribution. Further, we compared needed quantities with sold quantities of pediatric cancer medicines used for the treatment of ALL to identify possible unmet need. Ultimately, we aimed to generate a transferable approach that can be customized to other jurisdictions and populated with local data to estimate the need for and availability of essential pediatric cancer medicines, as well as their corresponding budget impact. We expect this tool to be useful for procurement planning, budgeting, advocacy, and monitoring of sufficiency of medicine supplies.

Methods

Data sources

Cancer incidence by risk group or stage

We used age-stratified (<1; 1-4, 5-9, 10-14 years old) estimates of the number of children with incident ALL, diagnosed and undiagnosed, from the Global Childhood Cancer (GCC) microsimulation model (12). These GCC estimates were calibrated against the International Incidence of Childhood Cancer data (30) and included relevant demographic, incidence and health system variables to estimate the number of

For peer review only - http://bmjopen.bmj.com/site/about/guidelines.xhtml

diagnosed and undiagnosed cases (12). As published international estimates do not sub-classify patients by risk group, we estimated the proportion of children with ALL by risk group (standard, high, relapse) using data on the proportion of children per risk group treated with standard vs. high risk ALL protocols in Thailand (19).

Therapeutic guidelines

We used the national treatment guidelines published in 2016 by the Thai National Health Security Office (NHSO) (31) which were developed using treatment regimens implemented in advanced health care systems. NHSO guidelines are the basis for health insurance reimbursement for approximately 80% of the Thai population. The guidelines include separate protocols for standard risk ALL, high risk ALL and for relapsed ALL (the latter with separate treatment schemes for standard and high-risk relapse). All protocols specify recommended doses of each medication by age group, risk group, and treatment phase. We identified all antineoplastic agents and chemoprotective agents in the 2016 NHSO pediatric ALL treatment guidelines (31).

Medications sold

We estimated the volumes of each medication of interest sold in Thailand from January 1, 2017 through December 31, 2017 using IQVIA (formerly IMS Health and Quintiles) data. IQVIA is a multinational company that conducts multi-sample audits of pharmaceutical purchase data based on invoices from pharmacies, wholesalers, distributors, and manufacturers in the hospital and retail sectors in a number of countries worldwide (32). The proprietary data are extrapolated to represent national level sales. Quality checks (e.g. comparison with manufacturer data) are conducted to ensure accuracy and representativeness of the data (32). IQVIA sales data record quarterly sales volumes of each product with information on formulation, strength, number of units per pack and number of packs sold. We used this information to calculate the total volume, in milligrams (mg), of each active ingredient sold in 2017. We expressed volume of medicines sold as the number of smallest units for the relevant formulation (i.e. the one recommended in the Thai pediatric ALL guidelines) of each international nonproprietary name (INN). We defined the smallest unit as the unit with the smallest amount (mg or active units) of active ingredient for the relevant formulation listed in the 2019 WHO model list of essential medicines for children (6).

For peer review only - http://bmjopen.bmj.com/site/about/guidelines.xhtml

Prices

We extracted price data from the Pharmaceutical Information Center, Ministry of Public Health, Thailand (33). These represent list prices and may be higher than the prices actually paid. As such, they provide a conservative estimate of the maximum price paid.

Analyses

Pediatric ALL medication need

Building on earlier work (2014) by some of the authors (RS, DRG, AKW and ALF), we updated a tool that we created to forecast medicine need. We used data on weight and height of Thai children by age (34) to calculate the average body surface area for each age group according to the Mosteller formula (35). We multiplied the number of patients in each age and risk-group by the milligrams (or active units) per body surface area needed, for each ALL medication, according to the treatment protocols. We then summed the number of mg needed across different age and risk groups to arrive at a total number of milligrams for each antineoplastic and chemoprotective agent needed to treat both diagnosed and undiagnosed children with ALL in Thailand. We present the volume of ALL medicines needed as the number of smallest units.

Comparing volumes needed and volumes sold

Of the ten anticancer medicines included in the pediatric ALL protocols, most are also used for high-incidence adult cancers like breast cancer and lung cancer. Only two medicines, asparaginase and 6-mercaptopurine, are used predominantly for pediatric ALL, with a relatively small proportion of the total volume used for adolescents and young adults with ALL. In Thailand, asparaginase is a minor agent in treatment protocol for acute myeloid leukemia (in 2017 there were 97 children diagnosed with this type of cancer in Thailand). For these two medications, we compared the smallest units of both estimated needs and national sales volumes between January and December 2017.

Expenditure

Using price and volume data, we calculated the budget needed to purchase antineoplastic and chemoprotective agents for diagnosed only and diagnosed and undiagnosed children in 2017 and 2021.

For peer review only - http://bmjopen.bmj.com/site/about/guidelines.xhtml

Prices were adjusted for inflation (36). For the 2017 estimates, we calculated what percentage of the national health care expenditure (37) was required to pay for these medicines.

We conducted sensitivity analyses by varying the proportion of children who relapse and the protocol on which they relapse (standard vs. high risk) and by using the upper bound of the estimate of the number of children with ALL (12). Further, we tested the additional budgetary requirements if 20% and 40% of each vial content is wasted due to larger than needed vial size. Data preparation was conducted in Excel and the final analysis was conducted in Stata 15.1 (StataCorp LLC).

This study was approved by the Harvard Pilgrim Health Care Institute Institutional Review Board. The corresponding authors had full access to all data and the final responsibility to submit for publication. We used the STROBE reporting guidelines for observational studies in epidemiology (38) to guide the writing of this study.

Patient and public involvement

Patients or the public were not involved in the design, or conduct, or reporting, or dissemination plans of our research.

Results

Based on the Global Childhood Cancer microsimulation model, there were an estimated 318 (95% uncertainty interval 197-434) children 0-14 years old with diagnosed ALL and 215 children with undiagnosed ALL in Thailand in 2017, for a total to 533 (95% uncertainty interval 296-795) children with ALL (Table 1). Children who develop ALL from age 10-14 are considered high risk, so all children in this age group were allocated to the high-risk protocol. Based on published local evidence of stage distribution and relapse (19), 65% of 0-9 year old children were categorized as standard-risk and 35% as high-risk. Further, we estimated that 69 diagnosed children (22%) would relapse (Table 2). Of these relapses, we estimated that 28% would be treated on the standard-risk relapse protocol and 72% on the high-risk relapse protocol based on evidence from previous local studies on immunotype and site of relapse (19,21), risk allocation according to the national treatment guidelines (31) and assuming equal distribution of very early, early and late relapse.

For peer review only - http://bmjopen.bmj.com/site/about/guidelines.xhtml

For peer review only

<Table 1: Estimated numbers of Thai children aged 0-14 years with ALL in 2017 and 2021>

<Table 2: Estimated numbers of Thai children with ALL who relapse by risk-group in 2017 and 2021>

Ten anti-cancer medicines and four chemoprotective agents are employed in the treatment of pediatric ALL in Thailand. Thirteen of these fourteen medicines are included in the WHO essential medicines list for children and all medicines are available as generics. Providing these medicines to all diagnosed children would cost an estimated US$ 814,952 (US$ 1,365,422 including undiagnosed children), which represents 0.005% (0.008% including undiagnosed) of total health expenditure in Thailand in 2017 (Table 3). Five medicines would account for more than 90% of total expenditure (asparaginase 28%, methotrexate 26%, 6-mercaptopurine 20%, cytarabine 9% and vincristine 9%). For medicines used almost exclusively for the treatment of pediatric ALL, asparaginase and 6-mercaptopurine, the volume sold is more than sufficient (2.3 and 1.5 times, respectively, the amounts needed) to treat all children diagnosed with ALL, including children who relapse.

< Table 3: Medications for pediatric ALL – estimated volumes needed, cost, and actual sales in Thailand in 2017 and 2021>

Sensitivity analyses showed that even if the number of children with pediatric ALL is 27% higher (upper bound of 95% uncertainty interval), or if 50% of children relapse, or if the ratio of standard vs. high risk relapse is 60% greater, the total expenditure required to treat diagnosed and undiagnosed children would still be less than 0.02% of total health expenditure (see supplementary material). If 20% of the vial content (for all medicines that listed in the protocols to be administered in vials) is wasted, an additional US$ 123,439 would be required to treat all diagnosed children in 2017. The amount increases to US$ 246,876 if 40% of the volume in each vial is wasted.

Discussion

In this study, we contribute to global and local efforts to improve pediatric cancer treatment with the development of a tool to estimate the volume of medicines needed and available, and the corresponding budget required to implement national treatment protocols for pediatric ALL. We find that providing essential medicines to all children with ALL (diagnosed and undiagnosed) in Thailand would cost US$ 1,365,422 which corresponds to less than 0.01% of the country's 2017 health care

For peer review only - http://bmjopen.bmj.com/site/about/guidelines.xhtml

expenditure. To help inform procurement planning going forward, we also estimated volume and budgetary requirements for 2021 and found that accounting for inflation and changes in incidence, the expected budget impact of pediatric ALL antineoplastic and chemoprotective medications is US$ 1,421,880 (diagnosed and undiagnosed). Treating the most common form of pediatric cancer thus requires a relatively modest investment. The strengths of our tool are that it is based on local treatment protocols, local incidence estimates of both diagnosed and undiagnosed children, and local medicines prices and sales; the model can readily be customized for use by other countries who populate it with their own data.

Access to essential pediatric oncology medicines is a long-standing (1) and complex issue. Certain barriers to access disproportionately impact countries with developing health systems, such as limited affordability, inefficient procurement practices, supply chain weaknesses, and poor quality medicines (39). However, countries at all levels of health system development are affected by recurrent shortages of essential pediatric antineoplastic medicines (40). These shortages are due to a range of factors including thin profit margins for generic antineoplastics, interruptions in sole source production due to issues in the manufacture of sterile injectables, and poor forecasting of need (41). Shortages introduce risks of sub-optimal treatment due to treatment interruptions, substitution, and increased risk of exposure to substandard or falsified medicines procured reactively through available alternative suppliers (42,43). Availability challenges have been amplified during the COVID-19 pandemic. It is therefore very timely to reinforce the importance of estimating needed procurement volumes and budgets (44). The tool we developed can support country planners.

A national essential medicines list linked with standard treatment guidelines and universal health coverage have been identified as keys to improved access to pediatric cancer treatment and ultimately health outcomes. Given the imperative of using scarce resources wisely on the path to universal health coverage, it is important to know the extent of treatment needs and expected budget impacts. Accurate forecasts of need can help increase procurement efficiency. If procurement is aggregated across different hospitals, it can generate economies of scale (44). Knowledge of the types of medicines needed and volumes required can enable measures to proactively prevent or mitigate the impact of anticipated or recurrent shortages.

For peer review only - http://bmjopen.bmj.com/site/about/guidelines.xhtml

There are limitations to our study. Our model includes some assumptions. We assume that all children complete the full first course of treatment and that those who relapse complete the full relapse protocol. We made these assumptions because we wanted to estimate the volume and budget that would be needed if every child received a full treatment course. In reality, some children will relapse, die from ALL or treatment toxicity, or abandon therapy during their treatment course. The Thai guidelines include separate treatment regimens for low risk infants and intermediate/high risk infants. Given that less than 4% of children with ALL in Thailand were infants (<1 year old) we included them in the 0-4 year old group, which was assigned proportions of 0.65 and 0.35 to either the standard or high risk protocols, respectively. The distributions of risk status and assigned treatment protocol were based on assumptions informed by local data. Sensitivity analyses varying these distributions did not change our overall conclusions. The IQVIA sales data do not capture medications obtained through special procurement channels, such as donated products. We may therefore have underestimated the available volumes of some products. However, as we found no gaps in availability, this would not affect our finding of sufficient amounts of asparaginase and 6-mercaptopurine in Thailand at the time of the study. Finally, while availability of medicines is an essential component of effective pediatric cancer treatment, improving outcomes of care also requires other health systems inputs (e.g. pediatric oncologists and nurses) and processes (e.g. diagnostic capacity, referrals, access to specialist care, ability to prevent and treat side effects). Global childhood cancer 5-year survival rates are projected to improve from 37% to ≥50% with expanded treatment access or improved service delivery, and to 42% with improved access to chemotherapy only (13). Equitable access to appropriate medicines is thus a necessary but not sufficient component of a health system equipped to treat children with cancers.

Conclusions

The survival disparities among children with cancer between high- and low- and middle-income countries have been recognized as an unacceptable injustice which demands redress. Medicines are an important component in the set of interventions needed to bridge the gaps in global 5-year net survival rates which range from 12% in Eastern Africa, 40% in South-East Asia, to 90% in Western and Northern Europe, North America, Australia and New Zealand (13). The introduction and expansion of universal health coverage in low- and middle-income countries provides an opportunity to save lives by including pediatric cancer medicines in benefit packages. To do so requires sound, context-specific estimates of needed volumes and expected costs of pediatric cancer medications.

For peer review only - http://bmjopen.bmj.com/site/about/guidelines.xhtml

We developed a practical, customizable tool to transparently generate such estimates based on the needs and circumstances of different countries. Estimates can be used for advocacy and to inform procurement processes with the aim of increasing efficiency. Ultimately, evidence-based estimates of needs, together with market analysis, can help countries move from passive purchasing based on historical data to strategic procurement and proactive mitigation of potential medicine shortages.

Acknowledgments

This work would not have been possible without the numerous contributions from Dr. Peter Stephens from IQVIA, United Kingdom. We gratefully acknowledge Dr. Stephens and IQVIA for providing the sales data and information on medicines sales data. We gratefully acknowledge the contribution of Prof. Dr. Suradej Hongeng, Department of Pediatrics, Faculty of Medicine, Ramathibodi Hospital, Mahidol University, Bangkok, Thailand in providing important information on the treatment of pediatric ALL in Thailand. We gratefully acknowledge the Global Task Force on Expanded Access to Cancer Care and Control, led by Dr. Felicia Knaul, which provided funding for the initial development in 2014 of the approach to estimate gaps between needs for and availability of medications to treat children with cancers.

Contributors

RS and AF contributed equally to this paper. RS, DRG and AW with contribution from ALF developed a tool to estimate need and availability as part of the POEMS project in 2014. The tool was updated and enhanced between 2019 and 2020 by AF and RS who also compiled the data and conducted the analysis. SG, AD and ALF advised on clinical aspects. JY and ZW advised on analytical aspects. AF wrote the first draft of the paper; all authors contributed to revisions of the manuscript to its final version and approved the final version.

Funding

The initial development of a tool (POEMS project 2014) on which this work built was funded by the Global Task Force for Cancer Care and Control (grant number N/A), an initiative promoted by the Harvard Global Equity Initiative, Harvard Medical School, Harvard T.H. Chan School of Public Health,

For peer review only - http://bmjopen.bmj.com/site/about/guidelines.xhtml

Dana-Farber Cancer Institute, Fred Hutchinson Cancer Research Center, and University of Washington School of Medicine. At that time, RS was a Research Fellow in the Department of Population Medicine and supported by the Thai Royal Golden Jubilee PhD program (grant number PHD/0127/2552). There were no external funds provided for the present analyses. AF was supported by a postdoctoral Fellowship from the Swiss National Science Foundation (grant number P400PM_183877) and AKW by a Department of Population Medicine Ebert Award (grant number N/A), Harvard Medical School and Harvard Pilgrim Health Care Institute. The funders of this study had no role in study design, collection, analysis, or interpretation of the data, or writing of the report.

Competing interests

RS is currently employed by Takeda (Thailand) Ltd. AF reports personal fees from the European Society of Medical Oncology, separate from the submitted work. AKW reports support from the Global Task Force for Cancer Care and Control for the initial development of the tool. The other authors declare no competing interests.

Patient and public involvement

Patients or the public were not involved in the design, or conduct, or reporting, or dissemination plans of our research.

Ethics approval

This study was approved by the Harvard Pilgrim Health Care Institute Institutional Review Board.

Data availability statement

All data relevant to the study are included in the article or uploaded as supplementary information.

For peer review only

For peer review only - http://bmjopen.bmj.com/site/about/guidelines.xhtml

Tables

Table 1: Estimated numbers of Thai children aged 0-14 years with ALL in 2017 and 2021

	Diagnosed								Diagnosed and undiagnosed							
	Standard risk				High risk				Standard risk				High risk			
	n	LB	UB	%	n	LB	UB	%	n	LB	UB	%	n	LB	UB	%
**2017**																
Age group																
0-4	95	58	128	29.8	51	32	70	16.1	159	87	238	29.8	86	47	129	16.1
5-9	68	43	92	21.3	37	23	50	11.5	113	64	169	21.3	61	34	92	11.5
10-14	0	0	0	0	68	41	94	21.3	0	0	0	0.0	113	64	167	21.2
Total by risk group	162	101	221	51.1	155	96	213	48.9	272	151	408	51.1	261	145	387	48.9
Total by diagnosis status	318	197	434	100					533	296	795	100				
**2021**																
0-4	86	51	120	29.0	47	28	65	15.7	144	78	219	29.0	78	42	118	15.7
5-9	64	42	90	21.6	35	22	48	11.7	108	62	163	21.8	59	33	88	11.8
10-14	0	0	0	0.0	65	40	91	21.9	0	0	0	0.0	108	59	161	21.7
Total by risk group	150	93	210	50.7	146	90	204	49.3	252	140	382	50.8	244	134	367	49.2
Total by diagnosis status	297	183	414	100					496	274	749	100				

Notes: LB: lower bound; UB: upper bound of 95% uncertainty interval (calculated as the 2.5 and 97.5 percentiles). Numbers (n, LB, UB) were rounded to the nearest full digit.

For peer review only

For peer review only - http://bmjopen.bmj.com/site/about/guidelines.xhtml

Sources: Number of incident pediatric ALL cases (diagnosed, all) by age group (12), risk group proportions for children (19).

Table 2: Estimated numbers of Thai children with ALL who relapse by risk-group in 2017 and 2021

	Diagnosed								Diagnosed and undiagnosed							
	Standard risk				High risk				Standard risk				High risk			
	n	LB	UB	%	n	LB	UB	%	n	LB	UB	%	n	LB	UB	%
**2017**																
Total relapse by risk group*	19	12	26	28.0	50	31	68	72.0	32	18	48	28.0	83	46	124	72.0
Total relapse by diagnosis status	69	43	94	100					116	64	172	100				
**2021**																
Total relapse by risk group*	18	11	25	28.0	46	29	65	72.0	30	17	46	28.0	78	43	117	72.0
Total relapse by diagnosis status	64	40	90	100					108	59	162	100				

Notes: LB: lower bound; UB: upper bound of 95% uncertainty interval (calculated as the 2.5 and 97.5 percentiles). Risk classification for relapse treatment is independent of the original risk classification for the first full course of treatment. There is a second risk assessment for children who relapse. Numbers (n, LB, UB) were rounded to the nearest full digit.

Sources: Number of incident pediatric ALL cases (diagnosed, all) by age group (12), risk group proportions for children (19,21).

For peer review only

For peer review only - http://bmjopen.bmj.com/site/about/guidelines.xhtml

Table 3: Medications for pediatric ALL – estimated volumes needed, cost, and actual sales in Thailand in 2017 and 2021

			2017					2021			
			Total smallest units		Expenditure (US$)			Total smallest units		Expenditure (US$)	
	Route	Smallest unit	DX	DX + uDX	DX	DX + uDX	Percentage	DX	DX + uDX	DX	DX + uDX
Asparaginase	IM	10000	4,607 [10,365]	7,719	225,847	378,394	27.7	4,348	7,263	236,209	394,571
Methotrexate	*all*				213,031	356,925	26.1			222,654	371,993
Methotrexate	IV	50	83,180	139,364	*190,358*	*318,934*		78,482	131,109	*199,042*	*332,511*
Methotrexate	oral	2.5	235,036	393,798	*19,285*	*32,311*		221,110	369,701	*20,105*	*33,617*
Methotrexate	IT	10	6,730	11,281	*3,388*	*5,680*		6,285	10,512	*3507*	*5865*
Mercaptopurine	oral	50	223,501 [340,035]	374,472	164,313	275,303	20.2	210,281	351,584	171,324	286,448
Cytarabine	IV	100	18,905	31,674	73,572	123,268	9.0	17,798	29,752	76,763	128,319
Vincristine	IV	1	17,363	29,092	70,469	118,070	8.6	16,341	27,320	73,507	122,891
Tioguanine	oral	40	5,429	9,096	12,011	20,125	1.5	5,107	8,539	12,522	20,938
Mitoxantrone	IV	10	112	188	11,586	19,413	1.4	105	176	12,081	20,199
Doxorubicin	IV	10	3,401	5,698	9,705	16,260	1.2	3,211	5,363	10,155	16,961
Cyclophosphamide	IV	500	1,386	2,322	6,551	10,976	0.8	1,309	2,186	6,854	11,448
Etoposide	IV	100	375	628	2,112	3,538	0.3		589	2,202	3,681
Leucovorin	IV	30	3,438	5,761	21,030	35,236	2.6	3,235	5,409	21,928	36,663
MESNA	IV	400	822	1,378	2,565	4,298	0.3	780	1,301	2,401	4,004
Prednisolone	oral	5	183,024	306,654	1,863	3,122	0.2	172,180	287,889	1,943	3,249
Dexamethasone	oral	2	21,048	35,265	295	494	0.0	19,801	33,107	307	514
Antineoplastic agents					789,198	1,322,272				824,270	1,377,449
Chemoprotective agents					25,754	43,149				26,579	44,430

For peer review only
For peer review only - http://bmjopen.bmj.com/site/about/guidelines.xhtml

Total expenditure on antineoplastic and chemoprotective agents	814,952	1,365,422	850,849	1,421,880
Total health expenditure (in million constant (2017) US$)	17,055			
Percentage of total health budget	0.005	0.008		

Notes: Route of administration: IM: intramuscular; IV: intravenous; IT: intrathecal; mg: milligrams; [volume sold] is provided for medicines used mainly for the treatment of pediatric ALL. DX: diagnosed; DX + uDX: diagnosed and undiagnosed. Tioguanine is also known as thioguanine.

Source: Authors' estimation based on Thai ALL treatment protocol (31), incidence data (12), IQVIA sales data, WHO Global Health Expenditure Database (37).

For peer review only

For peer review only - http://bmjopen.bmj.com/site/about/guidelines.xhtml

Supplementary material

Key indicators in Thailand in comparison to regional, upper middle income and global averages

Incidence by age and risk group

ALL treatment protocol

Body surface area

Price of medicines

Sensitivity analysis

For peer review only

For peer review only - http://bmjopen.bmj.com/site/about/guidelines.xhtml

References

1. Pui C-H, Schrappe M, Masera G, Nachman J, Gadner H, Eden OB, et al. Ponte di Legno Working Group: statement on the right of children with leukemia to have full access to essential treatment and report on the Sixth International Childhood Acute Lymphoblastic Leukemia Workshop. Leukemia. 2004 Jun;18(6):1043–53.

2. Magrath I, Steliarova-Foucher E, Epelman S, Ribeiro RC, Harif M, Li C-K, et al. Paediatric cancer in low-income and middle-income countries. Lancet Oncol. 2013 Mar;14(3):e104-116.

3. Atun R, Bhakta N, Denburg A, Frazier AL, Friedrich P, Gupta S, et al. Sustainable care for children with cancer: a Lancet Oncology Commission. Lancet Oncol. 2020 Apr 1;21(4):e185–224.

4. Hunger SP, Sung L, Howard SC. Treatment strategies and regimens of graduated intensity for childhood acute lymphoblastic leukemia in low-income countries: A proposal. Pediatr Blood Cancer. 2009 May;52(5):559–65.

5. Yeoh AEJ, Tan D, Li C-K, Hori H, Tse E, Pui C-H, et al. Management of adult and paediatric acute lymphoblastic leukaemia in Asia: resource-stratified guidelines from the Asian Oncology Summit 2013. Lancet Oncol. 2013 Nov;14(12):e508-523.

6. WHO Model Lists of Essential Medicines [Internet]. World Health Organization. World Health Organization; [cited 2020 Mar 25]. Available from: http://www.who.int/medicines/publications/essentialmedicines/en/

7. Rodriguez-Galindo C, Friedrich P, Alcasabas P, Antillon F, Banavali S, Castillo L, et al. Toward the Cure of All Children With Cancer Through Collaborative Efforts: Pediatric Oncology As a Global Challenge. J Clin Oncol Off J Am Soc Clin Oncol. 2015 Sep 20;33(27):3065–73.

8. Hopkins J, Burns E, Eden T. International twinning partnerships: An effective method of improving diagnosis, treatment and care for children with cancer in low-middle income countries. J Cancer Policy. 2013 Mar 1;1(1):e8–19.

9. Global Initiative for Childhood Cancer [Internet]. World Health Organization. World Health Organization; [cited 2020 Mar 25]. Available from: http://www.who.int/cancer/childhood-cancer/en/

10. Bhakta N, Force LM, Allemani C, Atun R, Bray F, Coleman MP, et al. Childhood cancer burden: a review of global estimates. Lancet Oncol. 2019 Jan 1;20(1):e42–53.

11. Global Burden of Disease Cancer Collaboration, Fitzmaurice C, Allen C, Barber RM, Barregard L, Bhutta ZA, et al. Global, Regional, and National Cancer Incidence, Mortality, Years of Life Lost, Years Lived With Disability, and Disability-Adjusted Life-years for 32 Cancer Groups, 1990 to 2015: A Systematic Analysis for the Global Burden of Disease Study. JAMA Oncol. 2017 Apr 1;3(4):524–48.

12. Ward ZJ, Yeh JM, Bhakta N, Frazier AL, Atun R. Estimating the total incidence of global childhood cancer: a simulation-based analysis. Lancet Oncol. 2019 Apr;20(4):483–93.

For peer review only - http://bmjopen.bmj.com/site/about/guidelines.xhtml

13. Ward ZJ, Yeh JM, Bhakta N, Frazier AL, Girardi F, Atun R. Global childhood cancer survival estimates and priority-setting: a simulation-based analysis. Lancet Oncol. 2019 Jul 1;20(7):972–83.

14. Miranda-Filho A, Piñeros M, Ferlay J, Soerjomataram I, Monnereau A, Bray F. Epidemiological patterns of leukaemia in 184 countries: a population-based study. Lancet Haematol. 2018 Jan 1;5(1):e14–24.

15. United Nations. Sustainable Development Goals [Internet]. [cited 2019 Jan 12]. Available from: https://www.un.org/sustainabledevelopment/

16. Political Declaration of the High-level Meeting on Universal Health Coverage "Universal health coverage: moving together to build a healthier world" [Internet]. United Nations General Assembly; 2019 [cited 2020 Mar 26]. Available from: https://www.un.org/pga/73/wp-content/uploads/sites/53/2019/07/FINAL-draft-UHC-Political-Declaration.pdf

17. Chivukula MV, Tisocki K. Essential cancer medicines in the national lists of countries of the WHO South-East Asia Region: a descriptive assessment. WHO South-East Asia J Public Health. 2018;7(2):90–8.

18. Kanjanapongkul S. Outcome of acute lymphoblastic leukemia treatment using national protocols at the Queen Sirikit National Institute of Child Health. J Med Assoc Thai. 2014 Jun;97 Suppl 6:S1-5.

19. Seksarn P, Wiangnon S, Veerakul G, Chotsampancharoen T, Kanjanapongkul S, Chainansamit S-O. Outcome of Childhood Acute Lymphoblastic Leukemia Treated Using the Thai National Protocols. Asian Pac J Cancer Prev. 2015;16(11):4609–14.

20. Tharnprisan P, Khiewyoo J, Sripraya P, Wiangnon S. Relapse-free rate with childhood acute lymphoblastic leukemia treated under the thai national protocol. Asian Pac J Cancer Prev APJCP. 2013;14(2):1127–30.

21. Surapolchai P, Anurathapan U, Sermcheep A, Pakakasama S, Sirachainan N, Songdej D, et al. Long-Term Outcomes of Modified St Jude Children's Research Hospital Total Therapy XIIIB and XV Protocols for Thai Children With Acute Lymphoblastic Leukemia. Clin Lymphoma Myeloma Leuk. 2019 Aug;19(8):497–505.

22. Wiangnon S, Jetsrisuparb A, Komvilaisak P, Suwanrungruang K. Childhood cancer incidence and survival 1985-2009, Khon Kaen, Thailand. Asian Pac J Cancer Prev APJCP. 2014;15(18):7989–93.

23. Denburg A, Arora B, Arora RS, Auste C, Bagai P, Barr R, et al. Access to essential medicines for children with cancer: a joint SIOP-CCI position statement. Lancet Oncol. 2017 Jan;18(1):20–2.

24. Mehta PS, Wiernikowski JT, Petrilli JAS, Barr RD, Working Group on Essential Medicines of the Pediatric Oncology in Developing Countries committee of SIOP. Essential medicines for pediatric oncology in developing countries. Pediatr Blood Cancer. 2013 May;60(5):889–91.

25. Barr R, Robertson J. Access to Cytotoxic Medicines by Children With Cancer: A Focus on Low and Middle Income Countries. Pediatr Blood Cancer. 2016 Feb;63(2):287–91.

For peer review only - http://bmjopen.bmj.com/site/about/guidelines.xhtml

26. Cohen P, Friedrich P, Lam C, Jeha S, Metzger M, Qaddoumi I, et al. Global Access to Essential Medicines for Childhood Cancer: A Cross-Sectional Survey. - PubMed - NCBI. 2018;4(Dec):1–11.

27. Faruqui N, Martiniuk A, Sharma A, Sharma C, Rathore B, Arora RS, et al. Evaluating access to essential medicines for treating childhood cancers: a medicines availability, price and affordability study in New Delhi, India. BMJ Glob Health [Internet]. 2019 Apr 23 [cited 2020 Mar 26];4(2). Available from: https://www.ncbi.nlm.nih.gov/pmc/articles/PMC6509613/

28. USP. USP Global Public Policy Position. Key Elements to Building a More Resilient Supply Chain [Internet]. The United States Pharmacopeia; [cited 2020 May 28]. Available from: https://www.usp.org/sites/default/files/usp/document/our-impact/covid-19/global-policy-supply-chain.pdf

29. GlobalData Healthcare. Governments can avoid potential cancer drug shortages amid Covid-19 pandemic [Internet]. 2020 [cited 2020 May 31]. Available from: https://www.pharmaceutical-technology.com/comment/cancer-drug-shortages-covid-19/

30. Steliarova-Foucher E, Colombet M, Ries L, Hesseling P, Moreno F, Shin H, et al. International Incidence of Childhood Cancer, volume III (electronic version) [Internet]. Lyon, France: International Agency for Research on Cancer; 2017 [cited 2020 Feb 25]. Available from: http://iicc.iarc.fr/results

31. Cancer Treatment Guidelines - National protocol for the treatment of childhood cancers 2016 [Internet]. The Thai Pediatric Oncology Group; The Thai Society of Hematology; National Health Security Office; [cited 2019 Aug 9]. Available from: https://www.nhso.go.th/FrontEnd/page-information_detail.aspx?ContentID=NjAwMDAwMjY5

32. IQVIA Quality Assurance [Internet]. Frankfurt am Main: IQVIA; [cited 2020 Feb 14]. (acts 2018). Available from: https://www.iqvia.com/-/media/iqvia/pdfs/library/publications/acts-2018-32nd-edition.pdf?_=1581584888234

33. Drug and Medical Supply Information Center, Ministry of Public Health. Average medicine prices [Internet]. [cited 2020 May 20]. Available from: http://dmsic.moph.go.th/

34. [Report on the Thai population Health: 2000. Ministry of Public Health] (in Thai) [Internet]. [cited 2020 May 9]. Available from: https://www.hiso.or.th/hiso/picture/reportHealth/report/report6.pdf

35. Mosteller R. Simplified Calculation of Body-Surface Area. N Engl J Med. 1987 Oct 22;317(17):1098–1098.

36. Thailand: Inflation rate from 2004 to 2021 [Internet]. Statista. Available from: https://www.statista.com/statistics/332274/inflation-rate-in-thailand/

37. Global Health Expenditure Database [Internet]. World Health Organization. [cited 2020 Apr 10]. Available from: https://apps.who.int/nha/database

For peer review only - http://bmjopen.bmj.com/site/about/guidelines.xhtml

38. EQUATOR Network. The Strengthening the Reporting of Observational Studies in Epidemiology (STROBE) Statement: guidelines for reporting observational studies [Internet]. [cited 2020 Jun 19]. Available from: https://www.equator-network.org/reporting-guidelines/strobe/

39. Silverman R, Keller J, Glassman A, Chalkidou K. Tackling the Triple Transition in Global Health Procurement [Internet]. Washington, DC: Center for Global Development; 2019 [cited 2020 May 28]. Available from: https://www.cgdev.org/better-health-procurement

40. Unguru Y, Bernhardt MB, Berg SL, Johnson L-M, Pyke-Grimm K, Woodman C, et al. Chemotherapy and Supportive Care Agents as Essential Medicines for Children With Cancer. JAMA Pediatr. 2019 01;173(5):477–84.

41. WHO Executive Board. Addressing the global shortages of medicines, and the safety and accessibility of children's medication [Internet]. Geneva: World Health Organization; 2015 Dec [cited 2020 Jun 1]. (138th session). Available from: https://apps.who.int/iris/bitstream/handle/10665/250709/B138_41-en.pdf;jsessionid=7060D7B9E132A783D8027DAD372DC812?sequence=1

42. Dandan N. Shortage of Critical Leukemia Drug Threatens Ill Children [Internet]. 2017 [cited 2020 May 20]. Available from: https://www.sixthtone.com/news/1000776/shortage-of-critical-leukemia-drug-threatens-ill-children

43. Pisani E, Nistor A, Hasnida A, Parmaksiz K, Xu J, Kok M. Identifying market risk for substandard and falsified medicines: an analytic framework based on qualitative research in China, Indonesia, Turkey and Romania. Wellcome Open Research [Internet]. 4(70). Available from: https://wellcomeopenresearch.org/articles/4-70/v1

44. Ferrario A, Kanavos PG, Humbert T, Iwamoto K, Bak Pedersen H. Challenges and opportunities in improving access to medicines through efficient public procurement in the WHO European Region. Copenhagen: World Health Organization Regional Office for Europe; 2016.

For peer review only

For peer review only - http://bmjopen.bmj.com/site/about/guidelines.xhtml

Key indicators in Thailand in comparison to regional, upper middle income and global ave

Indicator
Population (million)[1], 2018
Percentage of the population ages 0-14[1], 2018
GDP per capita, PPP (current international $)[1], 2018
Income share held by highest 20%[1], 2018
Current health expenditure per capita, PPP (current international $)[1], 2017
omestic general government health expenditure (as percentage of current health expenditure)[1], 201
Year UHC started to be implemented
Five-year net survival ALL, 2015-2019[3]

Notes: *Comparison are made where relevant

Sources: [1]World Bank Open Data (https://data.worldbank.org/, accessed 13 April 2020); [2]Global Hea

For peer review only

erages*

Thailand	East Asia & Pacific (East Asia & Pacific excluding high income)	Upper middle income	Global
69	-	-	-
17%	20% (20%)	21%	26%
19,051.3	19,329.8 (16,113.5)	19,028.9	17,948.4
44.00%	-	-	-
670.88	975.21 (647.38)	964.5	1,355.60
76.10%	68.2% (56.8%)	54.60%	74.30%
2001	-	-	-
66.50%	52.6% (Asia) 39.7% (South-East Asia)	63.20%	56.10%

alth Expenditure Database (WHO); [3](Ward et al. Lancet Oncology 2019)

For peer review only

Calculation of number of children with ALL by risk group

1) Calculation of the number of children with ALL

Mean estimate

2017	<1	1-4	5-9	10-15
Diagnosed	11	135	104	68
Diagnosed and undiagnosed	19	226	175	113

2021	<1	1-4	5-9	10-15
Diagnosed	10	123	99	65
Diagnosed and undiagnosed	17	205	167	108

Notes: 95% uncertainty interval calculated as the 2.5 and 97.5 percentiles
Source: Ward et al. Lancet Oncology, 2019 https://doi.org/10.7910/DVN/CTJN63

2) Calculatation of children by risk group

All children age 10-15 are classified as high risk. Question: what is the proportion of children age
1. Derive proportion of Thai children with ALL who are 0-9 vs. 10-15

Proportion 0-9	0.79
Proportion 10-15	0.21

2. Based on the above proportion, estimate the number of children in the Seksarn et al. 2015 pa

Number 0-9	376
Number 10-15	102

3. Given that all 10-19 year olds are high risk and that 234 patients in the Seksarn paper were h

Number low risk in Seskarn (all 0-9 years old - 0-9 years
Number high risk in Seskarn (all 10-15 years old children

4. Therefore we can now estimate the proportion of 0-9 year olds that are high risk:

5. Thus, in the modeling, all 10-19 year olds are assigned to the high risk treatment. O assigned to standard risk treatment

2017 DIAGNOSED

Age group	1	2	3
	**0-4**	**5-9**	**10-14**
**Standard risk**	95	68	0
**High risk**	51	37	68

2021

	1	2	3
	**0-4**	**5-9**	**10-14**
**Standard risk**	86	64	0
**High risk**	47	35	65

Calculation of number of children who relapse by risk group

1. According to (Bunyatisai et al. 2019; Seksarn et al. 2015) on average 22% of childr

2. Estimate the proportion of children who relapse on the standard risk protocol vs. hi[illegible]

	Surapolchai et al. 2019 (2	ksarn et al. 20	rtion across studies
a) Immunotype			
-pre-B lineage/ B cell	138	309	0.87
-T-cell lineage	14	51	0.13
Relapse	15	95	
b) Site of relapse			
Isolated extramedullary	0.333	0.305	0.31
- Central nervous system (CNS)	4	28	
- Testis	1	1	
Isolated marrow	0.467	0.526	0.52
- Bone marrow (BM)	7	50	*
Combined	0.143	0.168	0.17
- CNS+BM	2	15	
- BM + testis		1	
Other relapse sites	1		

2017	DIAGNOSED		
Age group	1	2	3
	**0-4**	**5-9**	**10-14**
**Total number of relapses**	32	23	15
**Standard risk**	9	6	4
**High risk**	23	16	11

2021	DIAGNOSED		
Age group	1	2	3
	**0-4**	**5-9**	**10-14**
**Total number of relapses**	29	21	14
**Standard risk**	8	6	4
**High risk**	21	15	10

Note: The risk group to which children who relapse are allocated is independent of the risk grou[illegible]

Data sources	Accessed	
Ward et al. 2019	5-Jun-20	https://doi.org/10.7910/DVN/CTJN63
Seksarn et al. 2015	5-Jun-20	http://dx.doi.org/10.7314/APJCP.2015.16.[illegible]

For peer review only

Bunyatisai et al. 2019	5-Jun-20	https://he01.tci-thaijo.org/index.php/jtaro/
Thai ALL protocols 2016	5-Jun-20	https://www.nhso.go.th/FrontEnd/page-inf

For peer review only

Total
318
533

Lower bound of 95% uncertainty interval (calculated as the 2.5 ar

2017	<1	1-4	5-9
Diagnosed	5	85	66
Diagnosed and	7	127	98

Total
297
496

2021	<1	1-4	5-9
Diagnosed	4	75	64
Diagnosed and	7	113	95

ed 0-9 who are high risk?

aper (N=478) who are 0-9 vs. 10-15

Source: Seksarn et al. Asian

nigh risk, estimate that (234-102)=132 0-9 year olds were high risk

old high risk) 244

+ 35% of 0-9 years old) 234

0.35

)f the 0-9 year olds, 35% are assigned to high risk treatment, while 65% are

DIAGNOSED AND NOT DIAGNOSED

Age group	1	2	3
	**0-4**	**5-9**	**10-14**
**tandard risk**	159	113	0
**High risk**	86	61	113

	1	2	3
	**0-4**	**5-9**	**10-14**
**tandard risk**	144	108	0
**High risk**	78	59	108

Scenario 0

	Proportion relapse		
		Basecase (=0	0.22
**en with ALL relapse**	0.22	Sensitivity an	0.5

For peer review only

gh risk protocol according to the risk allocation table for relapse (Thai ALL pr

3. Allocate different sites of relapse to either standard or high ris
Variables for risk allocation (relapse)
a) Immunophenotype: use proportions from the literature (these refer t
b) Site of relapse: use data from the literature to calculate proportions
c) Time of relapse: Assume equal distribution across time of relapse (1/
d) Intermediate risk: Assume equal allocation to SR and HR protocol (1/
Protocol: assume that among intermediate risk children, 1/2 receive SR

Immunophenotyion	immunophe	Site of relapse	rtion site of re	Very early<br>months from 1s
		Isolated extra	0.3	High
Non T-cell	0.87	Isolated marr	0.5	High
		Combined	0.2	High
		Isolated extra	0.3	High
T-cell	0.13	Isolated marr	0.5	High
		Combined	0.2	High

Source risk allocation table: p.55 Thai protocols

Proportion SR r
Proportion HR r

DIAGNOSED AND NOT DIAGNOSED

Age group	1<br>**0-4**	2<br>**5-9**	3<br>**10-14**
	53	38	25
	15	11	7
	38	27	18

DIAGNOSED AND NOT DIAGNOSED

Age group	1<br>**0-4**	2<br>**5-9**	3<br>**10-14**
	48	36	23
	13	10	7
	35	26	17

p for the baseline treatment

11.4609

For peer review only

/article/view/203011
formation_detail.aspx?ContentID=NjAwMDAwMjY5

For peer review only

nd 97.5 percentiles)

10-15	Total
41	197
64	296

10-15	Total
40	183
59	274

Upper bound of 95% uncertainty interval (calculated as the 2.!

2017	<1	1-4	5-9
Diagnosed	20	178	142
Diagnosed an	35	332	261

2021	<1	1-4	5-9
Diagnosed	19	166	138
Diagnosed an	31	306	251

Pac J Cancer Prev 2015

For peer review only

otocols 2016)

sk relapse treatment protocol

:o first DX), assumption: the same proportion applied to relapse

'3 each)
/2 each)
. and 1/2 HR protocol

portion very e	Early	roportion earl	Late	Proportion late
0.33		0.33		0.33
st after 1st DX	and <6 months fr	≥6 months after stopping therapy		
0.09	Intermediate	0.09	Standard	0.09
0.15	High	0.15	Intermediate	0.15
0.05	Intermediate	0.05	Intermediate	0.05
0.01	Intermediate	0.01	Standard	0.01
0.02	High	0.02	High	0.02
0.01	High	0.01	High	0.01

0.28
0.72

Scenario		0
Basecase (=0)		
	SR	0.28
	HR	0.72
Sensitivity analysis (=1)		
	SR	0.5
	HR	0.5

For peer review only

For peer review only

5 and 97.5 percentiles)

10-15	Total
94	434
167	795

10-15	Total
91	414
161	749

For peer review only

	Age group (years)	Age group (#)	Risk group description	Risk group (#)	Treatment phase
pedi_ALL	0-4	1	Standard risk	1	Induction
pedi_ALL	0-4	1	Standard risk	1	Induction
pedi_ALL	0-4	1	Standard risk	1	Induction
pedi_ALL	0-4	1	Standard risk	1	Induction
pedi_ALL	0-4	1	Standard risk	1	Consolidation
pedi_ALL	0-4	1	Standard risk	1	Consolidation
pedi_ALL	0-4	1	Standard risk	1	Consolidation
pedi_ALL	0-4	1	Standard risk	1	Interim Maintenance
pedi_ALL	0-4	1	Standard risk	1	Interim Maintenance
pedi_ALL	0-4	1	Standard risk	1	Interim Maintenance
pedi_ALL	0-4	1	Standard risk	1	Interim Maintenance
pedi_ALL	0-4	1	Standard risk	1	Delayed Intensification
pedi_ALL	0-4	1	Standard risk	1	Delayed Intensification
pedi_ALL	0-4	1	Standard risk	1	Delayed Intensification
pedi_ALL	0-4	1	Standard risk	1	Delayed Intensification
pedi_ALL	0-4	1	Standard risk	1	Delayed Intensification
pedi_ALL	0-4	1	Standard risk	1	Delayed Intensification
pedi_ALL	0-4	1	Standard risk	1	Delayed Intensification
pedi_ALL	0-4	1	Standard risk	1	Delayed Intensification
pedi_ALL	0-4	1	Standard risk	1	Maintenance
pedi_ALL	0-4	1	Standard risk	1	Maintenance
pedi_ALL	0-4	1	Standard risk	1	Maintenance
pedi_ALL	0-4	1	Standard risk	1	Maintenance
pedi_ALL	0-4	1	Standard risk	1	Maintenance
pedi_ALL	0-4	1	High risk	2	Induction
pedi_ALL	0-4	1	High risk	2	Induction
pedi_ALL	0-4	1	High risk	2	Induction
pedi_ALL	0-4	1	High risk	2	Induction
pedi_ALL	0-4	1	High risk	2	Induction
pedi_ALL	0-4	1	High risk	2	Consolidation
pedi_ALL	0-4	1	High risk	2	Consolidation
pedi_ALL	0-4	1	High risk	2	Consolidation
pedi_ALL	0-4	1	High risk	2	Consolidation
pedi_ALL	0-4	1	High risk	2	Consolidation
pedi_ALL	0-4	1	High risk	2	Consolidation
pedi_ALL	0-4	1	High risk	2	Consolidation
pedi_ALL	0-4	1	High risk	2	Interim Maintenance
pedi_ALL	0-4	1	High risk	2	Interim Maintenance
pedi_ALL	0-4	1	High risk	2	Interim Maintenance
pedi_ALL	0-4	1	High risk	2	Interim Maintenance
pedi_ALL	0-4	1	High risk	2	Interim Maintenance
pedi_ALL	0-4	1	High risk	2	Delayed Intensification
pedi_ALL	0-4	1	High risk	2	Delayed Intensification
pedi_ALL	0-4	1	High risk	2	Delayed Intensification
pedi_ALL	0-4	1	High risk	2	Delayed Intensification
pedi_ALL	0-4	1	High risk	2	Delayed Intensification
pedi_ALL	0-4	1	High risk	2	Delayed Intensification
pedi_ALL	0-4	1	High risk	2	Delayed Intensification
pedi_ALL	0-4	1	High risk	2	Delayed Intensification

For peer review only

pedi_ALL	0-4	1	High risk	2	Delayed Intensification
pedi_ALL	0-4	1	High risk	2	Maintenance
pedi_ALL	0-4	1	High risk	2	Maintenance
pedi_ALL	0-4	1	High risk	2	Maintenance
pedi_ALL	0-4	1	High risk	2	Maintenance
pedi_ALL	0-4	1	High risk	2	Maintenance
pedi_ALL	0-4	1	Relapse	3	Phase 1
pedi_ALL	0-4	1	Relapse	3	Phase 1
pedi_ALL	0-4	1	Relapse	3	Phase 1
pedi_ALL	0-4	1	Relapse	3	Phase 1
pedi_ALL	0-4	1	Relapse	3	Phase 1
pedi_ALL	0-4	1	Relapse	3	Phase 2
pedi_ALL	0-4	1	Relapse	3	Phase 2
pedi_ALL	0-4	1	Relapse	3	Phase 2
pedi_ALL	0-4	1	Relapse	3	Phase 2
pedi_ALL	0-4	1	Relapse	3	Phase 2
pedi_ALL	0-4	1	Relapse	3	Phase 2
pedi_ALL	0-4	1	Relapse	3	Phase 2
pedi_ALL	0-4	1	Relapse	3	Phase 2
pedi_ALL	0-4	1	Relapse	3	Phase 3
pedi_ALL	0-4	1	Relapse	3	Phase 3
pedi_ALL	0-4	1	Relapse	3	Phase 3
pedi_ALL	0-4	1	Relapse	3	Phase 3
pedi_ALL	0-4	1	Relapse	3	Phase 3
pedi_ALL	0-4	1	Relapse	3	Phase 3
pedi_ALL	0-4	1	Relapse	3	Phase 3
pedi_ALL	0-4	1	Relapse	4	Phase 1
pedi_ALL	0-4	1	Relapse	4	Phase 1
pedi_ALL	0-4	1	Relapse	4	Phase 1
pedi_ALL	0-4	1	Relapse	4	Phase 1
pedi_ALL	0-4	1	Relapse	4	Phase 1
pedi_ALL	0-4	1	Relapse	4	Phase 2
pedi_ALL	0-4	1	Relapse	4	Phase 2
pedi_ALL	0-4	1	Relapse	4	Phase 2
pedi_ALL	0-4	1	Relapse	4	Phase 2
pedi_ALL	0-4	1	Relapse	4	Phase 2
pedi_ALL	0-4	1	Relapse	4	Phase 2
pedi_ALL	0-4	1	Relapse	4	Phase 2
pedi_ALL	0-4	1	Relapse	4	Phase 2
pedi_ALL	0-4	1	Relapse	4	Phase 3
pedi_ALL	0-4	1	Relapse	4	Phase 3
pedi_ALL	0-4	1	Relapse	4	Phase 3
pedi_ALL	0-4	1	Relapse	4	Phase 3
pedi_ALL	0-4	1	Relapse	4	Phase 3
pedi_ALL	0-4	1	Relapse	4	Phase 3
pedi_ALL	0-4	1	Relapse	4	Phase 3
pedi_ALL	0-4	1	Relapse	4	Phase 4
pedi_ALL	0-4	1	Relapse	3	Phase 5
pedi_ALL	0-4	1	Relapse	3	Phase 5
pedi_ALL	0-4	1	Relapse	3	Phase 5
pedi_ALL	0-4	1	Relapse	3	Phase 5
pedi_ALL	0-4	1	Relapse	3	Phase 5
pedi_ALL	0-4	1	Relapse	3	Phase 5
pedi_ALL	0-4	1	Relapse	3	Phase 5
pedi_ALL	0-4	1	Relapse	3	Phase 5
pedi_ALL	0-4	1	Relapse	3	Phase 5

For peer review only

pedi_ALL	0-4	1	Relapse	3	Phase 6
pedi_ALL	0-4	1	Relapse	3	Phase 6
pedi_ALL	0-4	1	Relapse	3	Phase 6
pedi_ALL	0-4	1	Relapse	3	Phase 6
pedi_ALL	0-4	1	Relapse	3	Phase 6
pedi_ALL	5-9	2	Standard risk	1	Induction
pedi_ALL	5-9	2	Standard risk	1	Induction
pedi_ALL	5-9	2	Standard risk	1	Induction
pedi_ALL	5-9	2	Standard risk	1	Induction
pedi_ALL	5-9	2	Standard risk	1	Consolidation
pedi_ALL	5-9	2	Standard risk	1	Consolidation
pedi_ALL	5-9	2	Standard risk	1	Consolidation
pedi_ALL	5-9	2	Standard risk	1	Interim Maintenance
pedi_ALL	5-9	2	Standard risk	1	Interim Maintenance
pedi_ALL	5-9	2	Standard risk	1	Interim Maintenance
pedi_ALL	5-9	2	Standard risk	1	Interim Maintenance
pedi_ALL	5-9	2	Standard risk	1	Delayed Intensification
pedi_ALL	5-9	2	Standard risk	1	Delayed Intensification
pedi_ALL	5-9	2	Standard risk	1	Delayed Intensification
pedi_ALL	5-9	2	Standard risk	1	Delayed Intensification
pedi_ALL	5-9	2	Standard risk	1	Delayed Intensification
pedi_ALL	5-9	2	Standard risk	1	Delayed Intensification
pedi_ALL	5-9	2	Standard risk	1	Delayed Intensification
pedi_ALL	5-9	2	Standard risk	1	Delayed Intensification
pedi_ALL	5-9	2	Standard risk	1	Maintenance
pedi_ALL	5-9	2	Standard risk	1	Maintenance
pedi_ALL	5-9	2	Standard risk	1	Maintenance
pedi_ALL	5-9	2	Standard risk	1	Maintenance
pedi_ALL	5-9	2	Standard risk	1	Maintenance
pedi_ALL	5-9	2	High risk	2	Induction
pedi_ALL	5-9	2	High risk	2	Induction
pedi_ALL	5-9	2	High risk	2	Induction
pedi_ALL	5-9	2	High risk	2	Induction
pedi_ALL	5-9	2	High risk	2	Induction
pedi_ALL	5-9	2	High risk	2	Consolidation
pedi_ALL	5-9	2	High risk	2	Consolidation
pedi_ALL	5-9	2	High risk	2	Consolidation
pedi_ALL	5-9	2	High risk	2	Consolidation
pedi_ALL	5-9	2	High risk	2	Consolidation
pedi_ALL	5-9	2	High risk	2	Consolidation
pedi_ALL	5-9	2	High risk	2	Consolidation
pedi_ALL	5-9	2	High risk	2	Interim Maintenance
pedi_ALL	5-9	2	High risk	2	Interim Maintenance
pedi_ALL	5-9	2	High risk	2	Interim Maintenance
pedi_ALL	5-9	2	High risk	2	Interim Maintenance
pedi_ALL	5-9	2	High risk	2	Interim Maintenance
pedi_ALL	5-9	2	High risk	2	Delayed Intensification
pedi_ALL	5-9	2	High risk	2	Delayed Intensification
pedi_ALL	5-9	2	High risk	2	Delayed Intensification
pedi_ALL	5-9	2	High risk	2	Delayed Intensification
pedi_ALL	5-9	2	High risk	2	Delayed Intensification
pedi_ALL	5-9	2	High risk	2	Delayed Intensification
pedi_ALL	5-9	2	High risk	2	Delayed Intensification
pedi_ALL	5-9	2	High risk	2	Delayed Intensification
pedi_ALL	5-9	2	High risk	2	Delayed Intensification
pedi_ALL	5-9	2	High risk	2	Maintenance

For peer review only

pedi_ALL	5-9	2	High risk	2	Maintenance
pedi_ALL	5-9	2	High risk	2	Maintenance
pedi_ALL	5-9	2	High risk	2	Maintenance
pedi_ALL	5-9	2	High risk	2	Maintenance
pedi_ALL	5-9	2	Relapse	3	Phase 1
pedi_ALL	5-9	2	Relapse	3	Phase 1
pedi_ALL	5-9	2	Relapse	3	Phase 1
pedi_ALL	5-9	2	Relapse	3	Phase 1
pedi_ALL	5-9	2	Relapse	3	Phase 1
pedi_ALL	5-9	2	Relapse	3	Phase 2
pedi_ALL	5-9	2	Relapse	3	Phase 2
pedi_ALL	5-9	2	Relapse	3	Phase 2
pedi_ALL	5-9	2	Relapse	3	Phase 2
pedi_ALL	5-9	2	Relapse	3	Phase 2
pedi_ALL	5-9	2	Relapse	3	Phase 2
pedi_ALL	5-9	2	Relapse	3	Phase 2
pedi_ALL	5-9	2	Relapse	3	Phase 2
pedi_ALL	5-9	2	Relapse	3	Phase 3
pedi_ALL	5-9	2	Relapse	3	Phase 3
pedi_ALL	5-9	2	Relapse	3	Phase 3
pedi_ALL	5-9	2	Relapse	3	Phase 3
pedi_ALL	5-9	2	Relapse	3	Phase 3
pedi_ALL	5-9	2	Relapse	3	Phase 3
pedi_ALL	5-9	2	Relapse	3	Phase 3
pedi_ALL	5-9	2	Relapse	4	Phase 1
pedi_ALL	5-9	2	Relapse	4	Phase 1
pedi_ALL	5-9	2	Relapse	4	Phase 1
pedi_ALL	5-9	2	Relapse	4	Phase 1
pedi_ALL	5-9	2	Relapse	4	Phase 1
pedi_ALL	5-9	2	Relapse	4	Phase 2
pedi_ALL	5-9	2	Relapse	4	Phase 2
pedi_ALL	5-9	2	Relapse	4	Phase 2
pedi_ALL	5-9	2	Relapse	4	Phase 2
pedi_ALL	5-9	2	Relapse	4	Phase 2
pedi_ALL	5-9	2	Relapse	4	Phase 2
pedi_ALL	5-9	2	Relapse	4	Phase 2
pedi_ALL	5-9	2	Relapse	4	Phase 2
pedi_ALL	5-9	2	Relapse	4	Phase 3
pedi_ALL	5-9	2	Relapse	4	Phase 3
pedi_ALL	5-9	2	Relapse	4	Phase 3
pedi_ALL	5-9	2	Relapse	4	Phase 3
pedi_ALL	5-9	2	Relapse	4	Phase 3
pedi_ALL	5-9	2	Relapse	4	Phase 3
pedi_ALL	5-9	2	Relapse	4	Phase 3
pedi_ALL	5-9	2	Relapse	4	Phase 4
pedi_ALL	5-9	2	Relapse	3	Phase 5
pedi_ALL	5-9	2	Relapse	3	Phase 5
pedi_ALL	5-9	2	Relapse	3	Phase 5
pedi_ALL	5-9	2	Relapse	3	Phase 5
pedi_ALL	5-9	2	Relapse	3	Phase 5
pedi_ALL	5-9	2	Relapse	3	Phase 5
pedi_ALL	5-9	2	Relapse	3	Phase 5
pedi_ALL	5-9	2	Relapse	3	Phase 5
pedi_ALL	5-9	2	Relapse	3	Phase 5
pedi_ALL	5-9	2	Relapse	3	Phase 6
pedi_ALL	5-9	2	Relapse	3	Phase 6

For peer review only

pedi_ALL	5-9	2	Relapse	3	Phase 6
pedi_ALL	5-9	2	Relapse	3	Phase 6
pedi_ALL	5-9	2	Relapse	3	Phase 6
pedi_ALL	10-14	3	High risk	2	Induction
pedi_ALL	10-14	3	High risk	2	Induction
pedi_ALL	10-14	3	High risk	2	Induction
pedi_ALL	10-14	3	High risk	2	Induction
pedi_ALL	10-14	3	High risk	2	Induction
pedi_ALL	10-14	3	High risk	2	Consolidation
pedi_ALL	10-14	3	High risk	2	Consolidation
pedi_ALL	10-14	3	High risk	2	Consolidation
pedi_ALL	10-14	3	High risk	2	Consolidation
pedi_ALL	10-14	3	High risk	2	Consolidation
pedi_ALL	10-14	3	High risk	2	Consolidation
pedi_ALL	10-14	3	High risk	2	Consolidation
pedi_ALL	10-14	3	High risk	2	Interim Maintenance
pedi_ALL	10-14	3	High risk	2	Interim Maintenance
pedi_ALL	10-14	3	High risk	2	Interim Maintenance
pedi_ALL	10-14	3	High risk	2	Interim Maintenance
pedi_ALL	10-14	3	High risk	2	Interim Maintenance
pedi_ALL	10-14	3	High risk	2	Delayed Intensification
pedi_ALL	10-14	3	High risk	2	Delayed Intensification
pedi_ALL	10-14	3	High risk	2	Delayed Intensification
pedi_ALL	10-14	3	High risk	2	Delayed Intensification
pedi_ALL	10-14	3	High risk	2	Delayed Intensification
pedi_ALL	10-14	3	High risk	2	Delayed Intensification
pedi_ALL	10-14	3	High risk	2	Delayed Intensification
pedi_ALL	10-14	3	High risk	2	Delayed Intensification
pedi_ALL	10-14	3	High risk	2	Delayed Intensification
pedi_ALL	10-14	3	High risk	2	Maintenance
pedi_ALL	10-14	3	High risk	2	Maintenance
pedi_ALL	10-14	3	High risk	2	Maintenance
pedi_ALL	10-14	3	High risk	2	Maintenance
pedi_ALL	10-14	3	High risk	2	Maintenance
pedi_ALL	10-14	3	Relapse	3	Phase 1
pedi_ALL	10-14	3	Relapse	3	Phase 1
pedi_ALL	10-14	3	Relapse	3	Phase 1
pedi_ALL	10-14	3	Relapse	3	Phase 1
pedi_ALL	10-14	3	Relapse	3	Phase 1
pedi_ALL	10-14	3	Relapse	3	Phase 2
pedi_ALL	10-14	3	Relapse	3	Phase 2
pedi_ALL	10-14	3	Relapse	3	Phase 2
pedi_ALL	10-14	3	Relapse	3	Phase 2
pedi_ALL	10-14	3	Relapse	3	Phase 2
pedi_ALL	10-14	3	Relapse	3	Phase 2
pedi_ALL	10-14	3	Relapse	3	Phase 2
pedi_ALL	10-14	3	Relapse	3	Phase 2
pedi_ALL	10-14	3	Relapse	3	Phase 3
pedi_ALL	10-14	3	Relapse	3	Phase 3
pedi_ALL	10-14	3	Relapse	3	Phase 3
pedi_ALL	10-14	3	Relapse	3	Phase 3
pedi_ALL	10-14	3	Relapse	3	Phase 3
pedi_ALL	10-14	3	Relapse	3	Phase 3
pedi_ALL	10-14	3	Relapse	3	Phase 3
pedi_ALL	10-14	3	Relapse	4	Phase 1
pedi_ALL	10-14	3	Relapse	4	Phase 1

For peer review only

pedi_ALL	10-14	3	Relapse	4	Phase 1
pedi_ALL	10-14	3	Relapse	4	Phase 1
pedi_ALL	10-14	3	Relapse	4	Phase 1
pedi_ALL	10-14	3	Relapse	4	Phase 2
pedi_ALL	10-14	3	Relapse	4	Phase 2
pedi_ALL	10-14	3	Relapse	4	Phase 2
pedi_ALL	10-14	3	Relapse	4	Phase 2
pedi_ALL	10-14	3	Relapse	4	Phase 2
pedi_ALL	10-14	3	Relapse	4	Phase 2
pedi_ALL	10-14	3	Relapse	4	Phase 2
pedi_ALL	10-14	3	Relapse	4	Phase 2
pedi_ALL	10-14	3	Relapse	4	Phase 3
pedi_ALL	10-14	3	Relapse	4	Phase 3
pedi_ALL	10-14	3	Relapse	4	Phase 3
pedi_ALL	10-14	3	Relapse	4	Phase 3
pedi_ALL	10-14	3	Relapse	4	Phase 3
pedi_ALL	10-14	3	Relapse	4	Phase 3
pedi_ALL	10-14	3	Relapse	4	Phase 3
pedi_ALL	10-14	3	Relapse	4	Phase 4
pedi_ALL	10-14	3	Relapse	3	Phase 5
pedi_ALL	10-14	3	Relapse	3	Phase 5
pedi_ALL	10-14	3	Relapse	3	Phase 5
pedi_ALL	10-14	3	Relapse	3	Phase 5
pedi_ALL	10-14	3	Relapse	3	Phase 5
pedi_ALL	10-14	3	Relapse	3	Phase 5
pedi_ALL	10-14	3	Relapse	3	Phase 5
pedi_ALL	10-14	3	Relapse	3	Phase 5
pedi_ALL	10-14	3	Relapse	3	Phase 5
pedi_ALL	10-14	3	Relapse	3	Phase 6
pedi_ALL	10-14	3	Relapse	3	Phase 6
pedi_ALL	10-14	3	Relapse	3	Phase 6
pedi_ALL	10-14	3	Relapse	3	Phase 6
pedi_ALL	10-14	3	Relapse	3	Phase 6

For peer review only

Treatment phase (#)	smallest_unit	INN
0	1	Vincristine
0	10,000	Asparaginase
0	5	Prednisolone
0	11	Methotrexate
1	1	Vincristine
1	50	Mercaptopurine
1	11	Methotrexate
2	1	Vincristine
2	50	Methotrexate
2	30	Leucovorin
2	11	Methotrexate
3	1	Vincristine
3	10	Doxorubicin
3	2	Dexamethasone
3	500	Cyclophosphamide
3	40	Tioguanine
3	10,000	Asparaginase
3	11	Methotrexate
3	100	Cytarabine
4	1	Vincristine
4	5	Prednisolone
4	50	Mercaptopurine
4	2.5	Methotrexate
4	11	Methotrexate
0	1	Vincristine
0	10	Doxorubicin
0	10,000	Asparaginase
0	5	Prednisolone
0	11	Methotrexate
1	1	Vincristine
1	50	Mercaptopurine
1	11	Methotrexate
1	100	Cytarabine
1	10,000	Asparaginase
1	400	MESNA
1	500	Cyclophosphamide
2	1	Vincristine
2	50	Methotrexate
2	11	Methotrexate
2	50	Mercaptopurine
2	30	Leucovorin
3	1	Vincristine
3	10	Doxorubicin
3	2	Dexamethasone
3	500	Cyclophosphamide
3	400	MESNA
3	40	Tioguanine
3	10,000	Asparaginase
3	11	Methotrexate

For peer review only

3	100	Cytarabine
4	1	Vincristine
4	5	Prednisolone
4	50	Mercaptopurine
4	11	Methotrexate
4	2.5	Methotrexate
5	1	Vincristine
5	10	Mitoxantrone
5	10,000	Asparaginase
5	11	Methotrexate
5	2	Dexamethasone
6	2	Dexamethasone
6	1	Vincristine
6	50	Methotrexate
6	30	Leucovorin
6	10,000	Asparaginase
6	500	Cyclophosphamide
6	100	Etoposide
6	11	Methotrexate
7	2	Dexamethasone
7	1	Vincristine
7	100	Cytarabine
7	10,000	Asparaginase
7	50	Methotrexate
7	30	Leucovorin
7	11	Methotrexate
5	1	Vincristine
5	10	Mitoxantrone
5	10,000	Asparaginase
5	11	Methotrexate
5	2	Dexamethasone
6	2	Dexamethasone
6	1	Vincristine
6	50	Methotrexate
6	30	Leucovorin
6	10,000	Asparaginase
6	500	Cyclophosphamide
6	100	Etoposide
6	11	Methotrexate
7	2	Dexamethasone
7	1	Vincristine
7	100	Cytarabine
7	10,000	Asparaginase
7	50	Methotrexate
7	30	Leucovorin
7	11	Methotrexate
8	100	Cytarabine
9	2	Dexamethasone
9	1	Vincristine
9	50	Mercaptopurine
9	2.5	Methotrexate
9	2.5	Methotrexate
9	500	Cyclophosphamide
9	100	Etoposide
9	100	Cytarabine
9	11	Methotrexate

For peer review only

10	2	Dexamethasone
10	1	Vincristine
10	50	Mercaptopurine
10	2.5	Methotrexate
10	11	Methotrexate
0	1	Vincristine
0	10,000	Asparaginase
0	5	Prednisolone
0	11	Methotrexate
1	1	Vincristine
1	50	Mercaptopurine
1	11	Methotrexate
2	1	Vincristine
2	50	Methotrexate
2	30	Leucovorin
2	11	Methotrexate
3	1	Vincristine
3	10	Doxorubicin
3	2	Dexamethasone
3	500	Cyclophosphamide
3	40	Tioguanine
3	10,000	Asparaginase
3	11	Methotrexate
3	100	Cytarabine
4	1	Vincristine
4	5	Prednisolone
4	50	Mercaptopurine
4	2.5	Methotrexate
4	11	Methotrexate
0	1	Vincristine
0	10	Doxorubicin
0	10,000	Asparaginase
0	5	Prednisolone
0	11	Methotrexate
1	1	Vincristine
1	50	Mercaptopurine
1	11	Methotrexate
1	100	Cytarabine
1	10,000	Asparaginase
1	400	MESNA
1	500	Cyclophosphamide
2	1	Vincristine
2	50	Methotrexate
2	11	Methotrexate
2	50	Mercaptopurine
2	30	Leucovorin
3	1	Vincristine
3	10	Doxorubicin
3	2	Dexamethasone
3	500	Cyclophosphamide
3	400	MESNA
3	40	Tioguanine
3	10,000	Asparaginase
3	11	Methotrexate
3	100	Cytarabine
4	1	Vincristine

For peer review only

4	5	Prednisolone
4	50	Mercaptopurine
4	11	Methotrexate
4	2.5	Methotrexate
5	1	Vincristine
5	10	Mitoxantrone
5	10,000	Asparaginase
5	11	Methotrexate
5	2	Dexamethasone
6	2	Dexamethasone
6	1	Vincristine
6	50	Methotrexate
6	30	Leucovorin
6	10,000	Asparaginase
6	500	Cyclophosphamide
6	100	Etoposide
6	11	Methotrexate
7	2	Dexamethasone
7	1	Vincristine
7	100	Cytarabine
7	10,000	Asparaginase
7	50	Methotrexate
7	30	Leucovorin
7	11	Methotrexate
5	1	Vincristine
5	10	Mitoxantrone
5	10,000	Asparaginase
5	11	Methotrexate
5	2	Dexamethasone
6	2	Dexamethasone
6	1	Vincristine
6	50	Methotrexate
6	30	Leucovorin
6	10,000	Asparaginase
6	500	Cyclophosphamide
6	100	Etoposide
6	11	Methotrexate
7	2	Dexamethasone
7	1	Vincristine
7	100	Cytarabine
7	10,000	Asparaginase
7	50	Methotrexate
7	30	Leucovorin
7	11	Methotrexate
8	100	Cytarabine
9	2	Dexamethasone
9	1	Vincristine
9	50	Mercaptopurine
9	2.5	Methotrexate
9	2.5	Methotrexate
9	500	Cyclophosphamide
9	100	Etoposide
9	100	Cytarabine
9	11	Methotrexate
10	2	Dexamethasone
10	1	Vincristine

For peer review only

10	50	Mercaptopurine
10	2.5	Methotrexate
10	11	Methotrexate
0	1	Vincristine
0	10	Doxorubicin
0	10,000	Asparaginase
0	5	Prednisolone
0	11	Methotrexate
1	1	Vincristine
1	50	Mercaptopurine
1	11	Methotrexate
1	100	Cytarabine
1	10,000	Asparaginase
1	400	MESNA
1	500	Cyclophosphamide
2	1	Vincristine
2	50	Methotrexate
2	11	Methotrexate
2	50	Mercaptopurine
2	30	Leucovorin
3	1	Vincristine
3	10	Doxorubicin
3	2	Dexamethasone
3	500	Cyclophosphamide
3	400	MESNA
3	40	Tioguanine
3	10,000	Asparaginase
3	11	Methotrexate
3	100	Cytarabine
4	1	Vincristine
4	5	Prednisolone
4	50	Mercaptopurine
4	11	Methotrexate
4	2.5	Methotrexate
5	1	Vincristine
5	10	Mitoxantrone
5	10,000	Asparaginase
5	11	Methotrexate
5	2	Dexamethasone
6	2	Dexamethasone
6	1	Vincristine
6	50	Methotrexate
6	30	Leucovorin
6	10,000	Asparaginase
6	500	Cyclophosphamide
6	100	Etoposide
6	11	Methotrexate
7	2	Dexamethasone
7	1	Vincristine
7	100	Cytarabine
7	10,000	Asparaginase
7	50	Methotrexate
7	30	Leucovorin
7	11	Methotrexate
5	1	Vincristine
5	10	Mitoxantrone

For peer review only

5	10,000	Asparaginase
5	11	Methotrexate
5	2	Dexamethasone
6	2	Dexamethasone
6	1	Vincristine
6	50	Methotrexate
6	30	Leucovorin
6	10,000	Asparaginase
6	500	Cyclophosphamide
6	100	Etoposide
6	11	Methotrexate
7	2	Dexamethasone
7	1	Vincristine
7	100	Cytarabine
7	10,000	Asparaginase
7	50	Methotrexate
7	30	Leucovorin
7	11	Methotrexate
8	100	Cytarabine
9	2	Dexamethasone
9	1	Vincristine
9	50	Mercaptopurine
9	2.5	Methotrexate
9	2.5	Methotrexate
9	500	Cyclophosphamide
9	100	Etoposide
9	100	Cytarabine
9	11	Methotrexate
10	2	Dexamethasone
10	1	Vincristine
10	50	Mercaptopurine
10	2.5	Methotrexate
10	11	Methotrexate

For peer review only

Dosage and formulation according to Thai guidelines	form	oral	IV	IM
1.5 mg/m2/dose IV push (Max 2 mg)	IV		1	
10,000 IU/m2/dose IM	IM			1
30 mg/m2/dose PO BID (No max dose)	tab	1		
	IV			
1.5 mg/m2/dose IV push (Max 2 mg)	IV		1	
75 mg/m2/dose PO daily (hs)	tab	1		
	IV			
1.5 mg/m2/dose IV push (Max 2 mg)	IV		1	
2.5 gm/m2/dose IV infusion in 24 hr (No max	IV		1	
15 mg/m2/dose PO/IV after each HD MTX infu	IV	1	1	
	IV			
1.5 mg/m2/dose IV push (Max 2 mg)	IV		1	
25 mg/m2/dose IV	IV		1	
5 mg/m2/dose PO BID	tab	1		
1000 mg/m2/dose IV	IV			
60 mg/m2/dose PO daily (hs)	tab	1		
10,000 IU/m2/dose IM	IM			1
	IV			
75 mg/m2/dose IV over 15-30 min or SC daily	IV		1	
1.5 mg/m2/dose IV push (Max 2 mg)	IV		1	
20 mg/m2/dose PO BID	tab	1		
50 mg/m2/dose PO daily (hs)	tab	1		
20 mg/m2/dose PO weekly	tab	1		
	IV			
1.5 mg/m2/dose IV push over 1 min (Max 2 m	IV		1	
25 mg/m2/dose IV push	IV		1	
10,000 IU/m2/dose IM	IM			1
30 mg/m2/dose PO BID (No max dose)	tab	1		
	IV			
1.5 mg/m2/dose IV push	IV		1	
60 mg/m2/dose PO daily (hs)	tab	1		
	IV			
75 mg/m2/dose IV or SC daily	IV		1	
25,000 IU/m2/dose IM	IM			1
250 mg/m2/dose IV drip in 15 minutes at hr 0	IV		1	
1,000 mg/m2/dose IV	IV		1	
1.5 mg/m2/dose IV push (Max 2 mg)	IV		1	
5 gm/m2/dose IV infusion (No max dose)	IV		1	
	IV			
25 mg/m2/dose PO daily (hs)	tab	1		
15 mg/m2/dose PO/IV	IV	1	1	
1.5 mg/m2/dose IV push (Max 2 mg)	IV		1	
25 mg/m2/dose IV	IV		1	
5 mg/m2/dose PO BID	tab	1		
1,000 mg/m2/dose IV	IV		1	
250 mg/m2/dose IV drip	IV		1	
60 mg/m2/dose PO daily (hs)	tab	1		
10,000 IU/m2/dose IM	IM			1
	IV			

For peer review only

75 mg/m2/dose IV or SC daily	IV		1	
1.5 mg/m2/dose IV push (Max 2 mg)	IV		1	
20 mg/m2/dose PO BID	tab	1		
50 mg/m2/dose PO daily (hs)	tab	1		
	IV			
20 mg/m2/dose PO	tab	1		
1.5 mg/m2 IV push (max 2 mg)	IV		1	
10 mg/m2 IV	IV		1	
10,000 unit/m2 IM	IM			1
	IV			
20 mg/m2/day PO/IV	tab	1	1	
6 mg/m2/day PO BID	tab	1		
1.5 mg/m2 IV push (max 2 mg)	IV		1	
1,000 mg/m2 IV drip	IV		1	
15 mg/m2 IV	IV		1	
10,000 unit/m2 IM	IM			1
440 mg/m2 IV drip	IV		1	
100 mg/m2 IV drip	IV		1	
	IV			
6 mg/m2/day PO BID	tab	1		
1.5 mg/m2IV push (max 2 mg)	IV		1	
3,000 mg IV drip	IV		1	
10,000 unit/m2 IM	IM			1
1,000 mg/m2 IV drip	IV		1	
15 mg/m2 IV	IV		1	
	IV			
1.5 mg/m2 IV push (max 2 mg)	IV		1	
10 mg/m2 IV	IV		1	
10,000 unit/m2 IM	IM			1
	IV			
20 mg/m2/day PO/IV	tab	1	1	
6 mg/m2/day PO BID	tab	1		
1.5 mg/m2 IV push (max 2 mg)	IV		1	
1,000 mg/m2 IV drip	IV		1	
15 mg/m2 IV	IV		1	
10,000 unit/m2 IM	IM			1
440 mg/m2 IV drip	IV		1	
100 mg/m2 IV drip	IV		1	
	IV			
6 mg/m2/day PO BID	tab	1		
1.5 mg/m2IV push (max 2 mg)	IV		1	
3,000 mg IV drip	IV		1	
10,000 unit/m2 IM	IM			1
1,000 mg/m2 IV drip	IV		1	
15 mg/m2 IV	IV		1	
	IV			
1,000 mg/m2 IV drip	IV		1	
6 mg/m2/day PO BID	tab	1		
1.5 mg /m2 IV push (max 2 mg)	IV		1	
75 mg/m2 PO hs	tab	1		
20 mg/m2 PO hs	tab	1		
25 mg/m2/dose PO	tab	1		
300 mg/m2 IV drip	IV		1	
150 mg/m2 IV drip	IV		1	
50 mg/m2 IV/SC	IV		1	
	IV			

For peer review only

6 mg/m2/day PO BID	tab	1		
1.5 mg/m2 IV push (max 2 mg)	IV		1	
75 mg/m2 PO	tab	1		
20 mg/m2 PO	tab	1		
	IV			
1.5 mg/m2/dose IV push (Max 2 mg)	IV		1	
10,000 IU/m2/dose IM	IM			1
30 mg/m2/dose PO BID (No max dose)	tab	1		
	IV			
1.5 mg/m2/dose IV push (Max 2 mg)	IV		1	
75 mg/m2/dose PO daily (hs)	tab	1		
	IV			
1.5 mg/m2/dose IV push (Max 2 mg)	IV		1	
2.5 gm/m2/dose IV infusion in 24 hr (No max	IV		1	
15 mg/m2/dose PO/IV after each HD MTX infu	IV	1	1	
	IV			
1.5 mg/m2/dose IV push (Max 2 mg)	IV		1	
25 mg/m2/dose IV	IV		1	
5 mg/m2/dose PO BID	tab	1		
1000 mg/m2/dose IV	IV			
60 mg/m2/dose PO daily (hs)	tab	1		
10,000 IU/m2/dose IM	IM			1
	IV			
75 mg/m2/dose IV over 15-30 min or SC daily	IV		1	
1.5 mg/m2/dose IV push (Max 2 mg)	IV		1	
20 mg/m2/dose PO BID	tab	1		
50 mg/m2/dose PO daily (hs)	tab	1		
20 mg/m2/dose PO weekly	tab	1		
	IV			
1.5 mg/m2/dose IV push over 1 min (Max 2 m	IV		1	
25 mg/m2/dose IV push	IV		1	
10,000 IU/m2/dose IM	IM			1
30 mg/m2/dose PO BID (No max dose)	tab	1		
	IV			
1.5 mg/m2/dose IV push	IV		1	
60 mg/m2/dose PO daily (hs)	tab	1		
	IV			
75 mg/m2/dose IV or SC daily	IV		1	
25,000 IU/m2/dose IM	IM			1
250 mg/m2/dose IV drip in 15 minutes at hr 0	IV		1	
1,000 mg/m2/dose IV	IV		1	
1.5 mg/m2/dose IV push (Max 2 mg)	IV		1	
5 gm/m2/dose IV infusion (No max dose)	IV		1	
	IV			
25 mg/m2/dose PO daily (hs)	tab	1		
15 mg/m2/dose PO/IV	IV			
1.5 mg/m2/dose IV push (Max 2 mg)	IV		1	
25 mg/m2/dose IV	IV		1	
5 mg/m2/dose PO BID	tab	1		
1,000 mg/m2/dose IV	IV		1	
250 mg/m2/dose IV drip	IV		1	
60 mg/m2/dose PO daily (hs)	tab	1		
10,000 IU/m2/dose IM	IM			1
	IV			
75 mg/m2/dose IV or SC daily	IV		1	
1.5 mg/m2/dose IV push (Max 2 mg)	IV		1	

For peer review only

For peer review only - http://bmjopen.bmj.com/site/about/guidelines.xhtml

20 mg/m2/dose PO BID	tab	1		
50 mg/m2/dose PO daily (hs)	tab	1		
	IV			
20 mg/m2/dose PO	tab	1		
1.5 mg/m2 IV push (max 2 mg)	IV		1	
10 mg/m2 IV	IV		1	
10,000 unit/m2 IM	IM			1
	IV			
20 mg/m2/day PO/IV	tab	1	1	
6 mg/m2/day PO BID	tab	1		
1.5 mg/m2 IV push (max 2 mg)	IV		1	
1,000 mg/m2 IV drip	IV		1	
15 mg/m2 IV	IV		1	
10,000 unit/m2 IM	IM			1
440 mg/m2 IV drip	IV		1	
100 mg/m2 IV drip	IV		1	
	IV			
6 mg/m2/day PO BID	tab	1		
1.5 mg/m2IV push (max 2 mg)	IV		1	
3,000 mg IV drip	IV		1	
10,000 unit/m2 IM	IM			1
1,000 mg/m2 IV drip	IV		1	
15 mg/m2 IV	IV		1	
	IV			
1.5 mg/m2 IV push (max 2 mg)	IV		1	
10 mg/m2 IV	IV		1	
10,000 unit/m2 IM	IM			1
	IV			
20 mg/m2/day PO/IV	tab	1	1	
6 mg/m2/day PO BID	tab	1		
1.5 mg/m2 IV push (max 2 mg)	IV		1	
1,000 mg/m2 IV drip	IV		1	
15 mg/m2 IV	IV		1	
10,000 unit/m2 IM	IM			1
440 mg/m2 IV drip	IV		1	
100 mg/m2 IV drip	IV		1	
	IV			
6 mg/m2/day PO BID	tab	1		
1.5 mg/m2IV push (max 2 mg)	IV		1	
3,000 mg IV drip	IV		1	
10,000 unit/m2 IM	IM			1
1,000 mg/m2 IV drip	IV		1	
15 mg/m2 IV	IV		1	
	IV			
1,000 mg/m2 IV drip	IV		1	
6 mg/m2/day PO BID	tab	1		
1.5 mg /m2 IV push (max 2 mg)	IV		1	
75 mg/m2 PO hs	tab	1		
20 mg/m2 PO hs	tab	1		
25 mg/m2/dose PO	tab	1		
300 mg/m2 IV drip	IV		1	
150 mg/m2 IV drip	IV		1	
50 mg/m2 IV/SC	IV		1	
	IV			
6 mg/m2/day PO BID	tab	1		
1.5 mg/m2 IV push (max 2 mg)	IV		1	

For peer review only

75 mg/m2 PO	tab	1		
20 mg/m2 PO	tab	1		
	IV			
1.5 mg/m2/dose IV push over 1 min (Max 2 m	IV		1	
25 mg/m2/dose IV push	IV		1	
10,000 IU/m2/dose IM	IM			1
30 mg/m2/dose PO BID (No max dose)	tab	1		
	IV			
1.5 mg/m2/dose IV push	IV		1	
60 mg/m2/dose PO daily (hs)	tab	1		
	IV			
75 mg/m2/dose IV or SC daily	IV		1	
25,000 IU/m2/dose IM	IM			1
250 mg/m2/dose IV drip in 15 minutes at hr 0	IV		1	
1,000 mg/m2/dose IV	IV		1	
1.5 mg/m2/dose IV push (Max 2 mg)	IV		1	
5 gm/m2/dose IV infusion (No max dose)	IV		1	
	IV			
25 mg/m2/dose PO daily (hs)	tab	1		
15 mg/m2/dose PO/IV	IV			
1.5 mg/m2/dose IV push (Max 2 mg)	IV		1	
25 mg/m2/dose IV	IV		1	
5 mg/m2/dose PO BID	tab	1		
1,000 mg/m2/dose IV	IV		1	
250 mg/m2/dose IV drip	IV		1	
60 mg/m2/dose PO daily (hs)	tab	1		
10,000 IU/m2/dose IM	IM			1
	IV			
75 mg/m2/dose IV or SC daily	IV		1	
1.5 mg/m2/dose IV push (Max 2 mg)	IV		1	
20 mg/m2/dose PO BID	tab	1		
50 mg/m2/dose PO daily (hs)	tab	1		
	IV			
20 mg/m2/dose PO	tab	1		
1.5 mg/m2 IV push (max 2 mg)	IV		1	
10 mg/m2 IV	IV		1	
10,000 unit/m2 IM	IM			1
	IV			
20 mg/m2/day PO/IV	tab	1	1	
6 mg/m2/day PO BID	tab	1		
1.5 mg/m2 IV push (max 2 mg)	IV		1	
1,000 mg/m2 IV drip	IV		1	
15 mg/m2 IV	IV		1	
10,000 unit/m2 IM	IM			1
440 mg/m2 IV drip	IV		1	
100 mg/m2 IV drip	IV		1	
	IV			
6 mg/m2/day PO BID	tab	1		
1.5 mg/m2IV push (max 2 mg)	IV		1	
3,000 mg IV drip	IV		1	
10,000 unit/m2 IM	IM			1
1,000 mg/m2 IV drip	IV		1	
15 mg/m2 IV	IV		1	
	IV			
1.5 mg/m2 IV push (max 2 mg)	IV		1	
10 mg/m2 IV	IV		1	

For peer review only

10,000 unit/m2 IM	IM			1
	IV			
20 mg/m2/day PO/IV	tab	1	1	
6 mg/m2/day PO BID	tab	1		
1.5 mg/m2 IV push (max 2 mg)	IV		1	
1,000 mg/m2 IV drip	IV		1	
15 mg/m2 IV	IV		1	
10,000 unit/m2 IM	IM			1
440 mg/m2 IV drip	IV		1	
100 mg/m2 IV drip	IV		1	
	IV			
6 mg/m2/day PO BID	tab	1		
1.5 mg/m2IV push (max 2 mg)	IV		1	
3,000 mg IV drip	IV		1	
10,000 unit/m2 IM	IM			1
1,000 mg/m2 IV drip	IV		1	
15 mg/m2 IV	IV		1	
	IV			
1,000 mg/m2 IV drip	IV		1	
6 mg/m2/day PO BID	tab	1		
1.5 mg /m2 IV push (max 2 mg)	IV		1	
75 mg/m2 PO hs	tab	1		
20 mg/m2 PO hs	tab	1		
25 mg/m2/dose PO	tab	1		
300 mg/m2 IV drip	IV		1	
150 mg/m2 IV drip	IV		1	
50 mg/m2 IV/SC	IV		1	
	IV			
6 mg/m2/day PO BID	tab	1		
1.5 mg/m2 IV push (max 2 mg)	IV		1	
75 mg/m2 PO	tab	1		
20 mg/m2 PO	tab	1		
	IV			

For peer review only

IT	SC	mg_dose	dose_unit	Average Amount (mg dose * BSA)/Person Dose (mg)	Number of Doses/Cycle	Average Amount/Cycle (mg)
		1.5	mg/m2	0.832	4	3.327
		10,000	IU/m2	5544.609	6	33267.655
		30	mg/m2	16.634	28	465.747
1		11	mg	10.500	3	31.500
		2	mg/m2	0.832	2	1.663
		75	mg/m2	41.585	28	1164.368
1		11	mg	10.500	2	21.000
		1.5	mg/m2	0.832	4	3.327
1		2,500	mg/m2	1386.152	4	5544.609
		15	mg/m2	8.317	24	199.606
1		11	mg	10.500	4	42.000
		1.5	mg/m2	0.832	3	2.495
		25	mg/m2	13.862	3	41.585
		5	mg/m2	2.772	14	38.812
		1,000	mg/m2	554.461	1	554.461
		60	mg/m2	33.268	14	465.747
		10,000	IU/m2	5544.609	2	11089.218
1		11	mg	10.500	2	21.000
		75	mg/m2	41.585	8	332.677
		1.5	mg/m2	0.832	3	2.495
		20	mg/m2	11.089	15	166.338
		50	mg/m2	27.723	84	2328.736
		20	mg/m2	11.089	12	133.071
1		11	mg	10.500	1	10.500
		1.5	mg/m2	0.832	4	3.327
		25	mg/m2	13.862	4	55.446
		10,000	IU/m2	5544.609	6	33267.655
		30	mg/m2	16.634	28	465.747
1		11	mg	10.500	3	31.500
		1.5	mg/m2	0.832	4	3.327
		60	mg/m2	33.268	28	931.494
1		11	mg	10.500	4	42.000
	1	75	mg/m2	41.585	16	665.353
		25,000	IU/m2	13861.523	4	55446.091
		250	mg/m2	138.615	6	831.691
		1,000	mg/m2	554.461	2	1108.922
		1.5	mg/m2	0.832	4	3.327
		5,000	gm/m2	2772.305	4	11089.218
1		11	mg	10.500	2	21.000
		25	mg/m2	13.862	56	776.245
		15	mg/m2	8.317	24	199.606
		1.5	mg/m2	0.832	5	4.158
		25	mg/m2	13.862	3	41.585
		5	mg/m2	2.772	14	38.812
		1,000	mg/m2	554.461	1	554.461
		250	mg/m2	138.615	3	415.846
		60	mg/m2	33.268	14	465.747
		10,000	IU/m2	5544.609	4	22178.436
1		11	mg	10.500	3	31.500

For peer review only

	1	75	mg/m2	41.585	8	332.677
		1.5	mg/m2	0.832	3	2.495
		20	mg/m2	11.089	15	166.338
		50	mg/m2	27.723	84	2328.736
1		11	mg	10.500	1	10.500
		20	mg/m2	11.089	12	133.071
		2	mg/m2	0.832	4	3.327
		10	mg/m2	5.545	2	11.089
		10,000	IU/m2	5544.609	6	33267.655
1		11	mg	10.500	2	21.000
		20	mg/m2	11.089	10	110.892
		6	mg/m2	3.327	5	16.634
		2	mg/m2	0.832	1	0.832
		1,000	mg/m2	554.461	1	554.461
		15	mg/m2	8.317	6	49.901
		10,000	IU/m2	5544.609	3	16633.827
		440	mg/m2	243.963	5	1219.814
		100	mg/m2	55.446	5	277.230
1		11	mg	10.500	1	10.500
		6	mg/m2	3.327	5	16.634
		2	mg/m2	0.832	1	0.832
		3,000	mg/m2	1663.383	8	13307.062
		10,000	IU/m2	5544.609	5	27723.046
		1,000	mg/m2	554.461	1	554.461
		15	mg/m2	8.317	6	49.901
1		11	mg	10.500	2	21.000
		2	mg/m2	0.832	4	3.327
		10	mg/m2	5.545	2	11.089
		10,000	IU/m2	5544.609	6	33267.655
1		11	mg	10.500	2	21.000
		20	mg/m2	11.089	10	110.892
		6	mg/m2	3.327	5	16.634
		2	mg/m2	0.832	1	0.832
		1,000	mg/m2	554.461	1	554.461
		15	mg/m2	8.317	6	49.901
		10,000	IU/m2	5544.609	3	16633.827
		440	mg/m2	243.963	5	1219.814
		100	mg/m2	55.446	5	277.230
1		11	mg	10.500	1	10.500
		6	mg/m2	3.327	5	16.634
		2	mg/m2	0.832	1	0.832
		3,000	mg/m2	1663.383	8	13307.062
		10,000	IU/m2	5544.609	5	27723.046
		1,000	mg/m2	554.461	1	554.461
		15	mg/m2	8.317	6	49.901
1		11	mg	10.500	2	21.000
		1,000	mg/m2	554.461	5	2772.305
		6	mg/m2	3.327	5	16.634
		1.5	mg/m2	0.832	1	0.832
		75	mg/m2	41.585	42	1746.552
		20	mg/m2	11.089	4	44.357
		25	mg/m2	13.862	4	55.446
		300	mg/m2	166.338	2	332.677
		150	mg/m2	83.169	2	166.338
	1	50	mg/m2	27.723	8	221.784
1		10.5	mg	10.500	2	21.000

For peer review only

		6	mg/m2	3.327	5	16.634
		1.5	mg/m2	0.832	1	0.832
		75	mg/m2	41.585	525	21831.898
		20	mg/m2	11.089	75	831.691
1		10.5	mg	10.500	6	63.000
		1.5	mg/m2	1.324	4	5.297
		10,000	IU/m2	8829.028	6	52974.166
		30	mg/m2	26.487	28	741.638
1		11	mg	10.500	3	31.500
		2	mg/m2	1.324	2	2.649
		75	mg/m2	66.218	28	1854.096
1		11	mg	10.500	2	21.000
		1.5	mg/m2	1.324	4	5.297
1		2,500	mg/m2	2207.257	4	8829.028
		15	mg/m2	13.244	24	317.845
1		11	mg	10.500	4	42.000
		1.5	mg/m2	1.324	3	3.973
		25	mg/m2	22.073	3	66.218
		5	mg/m2	4.415	14	61.803
		1,000	mg/m2	882.903	1	882.903
		60	mg/m2	52.974	14	741.638
		10,000	IU/m2	8829.028	2	17658.055
1		11	mg	10.500	2	21.000
		75	mg/m2	66.218	8	529.742
		1.5	mg/m2	1.324	3	3.973
		20	mg/m2	17.658	15	264.871
		50	mg/m2	44.145	84	3708.192
		20	mg/m2	17.658	12	211.897
1		11	mg	10.500	1	10.500
		1.5	mg/m2	1.324	4	5.297
		25	mg/m2	22.073	4	88.290
		10,000	IU/m2	8829.028	6	52974.166
		30	mg/m2	26.487	28	741.638
1		11	mg	10.500	3	31.500
		1.5	mg/m2	1.324	4	5.297
		60	mg/m2	52.974	28	1483.277
1		11	mg	10.500	4	42.000
	1	75	mg/m2	66.218	16	1059.483
		25,000	IU/m2	22072.569	4	88290.276
		250	mg/m2	220.726	6	1324.354
		1,000	mg/m2	882.903	2	1765.806
		1.5	mg/m2	1.324	4	5.297
		5,000	gm/m2	4414.514	4	17658.055
1		11	mg	10.500	2	21.000
		25	mg/m2	22.073	56	1236.064
		15	mg/m2	13.244	24	317.845
		1.5	mg/m2	1.324	5	6.622
		25	mg/m2	22.073	3	66.218
		5	mg/m2	4.415	14	61.803
		1,000	mg/m2	882.903	1	882.903
		250	mg/m2	220.726	3	662.177
		60	mg/m2	52.974	14	741.638
		10,000	IU/m2	8829.028	4	35316.111
1		11	mg	10.500	3	31.500
	1	75	mg/m2	66.218	8	529.742
		1.5	mg/m2	1.324	3	3.973

For peer review only

		20	mg/m2	17.658	15	264.871
		50	mg/m2	44.145	84	3708.192
1		11	mg	10.500	1	10.500
		20	mg/m2	17.658	12	211.897
		1.5	mg/m2	1.324	4	5.297
		10	mg/m2	8.829	2	17.658
		10,000	IU/m2	8829.028	6	52974.166
1		11	mg	10.500	2	21.000
		20	mg/m2	17.658	10	176.581
		6	mg/m2	5.297	5	26.487
		1.5	mg/m2	1.324	1	1.324
		1,000	mg/m2	882.903	1	882.903
		15	mg/m2	13.244	6	79.461
		10,000	IU/m2	8829.028	3	26487.083
		440	mg/m2	388.477	5	1942.386
		100	mg/m2	88.290	5	441.451
1		11	mg	10.500	1	10.500
		6	mg/m2	5.297	5	26.487
		2	mg/m2	1.324	1	1.324
		3,000	mg/m2	2648.708	8	21189.666
		10,000	IU/m2	8829.028	5	44145.138
		1,000	mg/m2	882.903	1	882.903
		15	mg/m2	13.244	6	79.461
1		11	mg	10.500	2	21.000
		1.5	mg/m2	1.324	4	5.297
		10	mg/m2	8.829	2	17.658
		10,000	IU/m2	8829.028	6	52974.166
1		11	mg	10.500	2	21.000
		20	mg/m2	17.658	10	176.581
		6	mg/m2	5.297	5	26.487
		1.5	mg/m2	1.324	1	1.324
		1,000	mg/m2	882.903	1	882.903
		15	mg/m2	13.244	6	79.461
		10,000	IU/m2	8829.028	3	26487.083
		440	mg/m2	388.477	5	1942.386
		100	mg/m2	88.290	5	441.451
1		11	mg	10.500	1	10.500
		6	mg/m2	5.297	5	26.487
		2	mg/m2	1.324	1	1.324
		3,000	mg/m2	2648.708	8	21189.666
		10,000	IU/m2	8829.028	5	44145.138
		1,000	mg/m2	882.903	1	882.903
		15	mg/m2	13.244	6	79.461
1		11	mg	10.500	2	21.000
		1,000	mg/m2	882.903	5	4414.514
		6	mg/m2	5.297	5	26.487
		1.5	mg/m2	1.324	1	1.324
		75	mg/m2	66.218	42	2781.144
		20	mg/m2	17.658	4	70.632
		25	mg/m2	22.073	4	88.290
		300	mg/m2	264.871	2	529.742
		150	mg/m2	132.435	2	264.871
	1	50	mg/m2	44.145	8	353.161
1		10.5	mg	10.500	2	21.000
		6	mg/m2	5.297	5	26.487
		1.5	mg/m2	1.324	1	1.324

		75	mg/m2	66.218	525	34764.296
		20	mg/m2	17.658	75	1324.354
1		10.5	mg	10.500	6	63.000
		1.5	mg/m2	1.896	4	7.585
		25	mg/m2	31.605	4	126.418
		10,000	IU/m2	12641.820	6	75850.920
		30	mg/m2	37.925	28	1061.913
1		11	mg	10.500	3	31.500
		1.5	mg/m2	1.896	4	7.585
		60	mg/m2	75.851	28	2123.826
1		11	mg	10.500	4	42.000
	1	75	mg/m2	94.814	16	1517.018
		25,000	IU/m2	31604.550	4	126418.200
		250	mg/m2	316.046	6	1896.273
		1,000	mg/m2	1264.182	2	2528.364
		1.5	mg/m2	1.896	4	7.585
		5,000	gm/m2	6320.910	4	25283.640
1		11	mg	10.500	2	21.000
		25	mg/m2	31.605	56	1769.855
		15	mg/m2	18.963	24	455.106
		1.5	mg/m2	1.896	5	9.481
		25	mg/m2	31.605	3	94.814
		5	mg/m2	6.321	14	88.493
		1,000	mg/m2	1264.182	1	1264.182
		250	mg/m2	316.046	3	948.137
		60	mg/m2	75.851	14	1061.913
		10,000	IU/m2	12641.820	4	50567.280
1		11	mg	10.500	3	31.500
	1	75	mg/m2	94.814	8	758.509
		1.5	mg/m2	1.896	3	5.689
		20	mg/m2	25.284	15	379.255
		50	mg/m2	63.209	84	5309.564
1		11	mg	10.500	1	10.500
		20	mg/m2	25.284	12	303.404
		1.5	mg/m2	1.896	4	7.585
		10	mg/m2	12.642	2	25.284
		10,000	IU/m2	12641.820	6	75850.920
1		11	mg	10.500	2	21.000
		20	mg/m2	25.284	10	252.836
		6	mg/m2	7.585	5	37.925
		1.5	mg/m2	1.896	1	1.896
		1,000	mg/m2	1264.182	1	1264.182
		15	mg/m2	18.963	6	113.776
		10,000	IU/m2	12641.820	3	37925.460
		440	mg/m2	556.240	5	2781.200
		100	mg/m2	126.418	5	632.091
1		11	mg	10.500	1	10.500
		6	mg/m2	7.585	5	37.925
		1.5	mg/m2	1.896	1	1.896
		3,000	mg/m2	3792.546	8	30340.368
		10,000	IU/m2	12641.820	5	63209.100
		1,000	mg/m2	1264.182	1	1264.182
		15	mg/m2	18.963	6	113.776
1		10.50	mg	10.500	2	21.000
		1.5	mg/m2	1.896	4	7.585
		10	mg/m2	12.642	2	25.284

For peer review only

		10,000	IU/m2	12641.820	6	75850.920
1		11	mg	10.500	2	21.000
		20	mg/m2	25.284	10	252.836
		6	mg/m2	7.585	5	37.925
		1.5	mg/m2	1.896	1	1.896
		1,000	mg/m2	1264.182	1	1264.182
		15	mg/m2	18.963	6	113.776
		10,000	IU/m2	12641.820	3	37925.460
		440	mg/m2	556.240	5	2781.200
		100	mg/m2	126.418	5	632.091
1		11	mg	10.500	1	10.500
		6	mg/m2	7.585	5	37.925
		1.5	mg/m2	1.896	1	1.896
		3,000	mg/m2	3792.546	8	30340.368
		10,000	IU/m2	12641.820	5	63209.100
		1000	mg/m2	1264.182	1	1264.182
		15	mg/m2	18.963	6	113.776
1		10.5	mg	10.500	2	21.000
		1000	mg/m2	1264.182	5	6320.910
		6	mg/m2	7.585	5	37.925
		1.5	mg/m2	1.896	1	1.896
		75	mg/m2	94.814	42	3982.173
		20	mg/m2	25.284	4	101.135
		25	mg/m2	31.605	4	126.418
		300	mg/m2	379.255	2	758.509
		150	mg/m2	189.627	2	379.255
	1	50	mg/m2	63.209	8	505.673
1		10.5	mg	10.500	2	21.000
		6	mg/m2	7.585	5	37.925
		1.5	mg/m2	1.896	1	1.896
		75	mg/m2	94.814	525	49777.166
		20	mg/m2	25.284	75	1896.273
1		10.5	mg	10.500	6	63.000

For peer review only

Number of Cycles/Case	Average Amount/ Case (mg)	BSA
1	3.326765	0.55
1	33267.65	0.55
1	465.7472	0.55
1	31.5	1.00
1	1.663383	0.55
1	1164.368	0.55
1	21	1.00
1	3.326765	0.55
1	5544.609	0.55
1	199.6059	0.55
1	42	1.00
1	2.495074	0.55
1	41.58457	0.55
1	38.81226	0.55
1	554.4609	0.55
1	465.7472	0.55
1	11089.22	0.55
1	21	1.00
1	332.6765	0.55
9	22.45567	0.55
9	1497.044	0.55
9	20958.62	0.55
9	1197.636	0.55
9	94.5	1.00
1	3.326765	0.55
1	55.44609	0.55
1	33267.65	0.55
1	465.7472	0.55
1	31.5	1.00
1	3.326765	0.55
1	931.4943	0.55
1	42	1.00
1	665.3531	0.55
1	55446.09	0.55
1	831.6914	0.55
1	1108.922	0.55
1	3.326765	0.55
1	11089.22	0.55
1	21	1.00
1	776.2453	0.55
1	199.6059	0.55
1	4.158457	0.55
1	41.58457	0.55
1	38.81226	0.55
1	554.4609	0.55
1	415.8457	0.55
1	465.7472	0.55
1	22178.44	0.55
1	31.5	1.00

	INN	Average Amount/Case (mg) across treatment phases
0-4 years old Standard risk	Asparaginase	44,357
	Cyclophosphamide	554
	Cytarabine	333
	Dexamethasone	39
	Doxorubicin	42
	Leucovorin	200
	Mercaptopurine	22,123
	Methotrexate IV	5,755
	Methotrexate tab	1,198
	Prednisolone	1,963
	Tioguanine	466
	Vincristine	33
0-4 years old High risk	Asparaginase	110,892
	Cyclophosphamide	1,663
	Cytarabine	998
	Dexamethasone	39
	Doxorubicin	97
	Leucovorin	200
	Mercaptopurine	22,666
	MESNA	1,248
	Methotrexate IV	11,310
	Methotrexate tab	1,198
	Prednisolone	1,963
	Tioguanine	466
	Vincristine	37
0-4 years old Relapse SR	Asparaginase	77,625
	Cyclophosphamide	1,885
	Cytarabine	13,751
	Dexamethasone	477
	Etoposide	610
	Leucovorin	100
	Mercaptopurine	25,325
	Methotrexate IV	1,266
	Methotrexate tab	1,031
	Mitoxantrone	11
	Vincristine	22
0-4 years old Relapse HR	Asparaginase	77,625
	Cyclophosphamide	1,220
	Cytarabine	16,079
	Dexamethasone	144
	Etoposide	277
	Leucovorin	100
	Methotrexate IV	1,161
	Mitoxantrone	11

For peer review only

1	332.6765	0.55
9	22.45567	0.55
9	1497.044	0.55
9	20958.62	0.55
9	94.5	1.00
9	1197.636	0.55
1	3.326765	0.55
1	11.08922	0.55
1	33267.65	0.55
1	21	1.00
1	110.8922	0.55
1	16.63383	0.55
1	0.831691	0.55
1	554.4609	0.55
1	49.90148	0.55
1	16633.83	0.55
1	1219.814	0.55
1	277.2305	0.55
1	10.5	1.00
1	16.63383	0.55
1	0.831691	0.55
1	13307.06	0.55
1	27723.05	0.55
1	554.4609	0.55
1	49.90148	0.55
1	21	1.00
1	3.326765	0.55
1	11.08922	0.55
1	33267.65	0.55
1	21	1.00
1	110.8922	0.55
1	16.63383	0.55
1	0.831691	0.55
1	554.4609	0.55
1	49.90148	0.55
1	16633.83	0.55
1	1219.814	0.55
1	277.2305	0.55
1	10.5	1.00
1	16.63383	0.55
1	0.831691	0.55
1	13307.06	0.55
1	27723.05	0.55
1	554.4609	0.55
1	49.90148	0.55
1	21	1.00
1	2772.305	0.55
2	33.26765	0.55
2	1.663383	0.55
2	3493.104	0.55
2	88.71375	0.55
2	110.8922	0.55
2	665.3531	0.55
2	332.6765	0.55
2	443.5687	0.55
2	42	1.00

	Vincristine	5
5-9 years old Standard risk	Asparaginase	70,632.221
	Cyclophosphamide	883
	Cytarabine	530
	Dexamethasone	62
	Doxorubicin	66
	Leucovorin	318
	Mercaptopurine	35,228
	Methotrexate IV	9,039
	Methotrexate tab	1,907
	Prednisolone	3,125
	Tioguanine	742
	Vincristine	53
5-9 years old High risk	Asparaginase	176,581
	Cyclophosphamide	2,649
	Cytarabine	1,589
	Dexamethasone	62
	Doxorubicin	155
	Leucovorin	318
	Mercaptopurine	36,093
	MESNA	1,987
	Methotrexate IV	17,879
	Methotrexate tab	1,907
	Prednisolone	3,125
	Tioguanine	742
	Vincristine	58
5-9 years old Relapse SR	Asparaginase	123,606
	Cyclophosphamide	3,002
	Cytarabine	21,896
	Dexamethasone	759
	Etoposide	971
	Leucovorin	159
	Mercaptopurine	40,327
	Methotrexate IV	1,923
	Methotrexate tab	1,642
	Mitoxantrone	18
	Vincristine	34
5-9 years old Relapse HR	Asparaginase	123,606
	Cyclophosphamide	1,942
	Cytarabine	25,604
	Dexamethasone	230
	Etoposide	441
	Leucovorin	159
	Methotrexate IV	1,818
	Mitoxantrone	18
	Vincristine	8
10-14 years old High risk	Asparaginase	252,836
	Cyclophosphamide	3,793
	Cytarabine	2,276
	Dexamethasone	88

For peer review only

18	299.4089	0.55
18	14.97044	0.55
1	21831.9	0.55
1	831.6914	0.55
1	63	1.00
1	5.297417	0.88
1	52974.17	0.88
1	741.6383	0.88
1	31.5	1.00
1	2.648708	0.88
1	1854.096	0.88
1	21	1.00
1	5.297417	0.88
1	8829.028	0.88
1	317.845	0.88
1	42	1.00
1	3.973062	0.88
1	66.21771	0.88
1	61.80319	0.88
1	882.9028	0.88
1	741.6383	0.88
1	17658.06	0.88
1	21	1.00
1	529.7417	0.88
9	35.75756	0.88
9	2383.837	0.88
9	33373.72	0.88
9	1907.07	0.88
9	94.5	1.00
1	5.297417	0.88
1	88.29028	0.88
1	52974.17	0.88
1	741.6383	0.88
1	31.5	1.00
1	5.297417	0.88
1	1483.277	0.88
1	42	1.00
1	1059.483	0.88
1	88290.28	0.88
1	1324.354	0.88
1	1765.806	0.88
1	5.297417	0.88
1	17658.06	0.88
1	21	1.00
1	1236.064	0.88
1	317.845	0.88
1	6.621771	0.88
1	66.21771	0.88
1	61.80319	0.88
1	882.9028	0.88
1	662.1771	0.88
1	741.6383	0.88
1	35316.11	0.88
1	31.5	1.00
1	529.7417	0.88
9	35.75756	0.88

	Doxorubicin	221
	Leucovorin	455
	Mercaptopurine	51,680
	MESNA	2,844
	Methotrexate IV	25,504
	Methotrexate tab	2,731
	Prednisolone	4,475
	Tioguanine	1,062
	Vincristine	83
10-14 years old Relapse SR	Asparaginase	176,985
	Cyclophosphamide	4,298
	Cytarabine	31,352
	Dexamethasone	1,087
	Etoposide	1,391
	Leucovorin	228
	Mercaptopurine	57,742
	Methotrexate IV	2,686
	Methotrexate tab	2,351
	Mitoxantrone	25
	Vincristine	49
10-14 years old Relapse HR	Asparaginase	176,985
	Cyclophosphamide	2,781
	Cytarabine	36,661
	Dexamethasone	329
	Etoposide	632
	Leucovorin	228
	Methotrexate IV	2,581
	Mitoxantrone	25
	Vincristine	11

For peer review only

9	2383.837	0.88
9	33373.72	0.88
9	94.5	1.00
9	1907.07	0.88
1	5.297417	0.88
1	17.65806	0.88
1	52974.17	0.88
1	21	1.00
1	176.5806	0.88
1	26.48708	0.88
1	1.324354	0.88
1	882.9028	0.88
1	79.46125	0.88
1	26487.08	0.88
1	1942.386	0.88
1	441.4514	0.88
1	10.5	1.00
1	26.48708	0.88
1	1.324354	0.88
1	21189.67	0.88
1	44145.14	0.88
1	882.9028	0.88
1	79.46125	0.88
1	21	1.00
1	5.297417	0.88
1	17.65806	0.88
1	52974.17	0.88
1	21	1.00
1	176.5806	0.88
1	26.48708	0.88
1	1.324354	0.88
1	882.9028	0.88
1	79.46125	0.88
1	26487.08	0.88
1	1942.386	0.88
1	441.4514	0.88
1	10.5	1.00
1	26.48708	0.88
1	1.324354	0.88
1	21189.67	0.88
1	44145.14	0.88
1	882.9028	0.88
1	79.46125	0.88
1	21	1.00
1	4414.514	0.88
2	52.97417	0.88
2	2.648708	0.88
2	5562.287	0.88
2	141.2644	0.88
2	176.5806	0.88
2	1059.483	0.88
2	529.7417	0.88
2	706.3222	0.88
2	42	1.00
18	476.7675	0.88
18	23.83837	0.88

For peer review only

1	34764.3	0.88
1	1324.354	0.88
1	63	1.00
1	7.585092	1.26
1	126.4182	1.26
1	75850.92	1.26
1	1061.913	1.26
1	31.5	1.00
1	7.585092	1.26
1	2123.826	1.26
1	42	1.00
1	1517.018	1.26
1	126418.2	1.26
1	1896.273	1.26
1	2528.364	1.26
1	7.585092	1.26
1	25283.64	1.26
1	21	1.00
1	1769.855	1.26
1	455.1055	1.26
1	9.481365	1.26
1	94.81365	1.26
1	88.49274	1.26
1	1264.182	1.26
1	948.1365	1.26
1	1061.913	1.26
1	50567.28	1.26
1	31.5	1.00
1	758.5092	1.26
9	51.19937	1.26
9	3413.291	1.26
9	47786.08	1.26
9	94.5	1.00
9	2730.633	1.26
1	7.585092	1.26
1	25.28364	1.26
1	75850.92	1.26
1	21	1.00
1	252.8364	1.26
1	37.92546	1.26
1	1.896273	1.26
1	1264.182	1.26
1	113.7764	1.26
1	37925.46	1.26
1	2781.2	1.26
1	632.091	1.26
1	10.5	1.00
1	37.92546	1.26
1	1.896273	1.26
1	30340.37	1.26
1	63209.1	1.26
1	1264.182	1.26
1	113.7764	1.26
1	21	1.00
1	7.585092	1.26
1	25.28364	1.26

For peer review only

1	75850.92	1.26
1	21	1.00
1	252.8364	1.26
1	37.92546	1.26
1	1.896273	1.26
1	1264.182	1.26
1	113.7764	1.26
1	37925.46	1.26
1	2781.2	1.26
1	632.091	1.26
1	10.5	1.00
1	37.92546	1.26
1	1.896273	1.26
1	30340.37	1.26
1	63209.1	1.26
1	1264.182	1.26
1	113.7764	1.26
1	21	1.00
1	6320.91	1.26
2	75.85092	1.26
2	3.792546	1.26
2	7964.347	1.26
2	202.2691	1.26
2	252.8364	1.26
2	1517.018	1.26
2	758.5092	1.26
2	1011.346	1.26
2	42	1.00
18	682.6583	1.26
18	34.13291	1.26
1	49777.17	1.26
1	1896.273	1.26
1	63	1.00

For peer review only

DX 2017	DX + uDX 2017	DX 2021	DX + uDX 2021
4,198,059	7,052,242	3,823,511	6,382,478
52,476	88,153	47,794	79,781
31,485	52,892	28,676	47,869
3,673	6,171	3,346	5,585
3,936	6,611	3,585	5,984
18,891	31,735	17,206	28,721
2,093,782	3,517,306	1,906,976	3,183,261
544,632	914,918	496,041	828,026
113,348	190,411	103,235	172,327
185,764	312,062	169,190	282,425
44,080	74,049	40,147	67,016
3,149	5,289	2,868	4,787
5,677,702	9,537,868	5,171,142	8,632,040
85,166	143,068	77,567	129,481
51,099	85,841	46,540	77,688
1,987	3,338	1,810	3,021
4,968	8,346	4,525	7,553
10,220	17,168	9,308	15,538
1,160,522	1,949,540	1,056,981	1,764,389
63,874	107,301	58,175	97,110
579,060	972,752	527,397	880,368
61,319	103,009	55,848	93,226
100,495	168,820	91,529	152,787
23,846	40,059	21,719	36,255
1,874	3,147	1,706	2,849
687,771	1,155,374	626,408	1,045,646
16,703	28,059	15,213	25,394
121,834	204,666	110,964	185,229
4,225	7,097	3,848	6,423
5,404	9,078	4,922	8,216
884	1,485	805	1,344
224,385	376,941	204,366	341,142
11,221	18,850	10,220	17,059
9,138	15,350	8,322	13,892
98	165	89	149
192	322	174	291
1,767,540	2,969,259	1,609,842	2,687,263
27,776	46,660	25,298	42,228
366,133	615,061	333,467	556,647
3,283	5,514	2,990	4,991
6,313	10,604	5,749	9,597
2,273	3,818	2,070	3,455
26,446	44,426	24,087	40,207
253	424	230	384

Asparaginase
Cyclophosphamide
Cytarabine
Doxorubicin
Etoposide
Mercaptopurine
Methotrexate IV
Methotrexate tab
Mitoxantrone
Tioguanine
Vincristine
Dexamethasone
Leucovorin
MESNA
Prednisolone
Antineoplastic agent
Chemoprotective ag
**Total expenditure on a**
Total health expendi
Percentage of total h

For peer review only

114	191	103	173

4,788,237	8,005,361	4,538,249	7,644,633
59,853	100,067	56,728	95,558
35,912	60,040	34,037	57,335
4,190	7,005	3,971	6,689
4,489	7,505	4,255	7,167
21,547	36,024	20,422	34,401
2,388,133	3,992,674	2,263,452	3,812,761
612,766	1,024,471	580,774	978,308
129,282	216,145	122,533	206,405
211,879	354,237	200,818	338,275
50,276	84,056	47,652	80,269
3,591	6,004	3,404	5,733

6,475,895	10,826,922	6,137,795	10,339,052
97,138	162,404	92,067	155,086
58,283	97,442	55,240	93,051
2,267	3,789	2,148	3,619
5,666	9,474	5,371	9,047
11,657	19,488	11,048	18,610
1,323,673	2,213,023	1,254,565	2,113,302
72,854	121,803	69,050	116,314
655,676	1,096,212	621,444	1,046,816
69,940	116,931	66,288	111,662
114,623	191,637	108,639	183,001
27,199	45,473	25,779	43,424
2,137	3,573	2,025	3,412

784,460	1,311,524	743,504	1,252,425
19,051	31,851	18,057	30,416
138,962	232,327	131,706	221,858
4,819	8,057	4,567	7,693
6,164	10,305	5,842	9,840
1,009	1,686	956	1,610
255,930	427,885	242,568	408,604
12,206	20,407	11,569	19,488
10,422	17,425	9,878	16,639
112	187	106	179
219	365	207	349

2,016,028	3,370,557	1,910,773	3,218,677
31,680	52,966	30,026	50,579
417,606	698,187	395,803	666,726
3,744	6,260	3,549	5,978
7,200	12,038	6,824	11,495
2,592	4,334	2,457	4,138
29,657	49,582	28,108	47,348
288	482	273	460
130	217	123	207

17,082,386	28,616,529	16,425,011	27,285,599
256,236	429,248	246,375	409,284
153,741	257,549	147,825	245,570
5,979	10,016	5,749	9,550

For peer review only

14,947	25,039	14,372	23,875
30,748	51,510	29,565	49,114
3,491,640	5,849,219	3,357,272	5,577,176
192,177	321,936	184,781	306,963
1,723,136	2,886,610	1,656,825	2,752,356
184,490	309,059	177,390	294,684
302,358	506,513	290,723	482,955
71,746	120,189	68,985	114,600
5,637	9,443	5,420	9,004
726,450	1,216,954	698,494	1,160,355
17,642	29,555	16,963	28,180
128,685	215,575	123,733	205,549
4,462	7,476	4,291	7,128
5,708	9,562	5,488	9,117
934	1,565	898	1,492
237,004	397,031	227,884	378,566
11,024	18,468	10,600	17,609
9,651	16,168	9,280	15,416
104	174	100	166
202	339	195	323
1,866,944	3,127,517	1,795,099	2,982,059
29,338	49,147	28,209	46,861
386,724	647,843	371,842	617,712
3,467	5,808	3,334	5,538
6,668	11,170	6,411	10,650
2,400	4,021	2,308	3,834
27,224	45,607	26,177	43,485
267	447	256	426
120	201	115	192

For peer review only

For peer review only

For peer review only

For peer review only

Smallest unit	Total mg 2017		Total smallest units 2017		Exper
	DX	DX+uDX	DX	DX+uDX	DX
10,000	46,071,471	77,190,107	4,607	7,719	225,847
500	693,059	1,161,177	1,386	2,322	6,551
100	1,890,465	3,167,422	18,905	31,674	73,572
10	34,006	56,975	3,401	5,698	9,705
100	37,456	62,756	375	628	2,112
50	11,175,069	18,723,618	223,501	374,472	164,313
50	4,233,049	7,092,303	84,661	141,846	193,746
2.5	587,590	984,496	235,036	393,798	19,285
10	1,121	1,879	112	188	11,586
40	217,147	363,826	5,429	9,096	12,011
1	17,363	29,092	17,363	29,092	70,469
2	42,096	70,530	21,048	35,265	295
30	103,155	172,834	3,438	5,761	21,030
400	328,905	551,040	822	1,378	2,565
5	915,120	1,533,268	183,024	306,654	1,863

:s	789,198
ents	25,754
**antineoplasti and chemoprotective agents**	**814,952**
iture (in million constant (2017) US$)	17,055
ıealth budget	0.005

For peer review only

For peer review only

For peer review only

For peer review only

For peer review only

For peer review only

nditure (US$) 2017		Total mg 2021		Total smallest units 2021	
DX+uDX	Proportion	DX	DX+uDX	DX	DX+uDX
378,394	0.28	43,479,829	72,630,227	4,348	7,263
10,976	0.01	654,297	1,092,848	1,309	2,186
123,268	0.09	1,779,834	2,975,235	17,798	29,752
16,260	0.01	32,106	53,625	3,211	5,363
3,538	0.00	35,236	58,916		589
275,303	0.20	10,514,065	17,579,200	210,281	351,584
324,614	0.24	3,993,241	6,671,071	79,865	133,421
32,311	0.02	552,774	924,252	221,110	369,701
19,413	0.01	1,055	1,764	105	176
20,125	0.01	204,281	341,563	5,107	8,539
118,070	0.09	16,341	27,320	16,341	27,320
494	0.00	39,601	66,214	19,801	33,107
35,236	0.03	97,043	162,258	3,235	5,409
4,298	0.00	312,007	520,388	780	1,301
3,122	0.00	860,899	1,439,443	172,180	287,889
1,322,272					
43,149					
**1,365,422**					
0.008					

For peer review only

For peer review only

For peer review only

For peer review only

For peer review only

For peer review only

Expenditure (US$) 2021	
DX	DX+uDX
236,209	394,571
6,854	11,448
76,763	128,319
10,155	16,961
2,202	3,681
171,324	286,448
202,549	338,377
20,105	33,617
12,081	20,199
12,522	20,938
73,507	122,891
307	514
21,928	36,663
2,401	4,004
1,943	3,249
824,270	1,377,449
26,579	44,430
**850,849**	**1,421,880**

For peer review only

Children

Applied value				
	0-4	5-9	10-14	15-19
**Age group**	1	2	3	4
**Applied BSA**	0.554	0.883	1.264	1.532
**Applied weight**	12.425	23.540	39.305	52.135

Adults

Applied value		
	**Male**	**Female**
BSA	1.80	1.58
weight	68.83	57.40

Age	Boy		Girl		Both Sex
	Weight (kg	leight (cm	Veight (kg	leight (cm	Veight (kg
1	9.65	75.6	9.1	73.85	9.375
2	12.45	87	11.7	85.35	12.075
3	14.65	95.1	14	93.65	14.325
4	16.75	101.85	11.1	100.95	13.925
5	18.8	108.55	18.05	107.5	18.425
6	21	114.5	20.4	114.1	20.7
7	23.55	120.1	23.2	119.6	23.375
8	26.15	125.3	25.9	124.7	26.025
9	29.05	130.05	29.3	130.5	29.175
10	32.2	134.8	32.75	136.6	32.475
11	35.4	139.95	36.3	142.75	35.85
12	39.05	146	39.8	147.85	39.425
13	41.6	152.65	43.05	151.85	42.325
14	47.15	158.65	45.75	154.65	46.45

General Business

For peer review only - http://bmjopen.bmj.com/site/about/guidelines.xhtml

15	51	163.35	47.55	155.95	49.275
16	54	167.1	48.65	156.55	51.325
17	56.05	168.8	49.2	156.85	52.625
18	57.5	169.45	49.5	157	53.5
19	58.15	169.65	49.75	157	53.95

Sources: Weight and heigh of Thai children [Report on the Thai population Health: 2000.
Mosteller R. Simplified Calculation of Body-Surface Area. N Engl J Med. 1987 Oct 22;317(

Average Th	Weight (kg	Height (cm)
Male	68.83	169.46
Female	57.4	157

For peer review only

ตารางแสดงน้ำหนักและส่วนสูงทีควรจะเป็นในช่วงอายุ 0-19 ปีตามเกณฑ์มาตรฐานการเจริญเติบโตของเพศชายและเพศหญิง (อ้างอิงจากกรมอนามัย กระทรวงสาธารณสุข 2543)

For peer review only

	Body Surface Area				
	The Mosteller formula			The Dubois formula	
AGE	Boy	Girl	Both	Boy	Girl
1	0.450	0.432	0.441	0.433	0.415
2	0.549	0.527	0.538	0.535	0.513
3	0.622	0.603	0.613	0.611	0.593
4	0.688	0.558	0.626	0.680	0.567
5	0.753	0.734	0.744	0.748	0.730
6	0.817	0.804	0.811	0.815	0.803
7	0.886	0.878	0.882	0.885	0.877
8	0.954	0.947	0.951	0.955	0.947
9	1.024	1.031	1.028	1.025	1.032
10	1.098	1.115	1.106	1.100	1.118
11	1.173	1.200	1.186	1.176	1.206
12	1.258	1.279	1.268	1.265	1.287
13	1.328	1.348	1.338	1.342	1.356
14	1.441	1.402	1.422	1.455	1.410
15	1.521	1.435	1.478	1.537	1.442
16	1.583	1.455	1.519	1.601	1.460
17	1.621	1.464	1.543	1.638	1.470
18	1.645	1.469	1.557	1.661	1.474
19	1.655	1.473	1.564	1.670	1.478

Average Thai population	
Male	1.800
Female	1.582

1) The Mosteller formula

Aged Gr	1
	0-4 yrs
BSA	0.554
weight	12.425
high	89.169

3) The Haycock formula

Aged Gr	1
	0-4 yrs
BSA	0.557
weight	12.425
high	89.169

5) The Haycock formula

Aged Gr	1
	0-4 yrs
BSA	0.573
weight	12.425
high	89.169

leight (cm)
74.725
86.175
94.375
101.4
108.025
114.3
119.85
125
130.275
135.7
141.35
146.925
152.25
156.65

Age	Aged Gr	Formula 1
0-4 yr	1	0.554
5-9 yr	2	0.883
10-14 yr	3	1.264
15-19 yr	4	1.532

For peer review only - http://bmjopen.bmj.com/site/about/guidelines.xhtml

159.65
161.825
162.825
163.225
163.325

Ministry of Public Health] (in Thai) [Internet]. Available from: https://www.hiso.or.th/his[illegible]
(17):1098–1098.

For peer review only

For peer review only

General Business

The Haycock formula			The Gehan formula			The Boyd form	
Both	Boy	Girl	Both	Boy	Girl	Both	Boy
0.424	0.456	0.438	0.447	0.469	0.451	0.460	0.472
0.524	0.553	0.531	0.542	0.568	0.545	0.556	0.570
0.602	0.625	0.607	0.616	0.641	0.622	0.631	0.643
0.626	0.691	0.552	0.624	0.707	0.570	0.641	0.708
0.739	0.754	0.734	0.744	0.770	0.751	0.761	0.771
0.809	0.817	0.803	0.810	0.834	0.820	0.827	0.834
0.881	0.885	0.877	0.881	0.903	0.894	0.898	0.903
0.951	0.953	0.946	0.949	0.970	0.963	0.967	0.970
1.029	1.023	1.029	1.026	1.040	1.046	1.043	1.041
1.109	1.097	1.113	1.105	1.113	1.129	1.121	1.114
1.191	1.171	1.197	1.184	1.188	1.213	1.200	1.188
1.276	1.256	1.275	1.265	1.272	1.291	1.281	1.271
1.349	1.322	1.344	1.333	1.339	1.360	1.349	1.334
1.433	1.436	1.399	1.418	1.451	1.414	1.433	1.446
1.489	1.516	1.433	1.474	1.530	1.447	1.489	1.524
1.530	1.577	1.453	1.515	1.591	1.467	1.529	1.583
1.554	1.615	1.463	1.539	1.629	1.476	1.553	1.621
1.567	1.640	1.468	1.555	1.653	1.482	1.568	1.646
1.574	1.651	1.472	1.562	1.663	1.485	1.575	1.656

1.793			1.807			1.813	
1.570			1.590			1.599	

2) The Dubois formula							
2	3	4	Aged Gr	1	2	3	4
5-9 yrs	10-14 yr	15-19 yr		0-4 yrs	5-9 yrs	10-14 yr	15-19 yr
0.883	1.264	1.532	BSA	0.544	0.882	1.271	1.543
23.540	39.305	52.135	weight	12.425	23.540	39.305	52.135
119.490	146.575	162.170	high	89.169	119.490	146.575	162.170

4) The Boyd formula							
2	3	4	Aged Gr	1	2	3	4
5-9 yrs	10-14 yr	15-19 yr		0-4 yrs	5-9 yrs	10-14 yr	15-19 yr
0.882	1.261	1.529	BSA	0.572	0.899	1.277	1.542
23.540	39.305	52.135	weight	12.425	23.540	39.305	52.135
119.490	146.575	162.170	high	89.169	119.490	146.575	162.170

2	3	4
5-9 yrs	10-14 yr	15-19 yr
0.899	1.275	1.539
23.540	39.305	52.135
119.490	146.575	162.170

BSA				Weight	Height
Formula 2	Formula 3	Formula 4	Formula 5		
0.544	0.557	0.572	0.573	12.425	89.169
0.882	0.882	0.899	0.899	23.540	119.490
1.271	1.261	1.277	1.275	39.305	146.575
1.543	1.529	1.542	1.539	52.135	162.170

General Business

o/picture/reportHealth/report/report6.pdf (last accessed 9 May 2020)

For peer review only

General Business

For peer review only

General Business

nula	
Girl	Both
0.453	0.463
0.547	0.559
0.623	0.633
0.558	0.637
0.751	0.761
0.820	0.827
0.894	0.898
0.963	0.967
1.047	1.044
1.129	1.122
1.212	1.200
1.289	1.280
1.357	1.346
1.411	1.429
1.446	1.485
1.466	1.525
1.476	1.549
1.481	1.564
1.485	1.571

For peer review only

INN	Formulation	Form	Brand name
Asparaginase	ASPARAGINASE VIAL DRY 0.01 M		LEUNASE
Cyclophosphamide	CYCLOPHOSPHAMIDE VIAL DRY 1 G	IV	ENDOXAN
Cyclophosphamide	CYCLOPHOSPHAMIDE VIAL DRY 1 G	IV	CYCLOXAN
Cyclophosphamide	CYCLOPHOSPHAMIDE VIAL DRY 200 MG	IV	ENDOXAN
Cyclophosphamide	CYCLOPHOSPHAMIDE VIAL DRY 200 MG	IV	CYCLOXAN
Cyclophosphamide	CYCLOPHOSPHAMIDE VIAL DRY 500 MG	IV	ENDOXAN
Cyclophosphamide	CYCLOPHOSPHAMIDE VIAL DRY 500 MG	IV	ONCOMIDE
Cytarabine	CYTARABINE VIAL 1 G /10ML (10 ML)	IV	CYTARINE
Cytarabine	CYTARABINE VIAL 100 MG (1 ML)	IV	CYTARINE
Cytarabine	CYTARABINE VIAL 100 MG /ML (20 ML)	IV	CYTOSAR CS
Cytarabine	CYTARABINE VIAL 20 MG /ML (5 ML)	IV	CYTOSAR CS
Cytarabine	CYTARABINE VIAL 500 MG (5 ML)	IV	CYTARINE
Doxorubicin	DOXORUBICIN VIAL 10 MG /5ML (5 ML)	IV	ADRIM
Doxorubicin	DOXORUBICIN VIAL 2 MG /ML (25 ML)	IV	DOXORUBIN
Doxorubicin	DOXORUBICIN VIAL 2 MG /ML (5 ML)	IV	DOXORUBIN
Doxorubicin	DOXORUBICIN VIAL 50 MG (25 ML)	IV	DOXORUBICIN
Doxorubicin	DOXORUBICIN VIAL 50 MG /25ML (25 MI	IV	ADRIM
Etoposide	ETOPOSIDE VIAL 100 MG (5 ML)	IV	ETOPOS
Etoposide	ETOPOSIDE VIAL 100 MG /5ML (5 ML)	IV	EPOSIN
Etoposide	ETOPOSIDE VIAL 100 MG /5ML (5 ML)	IV	FYTOSID
Etoposide	ETOPOSIDE VIAL 100 MG /5ML (5 ML)	IV	SEDOL
Mercaptopurine	MERCAPTOPURINE TAB 50 MG (25 tab)	tab	PURI-NETHOL
Mercaptopurine	MERCAPTOPURINE TAB 50 MG (100 tab)	tab	PURINETONE
MESNA	Mesna 400mg/4ml Injection	IV	
Methotrexate	METHOTREXATE VIAL 1000 MG (10 ML)	IV	EMTHEXATE
Methotrexate	METHOTREXATE VIAL 1000 MG (10 ML)	IV	ALLTREX
Methotrexate	METHOTREXATE VIAL 50 MG /2ML (2 ML	IV	BIOTREXATE
Methotrexate	METHOTREXATE VIAL 50 MG /2ML (2 ML	IV	EMTHEXATE
Methotrexate	METHOTREXATE VIAL 50 MG /2ML (2 ML	IV	ALLTREX
Methotrexate	METHOTREXATE TAB 2.5 MG	tab	BIOTREXATE
Methotrexate	METHOTREXATE TAB 2.5 MG	tab	EMTHEXATE
Methotrexate	METHOTREXATE TAB 2.5 MG	tab	METREX
Methotrexate	METHOTREXATE TAB 2.5 MG	tab	BIOTREXATE
Methotrexate	METHOTREXATE TAB 2.5 MG	tab	ONKOMET
Methotrexate	METHOTREXATE TAB 2.5 MG	tab	UNITREXATE
Methotrexate	METHOTREXATE TAB SC 2.5 MG	tab	METHOTREXA
Tioguanine	TIOGUANINE TAB 40 MG	tab	LANVIS
Vincristine	VINCRISTINE VIAL 1 MG /ML (1 ML)	IV	V.C.S.
Dexamethasone	Dexamethasone tab 0.5 mg	tab	
Leucovorin	Calcium folinate (leucovorin calcium) ste	IV	
Mitoxantrone	Mitoxantrone hydrochloride sterile sol 1(	IV	
Mitoxantrone	Mitoxantrone hydrochloride sterile sol 2(	IV	
Prednisolone	Prednisolone tab 5 mg	tab	

For peer review only

Source: Pharmaceutical Information Center Ministry of Public Health (2018 or 2019, see year columı
http://dmsic.moph.go.th/index/drugsearch/3

Exchange rates Official exchange rate (LCU per US$, period average)
Source: https://data.worldbank.org/indicator/PA.NUS.FCRF (accessed 29 May 2020)

Thailand	Inflation (%)		
2017		0.67	
2018		1.06	
2019		0.71	
2020		-1.06	*forecasted
2021		0.56	*forecasted

Source: https://www.statista.com/statistics/332274/inflation-rate-in-thailand/ (accessed 28 May 20

For peer review only

Manufacturer	strength	Units per pac	ng (or units)	Year	e local curre	ocal currenc
KYOWA HAKKO KO	10,000	1	10000	2018	1,675	THB
BAXTER HEALTHCA	1,000	1	1,000	2018	423	THB
BIOCHEM GES.M B	1,000	1	1,000	2018	200	THB
BAXTER HEALTHCA	200	10	2,000	2018	877	THB
BIOCHEM GES.M B	200	1	200	2018	50	THB
BAXTER HEALTHCA	500	1	500	2018	203	THB
BORYUNG	500	1	500	2018	110	THB
FRESENIUS	1,000	1	1,000	2018	879	THB
FRESENIUS	100	1	100	2018	129	THB
PFIZER INTER. COR	2,000	1	2,000	2018	2,825	THB
PFIZER INTER. COR	100	1	100	2018	205	THB
FRESENIUS	500	1	500	2018	508	THB
FRESENIUS	10	1	10	2018	118	THB
PHARMACHEMIE B.	50	1	50	2018	546	THB
PHARMACHEMIE B.	10	1	10	2018	139	THB
KHANDELWAL	50	1	50	2018	330	THB
FRESENIUS	50	1	50	2018	278	THB
TEVA INT'L	100	1	100	2018	189	THB
PHARMACHEMIE B.	100	1	100	2018	250	THB
FRESENIUS	100	1	100	2018	171	THB
VENUS REMEDIES	100	1	100	2018	160	THB
ASPEN PHARMACA	50	25	1,250	2018	856	THB
KOREA UNITED PH	50	100	5,000	2018	1,600	THB
	400	1	400	2019	8.7	RM
PHARMACHEMIE B.	1,000	1	1,000	2018	975	THB
NAPROD LIFE SCIEN	1,000	1	1,000	2018	945	THB
BIOCHEM GES.M B	50	1	50	2018	95	THB
PHARMACHEMIE B.	50	1	50	2018	110	THB
NAPROD LIFE SCIEN	50	1	50	2018	90	THB
BIOCHEM GES.MBH	2.5	100	250	2018	207	THB
PHARMACHEMIE B'	2.5	100	250	2018	321	THB
DAE HAN NEW PHA	2.5	100	250	2018	270	THB
BIOCHEM GES.MBH	2.5	50	125	2018	94	THB
KLAB	2.5	100	250	2018	240	THB
KENYAKU LTD	2.5	100	250	2018	247	THB
REMEDICA	2.5	100	250	2018	490	THB
ASPEN PHARMACA	40	25	1,000	2018	1,890	THB
BORYUNG	1	1	1	2019	140	THB
	0.5	1,000	500	2019	120.91	THB
	50	1	50	2019	352.03	THB
	10	1	10	2019	3,954.72	THB
	20	1	20	2019	6,364.36	THB
	5	500	2,500	2019	175.81	THB

For peer review only

n)

)20)

For peer review only

xchange rat	Price USD	Price per mg	Notes		price_mg_USD_201
32.31	52	0.005		Asparaginase	0.0049021
32.31	13	0.013		Cyclophosphamide	0.0094528
32.31	6	0.006		Cytarabine	0.0389176
32.31	27	0.014		Doxorubicin	0.2853954
32.31	2	0.008		Etoposide	0.0563812
32.31	6	0.013		Mercaptopurine	0.0147035
32.31	3	0.007		Methotrexate IV	0.0457699
32.31	27	0.027		Methotrexate tab	0.0328201
32.31	4	0.040		Mitoxantrone	10.33291
32.31	87	0.044		Tioguanine	0.0553137
32.31	6	0.064		Vincristine	4.058514
32.31	16	0.031			
32.31	4	0.364		Dexamethasone	0.0070022
32.31	17	0.338		Leucovorin	0.2038696
32.31	4	0.431		MESNA	0.0077992
32.31	10	0.204		Prednisolone	0.0020363
32.31	9	0.172			
32.31	6	0.059			
32.31	8	0.077			
32.31	5	0.053			
32.31	5	0.050			
32.31	26	0.021			
32.31	50	0.010			
3.9055	2	0.006	We could not find the price for MESNA in Thailand so we used the pric		
32.31	30	0.030			
32.31	29	0.029			
32.31	3	0.059			
32.31	3	0.068			
32.31	3	0.056			
32.31	6	0.026			
32.31	10	0.040			
32.31	8	0.033			
32.31	3	0.023			
32.31	7	0.030			
32.31	8	0.031			
32.31	15	0.061			
32.31	58	0.058			
31.05	5	4.515			
31.05	3.9	0.008			
31.05	11.3	0.227			
31.05	127.4	12.738			
31.05	205	10.249			
31.05	6	0.002			

For peer review only

For peer review only

price_mg_USD_2021
0.00543
0.01048
0.04313
0.31628
0.06248
0.01629
0.05072
0.03637
11.45239
0.06130
4.49822
0.00776
0.22596
0.00770
0.00226

:e published in the Philippines (https://dpri.doh.gov.ph/download/2019%20DPRI%20Fourth%20Editi

For peer review only

For peer review only

ion.pdf, accessed 10 May 2020)

For peer review only

Sensitivity analysis

1) Relapse 50%, relapse 0.5 SR: 0.5 HR	Diagnosed	Underdiagnosed
Total expenditure (US$)	1,015,410	1,701,286
Percentage of total health budget (%)	0.006%	0.010%
Asparaginase (smallest units)	5,632	9,436
Mercaptopurine (smallest units)	268,192	449,351

2) Incidence UB	Diagnosed	Underdiagnosed
Total expenditure (US$)	1,117,398	2,031,973
Percentage of total health budget (%)	0.007%	0.012%
Asparaginase (smallest units)	6,323	11,480
Mercaptopurine (smallest units)	306,075	557,666

3) Incidence UB, relapse 50%	Diagnosed	Underdiagnosed
Total expenditure (US$)	1,362,849	2,479,242
Percentage of total health budget (%)	0.008%	0.015%
Asparaginase (smallest units)	7,726	14,037
Mercaptopurine (smallest units)	331,720	604,396

4) Wastage, additional 20% total volume for	Diagnosed	Underdiagnosed
Total expenditure (US$)	938,391	1,572,239
Percentage of total health budget (%)	0.006%	0.009%
Asparaginase (smallest units)	5,529	9,263

4) Wastage, additional 40% total volume for	Diagnosed	Underdiagnosed
Total expenditure (US$)	1,061,828	1,779,053
Percentage of total health budget (%)	0.006%	0.010%
Asparaginase (smallest units)	6,450	10,807

For peer review only

Total health expenditure (in million constant (
17054.69

Additional expendi	Additional expenditure
123,439	206,817

Additional expendi	Additional expenditure
246,876	413,631

For peer review only

2017) US$)

For peer review only

STROBE Statement—checklist of items that should be included in reports of observational studies

	Item No	Recommendation	Page No
**Title and abstract**	1	(*a*) Indicate the study's design with a commonly used term in the title or the abstract	1,3
		(*b*) Provide in the abstract an informative and balanced summary of what was done and what was found	3
**Introduction**			
Background/rationale	2	Explain the scientific background and rationale for the investigation being reported	5-6
Objectives	3	State specific objectives, including any prespecified hypotheses	6
**Methods**			
Study design	4	Present key elements of study design early in the paper	6-7
Setting	5	Describe the setting, locations, and relevant dates, including periods of recruitment, exposure, follow-up, and data collection	6-7
Participants	6	(*a*) *Cohort study*—Give the eligibility criteria, and the sources and methods of selection of participants. Describe methods of follow-up<br>*Case-control study*—Give the eligibility criteria, and the sources and methods of case ascertainment and control selection. Give the rationale for the choice of cases and controls<br>*Cross-sectional study*—Give the eligibility criteria, and the sources and methods of selection of participants	6-7
		(*b*) *Cohort study*—For matched studies, give matching criteria and number of exposed and unexposed<br>*Case-control study*—For matched studies, give matching criteria and the number of controls per case	n/a
Variables	7	Clearly define all outcomes, exposures, predictors, potential confounders, and effect modifiers. Give diagnostic criteria, if applicable	8-9
Data sources/ measurement	8*	For each variable of interest, give sources of data and details of methods of assessment (measurement). Describe comparability of assessment methods if there is more than one group	6-8
Bias	9	Describe any efforts to address potential sources of bias	9
Study size	10	Explain how the study size was arrived at	6-7
Quantitative variables	11	Explain how quantitative variables were handled in the analyses. If applicable, describe which groupings were chosen and why	8-9
Statistical methods	12	(*a*) Describe all statistical methods, including those used to control for confounding	n/a
		(*b*) Describe any methods used to examine subgroups and interactions	n/a
		(*c*) Explain how missing data were addressed	n/a
		(*d*) *Cohort study*—If applicable, explain how loss to follow-up was addressed<br>*Case-control study*—If applicable, explain how matching of cases and controls was addressed<br>*Cross-sectional study*—If applicable, describe analytical methods taking account of sampling strategy	n/a
		(*e*) Describe any sensitivity analyses	8

Continued on next page

For peer review only - http://bmjopen.bmj.com/site/about/guidelines.xhtml

**Results**			
Participants	13*	(a) Report numbers of individuals at each stage of study—eg numbers potentially eligible, examined for eligibility, confirmed eligible, included in the study, completing follow-up, and analysed	9-10
		(b) Give reasons for non-participation at each stage	n/a
		(c) Consider use of a flow diagram	n/a
Descriptive data	14*	(a) Give characteristics of study participants (eg demographic, clinical, social) and information on exposures and potential confounders	9-10
		(b) Indicate number of participants with missing data for each variable of interest	n/a
		(c) *Cohort study*—Summarise follow-up time (eg, average and total amount)	n/a
Outcome data	15*	*Cohort study*—Report numbers of outcome events or summary measures over time	n/a
		Case-control study—Report numbers in each exposure category, or summary measures of exposure	n/a
		Cross-sectional study—Report numbers of outcome events or summary measures	9-10
Main results	16	(*a*) Give unadjusted estimates and, if applicable, confounder-adjusted estimates and their precision (eg, 95% confidence interval). Make clear which confounders were adjusted for and why they were included	9-10
		(*b*) Report category boundaries when continuous variables were categorized	9-10
		(*c*) If relevant, consider translating estimates of relative risk into absolute risk for a meaningful time period	n/a
Other analyses	17	Report other analyses done—eg analyses of subgroups and interactions, and sensitivity analyses	10
**Discussion**			
Key results	18	Summarise key results with reference to study objectives	10-11
Limitations	19	Discuss limitations of the study, taking into account sources of potential bias or imprecision. Discuss both direction and magnitude of any potential bias	11-12
Interpretation	20	Give a cautious overall interpretation of results considering objectives, limitations, multiplicity of analyses, results from similar studies, and other relevant evidence	11-12
Generalisability	21	Discuss the generalisability (external validity) of the study results	
**Other information**			
Funding	22	Give the source of funding and the role of the funders for the present study and, if applicable, for the original study on which the present article is based	13-14

*Give information separately for cases and controls in case-control studies and, if applicable, for exposed and unexposed groups in cohort and cross-sectional studies.

Note: An Explanation and Elaboration article discusses each checklist item and gives methodological background and published examples of transparent reporting. The STROBE checklist is best used in conjunction with this article (freely available on the Web sites of PLoS Medicine at http://www.plosmedicine.org/, Annals of Internal Medicine at http://www.annals.org/, and Epidemiology at http://www.epidem.com/). Information on the STROBE Initiative is available at www.strobe-statement.org.

For peer review only
